# Supplementary material for: Mendelian randomization study of self-reported long sleep duration, short sleep duration, and insomnia and cognitive function
Source: PLoS One. 2025 Aug 20;20(8):e0330782. doi: 10.1371/journal.pone.0330782 (PMC12367179; doi:10.1371/journal.pone.0330782)
Supplement: S1 Table — SNPs analysis of short sleep duration. SNPs analysis of insomnia. SNPs analysis of long sleep duration. MR-Egger test for MR analysis of sleep phenotypes and cognitive functions. Cochran’s Q statistics test for MR analysis of sleep phenotypes and cognitive functions. The results of MR analyses using the Outlier-corrected method in MR-PRESSO with the results of the original MR analyses. The no. of SNPs determined in this MR study. (DOCX) [file pone.0330782.s001.docx]

**S1 Table**

**Supplement Table I Detailed information for cognitive function tests and dementia-related outcomes**

| **PHENOTYPES** | **GWAS ID** | **Population** | **Category** | **Availability of data and materials/data availability** |
| --- | --- | --- | --- | --- |
| COGNITIVE FUNCTIONS: |  |  |  |  |
| Cognitive Performance | ebi-a-GCST006572 | European | Continuous | https://gwas.mrcieu.ac.uk/datasets/ebi-a-GCST006572/ |
| Fluid intelligence score (FIS) | ukb-b-5238 | European | Categorical Ordered | https://gwas.mrcieu.ac.uk/datasets/ukb-b-5238/ |
| Memory Performance | ukb-b-16872 | European | Continuous | https://gwas.mrcieu.ac.uk/datasets/ukb-b-16872/ |
| TM: Interval in trail 2 path | ukb-b-16311 | European | Continuous | https://gwas.mrcieu.ac.uk/datasets/ukb-b-16311/ |
| TM: Duration to complete trail 2 path | ukb-b-20140 | European | Continuous | https://gwas.mrcieu.ac.uk/datasets/ukb-b-20140/ |
| SDS: Number of correct matches | ukb-b-15625 | European | Continuous | https://gwas.mrcieu.ac.uk/datasets/ukb-b-15625/ |
| SDS: Number of matches attempted | ukb-b-1806 | European | Continuous | https://gwas.mrcieu.ac.uk/datasets/ukb-b-1806/ |
| SDS: Duration to entering value | ukb-b-19585 | European | Continuous | https://gwas.mrcieu.ac.uk/datasets/ukb-b-19585/ |
| PM: Number of incorrect matches | ukb-b-20498 | European | Continuous | https://gwas.mrcieu.ac.uk/datasets/ukb-b-20498/ |
| PM: Time to complete round | ukb-b-13823 | European | Continuous | https://gwas.mrcieu.ac.uk/datasets/ukb-b-13823/ |
| Reaction Time | ieu-a-1066 | European | Continuous | https://gwas.mrcieu.ac.uk/datasets/ieu-a-1066/ |
| DEMENTIA-RELATED OUTCOMES: |  |  |  |  |
| Alzheimer's Disease | finn-b-G6_AD_WIDE | European | Binary | https://gwas.mrcieu.ac.uk/datasets/finn-b-G6_AD_WIDE/ |
| Lewy body dementia | ebi-a-GCST90001390 | European | Binary | https://gwas.mrcieu.ac.uk/datasets/ebi-a-GCST90001390/ |
| Vascular dementia | finn-b-VD_MI | European | Binary | https://gwas.mrcieu.ac.uk/datasets/finn-b-VD_MI/ |
| Frontotemporal dementia | ieu-b-43 | European | Binary | https://gwas.mrcieu.ac.uk/datasets/ieu-b-43/ |

*Notes.The GWAS summary statistics of cognitive function tests and dementia-related outcomes used in this MR study are available in OpenGWAS*

**Supplement Table II SNPs analysis of short sleep duration**

| SNP | Chromosome | Position | Effect allele | Other allele | MAF | EAF | Beta | SE | P value |
| --- | --- | --- | --- | --- | --- | --- | --- | --- | --- |
| rs2863957 | 2 | 114089551 | C | A | 0.22 | 0.78 | 0.0545 | 0.0073 | 2.60×10-18 |
| rs13107325 | 4 | 103188709 | T | C | 0.08 | 0.08 | 0.0751 | 0.011 | 2.50×10-13 |
| rs1229762 | 7 | 114218582 | T | C | 0.34 | 0.67 | 0.0373 | 0.0064 | 1.10×10-12 |
| rs1380703 | 2 | 57941287 | G | A | 0.38 | 0.38 | 0.0354 | 0.0059 | 1.60×10-11 |
| rs12963463 | 18 | 53099093 | C | T | 0.3 | 0.3 | 0.0286 | 0.0065 | 1.90×10-11 |
| rs75539574 | 2 | 58871658 | A | C | 0.09 | 0.92 | 0.045 | 0.0108 | 8.40×10-11 |
| rs17388803 | 15 | 48027204 | C | A | 0.11 | 0.11 | 0.0526 | 0.0098 | 6.50×10-10 |
| rs4585442 | 5 | 135508381 | G | A | 0.31 | 0.31 | 0.0305 | 0.006 | 8.10×10-10 |
| rs1607227 | 11 | 28808617 | G | T | 0.3 | 0.71 | 0.0305 | 0.0065 | 1.50×10-9 |
| rs2820313 | 1 | 201870221 | G | A | 0.34 | 0.34 | 0.0305 | 0.006 | 2.30×10-9 |
| rs17005118 | 4 | 82288564 | A | G | 0.27 | 0.27 | 0.0296 | 0.0065 | 2.50×10-9 |
| rs5757675 | 22 | 39838892 | G | T | 0.26 | 0.26 | 0.0344 | 0.0069 | 2.70×10-9 |
| rs12567114 | 1 | 98527951 | G | A | 0.28 | 0.73 | 0.0363 | 0.0064 | 4.10×10-9 |
| rs2186122 | 1 | 66470206 | T | A | 0.44 | 0.56 | 0.0237 | 0.006 | 4.80×10-9 |
| rs11763750 | 7 | 2080114 | G | A | 0.19 | 0.81 | 0.0354 | 0.0079 | 5.10×10-9 |
| rs12518468 | 5 | 7249696 | C | T | 0.33 | 0.33 | 0.0315 | 0.0065 | 8.50×10-9 |
| rs9367621 | 6 | 55040290 | T | A | 0.43 | 0.43 | 0.0237 | 0.0055 | 1.60×10-8 |
| rs3776864 | 5 | 102327868 | A | C | 0.33 | 0.67 | 0.0315 | 0.0065 | 1.70×10-8 |
| rs60882754 | 8 | 52886619 | A | T | 0.06 | 0.94 | 0.0554 | 0.0122 | 1.80×10-8 |
| rs59779556 | 16 | 56227965 | T | G | 0.45 | 0.55 | 0.0247 | 0.006 | 2.00×10-8 |
| rs2014830 | 3 | 50172397 | C | T | 0.3 | 0.7 | 0.0296 | 0.0065 | 2.70×10-8 |
| rs205024 | 17 | 11227352 | C | T | 0.38 | 0.62 | 0.0305 | 0.0065 | 2.70×10-8 |
| rs12661667 | 6 | 41792545 | T | C | 0.26 | 0.26 | 0.0276 | 0.0065 | 2.80×10-8 |
| rs7939345 | 11 | 47980568 | T | G | 0.21 | 0.21 | 0.0354 | 0.0074 | 4.00×10-8 |
| rs9321171 | 6 | 129848635 | C | T | 0.46 | 0.54 | 0.0315 | 0.006 | 4.20×10-8 |
| rs7524118 | 1 | 34736052 | C | T | 0.29 | 0.71 | 0.0296 | 0.0065 | 4.90×10-8 |

**Supplement Table III SNPs analysis of insomnia**

| SNP | Chromosome | Position | Effect allele | Other allele | MAF | EAF | Beta | SE | P value |
| --- | --- | --- | --- | --- | --- | --- | --- | --- | --- |
| rs113851554 | 2 | 66750564 | T | G | 0.05 | 0.05 | 0.2062 | 0.014 | 1.56×10-51 |
| rs9527083 | 13 | 53991125 | A | G | 0.33 | 0.67 | -0.0758 | 0.006 | 1.61×10-32 |
| rs10947428 | 6 | 33647058 | T | C | 0.21 | 0.79 | -0.0683 | 0.007 | 9.06×10-21 |
| rs6119267 | 20 | 31163914 | C | G | 0.31 | 0.69 | -0.0598 | 0.006 | 2.32×10-20 |
| rs62158170 | 2 | 114082175 | A | G | 0.21 | 0.79 | 0.0658 | 0.007 | 1.20×10-19 |
| rs9394502 | 6 | 38452503 | T | C | 0.33 | 0.33 | -0.0545 | 0.006 | 7.76×10-18 |
| rs55972276 | 5 | 135653737 | A | C | 0.14 | 0.14 | 0.0733 | 0.009 | 4.19×10-17 |
| rs2431108 | 5 | 103947968 | T | C | 0.33 | 0.67 | -0.0534 | 0.006 | 7.83×10-17 |
| rs7566062 | 2 | 66972843 | T | C | 0.22 | 0.22 | 0.0592 | 0.007 | 1.37×10-16 |
| rs8180817 | 7 | 114047542 | C | G | 0.43 | 0.43 | -0.0492 | 0.006 | 1.83×10-16 |
| rs118166957 | 9 | 8858043 | T | C | 0.16 | 0.16 | 0.0677 | 0.008 | 1.95×10-16 |
| rs13135092 | 4 | 103198082 | A | G | 0.08 | 0.92 | -0.0888 | 0.011 | 2.53×10-16 |
| rs35322724 | 16 | 77137324 | A | C | 0.42 | 0.58 | 0.0488 | 0.006 | 3.75×10-16 |
| rs4702 | 15 | 91426560 | A | G | 0.44 | 0.56 | -0.0481 | 0.006 | 6.78×10-16 |
| rs2491124 | 13 | 53784083 | T | C | 0.42 | 0.58 | 0.0488 | 0.006 | 8.81×10-16 |
| rs16903122 | 5 | 87693561 | T | C | 0.25 | 0.25 | 0.0554 | 0.007 | 9.04×10-16 |
| rs77641763 | 9 | 140265782 | T | C | 0.12 | 0.12 | 0.0714 | 0.009 | 6.53×10-15 |
| rs1927902 | 9 | 120518991 | T | C | 0.25 | 0.25 | 0.0526 | 0.007 | 1.15×10-14 |
| rs62264767 | 3 | 117642005 | A | C | 0.15 | 0.85 | 0.0649 | 0.008 | 1.63×10-14 |
| rs1620977 | 1 | 72729142 | A | G | 0.27 | 0.27 | 0.0516 | 0.007 | 2.27×10-14 |
| rs1015438 | 16 | 51177517 | A | G | 0.19 | 0.19 | 0.0583 | 0.008 | 2.51×10-14 |
| rs17643634 | 8 | 91650818 | T | C | 0.17 | 0.17 | -0.0598 | 0.008 | 1.34×10-13 |
| rs28582096 | 12 | 123856998 | A | G | 0.21 | 0.21 | -0.0545 | 0.007 | 1.74×10-13 |
| rs694786 | 3 | 173112907 | T | C | 0.46 | 0.46 | -0.044 | 0.006 | 1.97×10-13 |
| rs2815757 | 1 | 72764289 | T | C | 0.19 | 0.81 | 0.0554 | 0.008 | 2.24×10-13 |
| rs78206187 | 2 | 67022234 | A | G | 0.06 | 0.94 | -0.0943 | 0.013 | 2.96×10-13 |
| rs12912299 | 15 | 38897857 | T | C | 0.49 | 0.49 | -0.0429 | 0.006 | 4.42×10-13 |
| rs2903385 | 4 | 106094427 | A | G | 0.48 | 0.48 | 0.0431 | 0.006 | 4.53×10-13 |
| rs7214267 | 17 | 43157709 | A | G | 0.42 | 0.58 | -0.044 | 0.006 | 5.09×10-13 |
| rs314281 | 6 | 105400605 | T | C | 0.45 | 0.45 | -0.0429 | 0.006 | 6.03×10-13 |
| rs11605348 | 11 | 47606483 | A | G | 0.35 | 0.35 | -0.045 | 0.006 | 7.01×10-13 |
| rs4981170 | 14 | 33412996 | A | G | 0.19 | 0.19 | -0.0545 | 0.008 | 7.33×10-13 |
| rs670501 | 7 | 108625185 | T | C | 0.21 | 0.21 | 0.0526 | 0.007 | 7.40×10-13 |
| rs11126082 | 2 | 66789341 | C | G | 0.44 | 0.44 | -0.0429 | 0.006 | 8.26×10-13 |
| rs9931543 | 16 | 56128782 | T | C | 0.26 | 0.74 | 0.0478 | 0.007 | 1.11×10-12 |
| rs524859 | 11 | 66041079 | A | G | 0.36 | 0.36 | -0.044 | 0.006 | 1.48×10-12 |
| rs72657797 | 4 | 90820809 | T | C | 0.18 | 0.18 | -0.0555 | 0.008 | 1.52×10-12 |
| rs60565673 | 18 | 52906830 | T | G | 0.38 | 0.62 | -0.0429 | 0.006 | 1.59×10-12 |
| rs10761240 | 9 | 96361922 | A | G | 0.4 | 0.4 | -0.0429 | 0.006 | 2.12×10-12 |
| rs66674044 | 16 | 19904344 | A | T | 0.14 | 0.86 | -0.0598 | 0.009 | 2.18×10-12 |
| rs12666306 | 7 | 115082406 | A | G | 0.5 | 0.5 | 0.0421 | 0.006 | 2.24×10-12 |
| rs10800992 | 1 | 190900576 | T | C | 0.44 | 0.44 | 0.0421 | 0.006 | 3.84×10-12 |
| rs1031654 | 13 | 54382035 | A | C | 0.2 | 0.8 | -0.0513 | 0.007 | 3.88×10-12 |
| rs10947690 | 6 | 37631768 | A | G | 0.26 | 0.74 | -0.0471 | 0.007 | 4.04×10-12 |
| rs9889282 | 17 | 50259142 | A | C | 0.39 | 0.61 | -0.0419 | 0.006 | 4.70×10-12 |
| rs56133505 | 11 | 72348039 | A | G | 0.46 | 0.54 | 0.0411 | 0.006 | 5.59×10-12 |
| rs7044885 | 9 | 81739348 | C | G | 0.44 | 0.44 | -0.0408 | 0.006 | 5.67×10-12 |
| rs3774751 | 3 | 50209053 | T | G | 0.46 | 0.46 | -0.0408 | 0.006 | 7.32×10-12 |
| rs61921611 | 12 | 66367726 | T | C | 0.31 | 0.69 | -0.044 | 0.006 | 7.84×10-12 |
| rs715338 | 15 | 57215867 | A | G | 0.42 | 0.58 | 0.0411 | 0.006 | 7.85×10-12 |
| rs830716 | 16 | 12323509 | C | G | 0.29 | 0.71 | 0.045 | 0.007 | 8.68×10-12 |
| rs13010288 | 2 | 51824512 | T | G | 0.13 | 0.13 | -0.0598 | 0.009 | 9.26×10-12 |
| rs12983032 | 19 | 5073447 | A | G | 0.34 | 0.34 | -0.0429 | 0.006 | 1.07×10-11 |
| rs8180457 | 5 | 107209814 | T | C | 0.16 | 0.16 | -0.0555 | 0.008 | 1.12×10-11 |
| rs6545798 | 2 | 60521311 | A | T | 0.41 | 0.41 | -0.0408 | 0.006 | 1.19×10-11 |
| rs908668 | 19 | 56134038 | T | C | 0.21 | 0.21 | 0.0497 | 0.007 | 1.41×10-11 |
| rs79693059 | 11 | 72340686 | C | G | 0.08 | 0.92 | -0.0726 | 0.011 | 1.61×10-11 |
| rs10865954 | 3 | 49211989 | T | C | 0.33 | 0.33 | 0.0421 | 0.006 | 1.92×10-11 |
| rs9373590 | 6 | 101212001 | A | T | 0.49 | 0.51 | 0.0402 | 0.006 | 2.18×10-11 |
| rs12991815 | 2 | 68071990 | C | G | 0.42 | 0.42 | 0.0402 | 0.006 | 3.02×10-11 |
| rs61765555 | 1 | 73957815 | T | C | 0.26 | 0.26 | -0.045 | 0.007 | 4.00×10-11 |
| rs12310246 | 12 | 84700945 | A | G | 0.25 | 0.25 | 0.045 | 0.007 | 4.74×10-11 |
| rs55772859 | 2 | 208042581 | A | C | 0.31 | 0.31 | 0.0421 | 0.006 | 4.82×10-11 |
| rs6808140 | 3 | 10581380 | T | C | 0.49 | 0.51 | 0.0392 | 0.006 | 5.35×10-11 |
| rs67501351 | 16 | 20006745 | C | G | 0.25 | 0.75 | 0.045 | 0.007 | 5.36×10-11 |
| rs2286729 | 12 | 6873818 | A | G | 0.09 | 0.09 | 0.0695 | 0.011 | 5.37×10-11 |
| rs11803128 | 1 | 190060095 | A | G | 0.35 | 0.65 | -0.0408 | 0.006 | 6.85×10-11 |
| rs28611339 | 8 | 10170037 | T | G | 0.13 | 0.13 | 0.0583 | 0.009 | 8.46×10-11 |
| rs10502966 | 18 | 50748499 | A | G | 0.42 | 0.58 | -0.0387 | 0.006 | 8.54×10-11 |
| rs35110063 | 3 | 43066558 | A | G | 0.43 | 0.43 | 0.0392 | 0.006 | 8.82×10-11 |
| rs2792990 | 9 | 125621610 | C | G | 0.14 | 0.86 | 0.0545 | 0.008 | 1.15×10-10 |
| rs566673 | 11 | 66401373 | T | G | 0.46 | 0.54 | -0.0387 | 0.006 | 1.18×10-10 |
| rs6888135 | 5 | 141254063 | A | C | 0.5 | 0.5 | 0.0383 | 0.006 | 1.21×10-10 |
| rs6562066 | 13 | 60532796 | T | C | 0.37 | 0.37 | 0.0392 | 0.006 | 1.38×10-10 |
| rs6967168 | 7 | 132672192 | T | G | 0.25 | 0.75 | -0.044 | 0.007 | 1.39×10-10 |
| rs4643373 | 17 | 47123423 | T | C | 0.3 | 0.7 | 0.0411 | 0.007 | 1.58×10-10 |
| rs12187443 | 5 | 102660400 | T | C | 0.33 | 0.67 | 0.0402 | 0.006 | 1.64×10-10 |
| rs1861412 | 2 | 58893065 | A | G | 0.43 | 0.43 | 0.0383 | 0.006 | 1.67×10-10 |
| rs2389631 | 13 | 96932868 | A | C | 0.33 | 0.67 | -0.0398 | 0.006 | 2.03×10-10 |
| rs116466468 | 2 | 159137557 | T | C | 0.24 | 0.76 | 0.044 | 0.007 | 2.11×10-10 |
| rs1064939 | 11 | 118396331 | A | T | 0.02 | 0.98 | 0.1302 | 0.02 | 2.16×10-10 |
| rs4238755 | 16 | 52746089 | A | C | 0.26 | 0.26 | -0.0429 | 0.007 | 2.30×10-10 |
| rs17223714 | 5 | 50492629 | A | G | 0.21 | 0.79 | 0.0459 | 0.007 | 2.44×10-10 |
| rs1038093 | 15 | 74012409 | T | C | 0.37 | 0.63 | 0.0392 | 0.006 | 2.47×10-10 |
| rs224029 | 10 | 64519299 | T | C | 0.4 | 0.4 | -0.0387 | 0.006 | 2.51×10-10 |
| rs3902952 | 16 | 61647589 | T | C | 0.19 | 0.19 | 0.0478 | 0.008 | 2.55×10-10 |
| rs56097173 | 2 | 44262449 | T | C | 0.32 | 0.68 | 0.0402 | 0.006 | 2.69×10-10 |
| rs8076183 | 17 | 61024696 | T | C | 0.45 | 0.45 | -0.0377 | 0.006 | 2.75×10-10 |
| rs2089358 | 1 | 37194103 | T | C | 0.3 | 0.7 | -0.0408 | 0.007 | 2.75×10-10 |
| rs671985 | 8 | 60914783 | A | G | 0.45 | 0.45 | -0.0377 | 0.006 | 2.79×10-10 |
| rs12251016 | 10 | 21821918 | A | T | 0.34 | 0.66 | -0.0387 | 0.006 | 3.89×10-10 |
| rs4592425 | 11 | 62697813 | T | G | 0.3 | 0.7 | 0.0402 | 0.006 | 4.31×10-10 |
| rs12790660 | 11 | 57667222 | T | C | 0.32 | 0.68 | -0.0398 | 0.006 | 4.49×10-10 |
| rs152555 | 5 | 106849674 | A | G | 0.15 | 0.85 | -0.0523 | 0.008 | 4.83×10-10 |
| rs2398144 | 16 | 56352854 | A | C | 0.39 | 0.39 | 0.0383 | 0.006 | 5.09×10-10 |
| rs11838830 | 13 | 60362013 | A | G | 0.06 | 0.94 | -0.0801 | 0.013 | 5.20×10-10 |
| rs823247 | 2 | 2850540 | T | C | 0.48 | 0.48 | -0.0367 | 0.006 | 5.25×10-10 |
| rs73671843 | 7 | 3520024 | A | G | 0.13 | 0.13 | -0.0555 | 0.009 | 5.49×10-10 |
| rs17005118 | 4 | 82288564 | A | G | 0.26 | 0.26 | 0.0421 | 0.007 | 6.13×10-10 |
| rs1064213 | 2 | 198950240 | A | G | 0.48 | 0.48 | -0.0367 | 0.006 | 6.41×10-10 |
| rs4767645 | 12 | 118385788 | T | G | 0.46 | 0.46 | -0.0367 | 0.006 | 6.47×10-10 |
| rs6019663 | 20 | 47774512 | T | C | 0.29 | 0.29 | 0.0402 | 0.007 | 6.47×10-10 |
| rs3131638 | 6 | 31475127 | A | G | 0.23 | 0.23 | -0.044 | 0.007 | 7.88×10-10 |
| rs984306 | 2 | 66817402 | T | C | 0.25 | 0.75 | -0.0429 | 0.007 | 7.94×10-10 |
| rs4502882 | 5 | 153093998 | T | C | 0.34 | 0.66 | -0.0387 | 0.006 | 7.96×10-10 |
| rs940780 | 7 | 3323848 | T | C | 0.36 | 0.36 | 0.0383 | 0.006 | 8.50×10-10 |
| rs1530938 | 2 | 236900633 | A | G | 0.44 | 0.44 | 0.0363 | 0.006 | 8.82×10-10 |
| rs1264419 | 6 | 30576781 | C | G | 0.49 | 0.51 | 0.0363 | 0.006 | 8.91×10-10 |
| rs3184470 | 16 | 715164 | A | G | 0.35 | 0.35 | -0.0377 | 0.006 | 9.73×10-10 |
| rs1147852 | 6 | 147980909 | A | G | 0.31 | 0.31 | 0.0392 | 0.006 | 9.94×10-10 |
| rs72899452 | 11 | 45415577 | T | C | 0.06 | 0.06 | 0.0742 | 0.012 | 1.00×10-9 |
| rs6756610 | 2 | 147480394 | C | G | 0.37 | 0.63 | 0.0373 | 0.006 | 1.14×10-9 |
| rs62068188 | 17 | 2400876 | T | C | 0.17 | 0.83 | 0.0488 | 0.008 | 1.18×10-9 |
| rs6702604 | 1 | 107190062 | A | G | 0.42 | 0.58 | -0.0367 | 0.006 | 1.30×10-9 |
| rs2388840 | 6 | 99598756 | A | G | 0.42 | 0.58 | -0.0367 | 0.006 | 1.37×10-9 |
| rs9540729 | 13 | 66947124 | A | T | 0.48 | 0.48 | 0.0363 | 0.006 | 1.40×10-9 |
| rs324017 | 12 | 57487814 | A | C | 0.29 | 0.29 | 0.0392 | 0.007 | 1.61×10-9 |
| rs12520974 | 5 | 61514611 | T | C | 0.48 | 0.48 | -0.0356 | 0.006 | 1.69×10-9 |
| rs4790076 | 17 | 2243628 | T | C | 0.17 | 0.17 | 0.0478 | 0.008 | 1.76×10-9 |
| rs62429521 | 6 | 140324582 | A | C | 0.15 | 0.15 | 0.0507 | 0.008 | 1.78×10-9 |
| rs11090039 | 22 | 41496800 | A | G | 0.29 | 0.29 | 0.0392 | 0.007 | 1.82×10-9 |
| rs6734957 | 2 | 42813247 | T | G | 0.24 | 0.24 | -0.0419 | 0.007 | 1.82×10-9 |
| rs6465151 | 7 | 88310899 | T | C | 0.11 | 0.11 | 0.0564 | 0.009 | 1.90×10-9 |
| rs2221119 | 11 | 88598444 | C | G | 0.44 | 0.44 | 0.0363 | 0.006 | 2.00×10-9 |
| rs12605642 | 18 | 31313965 | T | G | 0.49 | 0.49 | 0.0354 | 0.006 | 2.13×10-9 |
| rs11149313 | 13 | 85294881 | A | G | 0.27 | 0.73 | 0.0402 | 0.007 | 2.38×10-9 |
| rs2598293 | 7 | 133989882 | T | C | 0.48 | 0.48 | 0.0354 | 0.006 | 2.48×10-9 |
| rs7615602 | 3 | 18718055 | C | G | 0.27 | 0.27 | -0.0398 | 0.007 | 2.59×10-9 |
| rs4709655 | 6 | 163280204 | T | C | 0.12 | 0.12 | -0.0545 | 0.009 | 3.09×10-9 |
| rs11679943 | 2 | 77724624 | A | G | 0.35 | 0.35 | 0.0373 | 0.006 | 3.16×10-9 |
| rs214934 | 11 | 17193475 | A | T | 0.31 | 0.31 | -0.0377 | 0.006 | 3.16×10-9 |
| rs34214423 | 16 | 52303107 | A | C | 0.19 | 0.81 | 0.045 | 0.008 | 3.18×10-9 |
| rs71575448 | 5 | 106918329 | A | G | 0.14 | 0.86 | 0.0507 | 0.009 | 3.38×10-9 |
| rs492858 | 3 | 155432229 | T | C | 0.08 | 0.08 | -0.0661 | 0.011 | 3.46×10-9 |
| rs72773790 | 9 | 139109080 | T | C | 0.33 | 0.67 | 0.0373 | 0.006 | 3.71×10-9 |
| rs7040224 | 9 | 134886837 | A | G | 0.32 | 0.32 | 0.0373 | 0.006 | 4.24×10-9 |
| rs34967082 | 2 | 215382654 | A | G | 0.41 | 0.41 | 0.0354 | 0.006 | 4.34×10-9 |
| rs45453598 | 16 | 52637892 | A | T | 0.17 | 0.17 | 0.0469 | 0.008 | 4.42×10-9 |
| rs35539975 | 5 | 91607148 | A | G | 0.22 | 0.78 | 0.0421 | 0.007 | 4.49×10-9 |
| rs62194948 | 2 | 239222376 | C | G | 0.28 | 0.28 | 0.0392 | 0.007 | 4.64×10-9 |
| rs6589988 | 11 | 99126016 | A | G | 0.32 | 0.68 | -0.0377 | 0.006 | 4.70×10-9 |
| rs37445 | 5 | 106899684 | A | G | 0.39 | 0.39 | -0.0356 | 0.006 | 4.88×10-9 |
| rs10758593 | 9 | 4292083 | A | G | 0.4 | 0.4 | -0.0356 | 0.006 | 4.90×10-9 |
| rs12454003 | 18 | 26315799 | C | G | 0.48 | 0.48 | -0.0346 | 0.006 | 4.94×10-9 |
| rs4664299 | 2 | 160570033 | T | C | 0.23 | 0.23 | -0.0408 | 0.007 | 4.95×10-9 |
| rs7599697 | 2 | 239231477 | T | C | 0.36 | 0.36 | -0.0367 | 0.006 | 5.00×10-9 |
| rs11588755 | 1 | 57819204 | A | G | 0.48 | 0.52 | -0.0346 | 0.006 | 5.14×10-9 |
| rs7402939 | 15 | 99183876 | T | C | 0.38 | 0.38 | -0.0356 | 0.006 | 5.19×10-9 |
| rs7625896 | 3 | 44062561 | A | G | 0.35 | 0.65 | 0.0363 | 0.006 | 5.28×10-9 |
| rs871994 | 8 | 35190619 | A | C | 0.44 | 0.44 | 0.0354 | 0.006 | 5.50×10-9 |
| rs9316619 | 13 | 53978628 | T | C | 0.18 | 0.82 | 0.0459 | 0.008 | 5.50×10-9 |
| rs1289939 | 1 | 117944435 | T | C | 0.23 | 0.23 | -0.0408 | 0.007 | 6.00×10-9 |
| rs1536053 | 13 | 111982291 | T | C | 0.32 | 0.32 | -0.0377 | 0.006 | 6.04×10-9 |
| rs4788203 | 16 | 29978827 | A | G | 0.43 | 0.43 | -0.0346 | 0.006 | 6.32×10-9 |
| rs7475916 | 10 | 77771194 | C | G | 0.35 | 0.35 | -0.0367 | 0.006 | 6.70×10-9 |
| rs701394 | 5 | 80296487 | A | G | 0.36 | 0.64 | -0.0356 | 0.006 | 6.83×10-9 |
| rs12614369 | 2 | 66792109 | A | G | 0.18 | 0.82 | 0.044 | 0.008 | 7.21×10-9 |
| rs7432782 | 3 | 48941551 | T | C | 0.04 | 0.96 | -0.0834 | 0.014 | 7.42×10-9 |
| rs2838787 | 21 | 46539725 | A | G | 0.39 | 0.39 | -0.0356 | 0.006 | 7.65×10-9 |
| rs874168 | 8 | 30849450 | T | C | 0.47 | 0.53 | 0.0344 | 0.006 | 7.95×10-9 |
| rs10944696 | 6 | 94498850 | A | G | 0.3 | 0.3 | -0.0377 | 0.007 | 7.99×10-9 |
| rs10898940 | 11 | 73455292 | A | C | 0.48 | 0.52 | 0.0344 | 0.006 | 8.09×10-9 |
| rs12030482 | 1 | 96961268 | A | T | 0.22 | 0.22 | 0.0411 | 0.007 | 8.16×10-9 |
| rs4090240 | 9 | 77118987 | T | C | 0.28 | 0.28 | -0.0387 | 0.007 | 8.46×10-9 |
| rs1167132 | 12 | 43484487 | T | C | 0.39 | 0.39 | 0.0354 | 0.006 | 8.73×10-9 |
| rs176644 | 15 | 89913632 | T | G | 0.4 | 0.4 | 0.0354 | 0.006 | 9.49×10-9 |
| rs728017 | 6 | 124292594 | A | G | 0.39 | 0.39 | -0.0346 | 0.006 | 9.51×10-9 |
| rs2030672 | 7 | 21687925 | C | G | 0.44 | 0.56 | 0.0344 | 0.006 | 1.10×10-8 |
| rs6457796 | 6 | 34828553 | T | C | 0.27 | 0.73 | -0.0387 | 0.007 | 1.12×10-8 |
| rs7992992 | 13 | 54721699 | A | G | 0.13 | 0.13 | 0.0507 | 0.009 | 1.15×10-8 |
| rs11119409 | 1 | 210293333 | T | C | 0.41 | 0.59 | -0.0346 | 0.006 | 1.19×10-8 |
| rs11650304 | 17 | 46035001 | C | G | 0.07 | 0.93 | 0.0667 | 0.012 | 1.23×10-8 |
| rs5877 | 1 | 173878862 | T | C | 0.33 | 0.67 | 0.0363 | 0.006 | 1.23×10-8 |
| rs4858708 | 3 | 25154112 | A | T | 0.47 | 0.53 | -0.0336 | 0.006 | 1.23×10-8 |
| rs72820274 | 2 | 104412924 | A | G | 0.42 | 0.42 | 0.0344 | 0.006 | 1.28×10-8 |
| rs11756035 | 6 | 18843810 | C | G | 0.13 | 0.13 | 0.0507 | 0.009 | 1.29×10-8 |
| rs1731951 | 7 | 137075847 | A | T | 0.44 | 0.44 | -0.0346 | 0.006 | 1.36×10-8 |
| rs62301574 | 4 | 22050165 | C | G | 0.2 | 0.8 | -0.0419 | 0.007 | 1.37×10-8 |
| rs1357685 | 7 | 109200331 | T | C | 0.47 | 0.47 | 0.0334 | 0.006 | 1.39×10-8 |
| rs73163783 | 3 | 117602144 | T | C | 0.28 | 0.72 | -0.0377 | 0.007 | 1.39×10-8 |
| rs7571486 | 2 | 176473295 | A | G | 0.25 | 0.25 | -0.0387 | 0.007 | 1.40×10-8 |
| rs138678612 | 6 | 30932223 | A | G | 0.02 | 0.98 | -0.1165 | 0.02 | 1.41×10-8 |
| rs10825503 | 10 | 57177470 | T | G | 0.49 | 0.49 | 0.0334 | 0.006 | 1.43×10-8 |
| rs6606731 | 12 | 109982578 | A | T | 0.19 | 0.19 | 0.0431 | 0.008 | 1.51×10-8 |
| rs521484 | 7 | 49894349 | A | G | 0.23 | 0.77 | -0.0398 | 0.007 | 1.53×10-8 |
| rs4588900 | 8 | 73890425 | A | G | 0.48 | 0.52 | 0.0334 | 0.006 | 1.57×10-8 |
| rs75452188 | 2 | 67134426 | A | G | 0.12 | 0.88 | 0.0516 | 0.009 | 1.58×10-8 |
| rs2216427 | 3 | 180785697 | C | G | 0.35 | 0.65 | 0.0354 | 0.006 | 1.60×10-8 |
| rs17083297 | 5 | 92995477 | A | C | 0.18 | 0.18 | -0.044 | 0.008 | 1.60×10-8 |
| rs10928256 | 2 | 146458738 | T | C | 0.42 | 0.42 | 0.0344 | 0.006 | 1.61×10-8 |
| rs910187 | 20 | 45841052 | A | G | 0.37 | 0.37 | -0.0346 | 0.006 | 1.63×10-8 |
| rs34490907 | 17 | 26933741 | C | G | 0.11 | 0.89 | 0.0535 | 0.009 | 1.76×10-8 |
| rs10756571 | 9 | 14534505 | T | C | 0.31 | 0.69 | 0.0363 | 0.006 | 1.80×10-8 |
| rs7168238 | 15 | 66709386 | C | G | 0.07 | 0.07 | 0.0639 | 0.011 | 1.80×10-8 |
| rs10955647 | 8 | 114154187 | T | G | 0.47 | 0.53 | 0.0334 | 0.006 | 1.84×10-8 |
| rs1519102 | 2 | 66677816 | C | G | 0.31 | 0.69 | -0.0367 | 0.006 | 1.90×10-8 |
| rs12924275 | 16 | 9191790 | T | C | 0.27 | 0.27 | 0.0383 | 0.007 | 1.93×10-8 |
| rs13138995 | 4 | 148987430 | A | G | 0.39 | 0.39 | 0.0344 | 0.006 | 1.97×10-8 |
| rs16990210 | 4 | 34720226 | T | C | 0.15 | 0.85 | -0.046 | 0.008 | 1.97×10-8 |
| rs34036083 | 2 | 66815719 | T | C | 0.34 | 0.66 | -0.0356 | 0.006 | 2.07×10-8 |
| rs1937447 | 1 | 66358242 | C | G | 0.24 | 0.76 | -0.0387 | 0.007 | 2.08×10-8 |
| rs6978112 | 7 | 1966841 | T | C | 0.41 | 0.41 | 0.0344 | 0.006 | 2.11×10-8 |
| rs429358 | 19 | 45411941 | T | C | 0.15 | 0.85 | 0.0459 | 0.008 | 2.13×10-8 |
| rs2364921 | 3 | 158522463 | T | C | 0.47 | 0.47 | -0.0336 | 0.006 | 2.13×10-8 |
| rs1567084 | 3 | 71435955 | A | G | 0.5 | 0.5 | 0.0334 | 0.006 | 2.14×10-8 |
| rs17025198 | 3 | 88001713 | A | G | 0.2 | 0.2 | 0.0411 | 0.007 | 2.19×10-8 |
| rs6601080 | 5 | 179511043 | A | G | 0.32 | 0.68 | 0.0354 | 0.006 | 2.21×10-8 |
| rs667730 | 11 | 83277325 | T | C | 0.42 | 0.58 | 0.0334 | 0.006 | 2.26×10-8 |
| rs1580173 | 3 | 107955515 | A | G | 0.44 | 0.56 | 0.0334 | 0.006 | 2.28×10-8 |
| rs62213452 | 2 | 210380152 | T | G | 0.28 | 0.28 | 0.0373 | 0.007 | 2.39×10-8 |
| rs742760 | 20 | 50985290 | A | T | 0.18 | 0.82 | 0.0431 | 0.008 | 2.48×10-8 |
| rs2447094 | 17 | 2294048 | A | C | 0.47 | 0.47 | -0.0336 | 0.006 | 2.50×10-8 |
| rs11001276 | 10 | 76825638 | A | T | 0.26 | 0.74 | -0.0377 | 0.007 | 2.52×10-8 |
| rs76145129 | 20 | 62670427 | T | G | 0.12 | 0.12 | -0.0502 | 0.009 | 2.73×10-8 |
| rs190073 | 7 | 10985188 | A | G | 0.41 | 0.41 | -0.0336 | 0.006 | 2.86×10-8 |
| rs17520265 | 7 | 119674508 | A | G | 0.03 | 0.03 | -0.091 | 0.016 | 2.87×10-8 |
| rs647905 | 11 | 121534938 | T | C | 0.46 | 0.54 | 0.0334 | 0.006 | 2.87×10-8 |
| rs11722569 | 4 | 112822731 | T | C | 0.34 | 0.66 | 0.0344 | 0.006 | 2.91×10-8 |
| rs12917449 | 15 | 74331659 | A | C | 0.19 | 0.81 | -0.0419 | 0.008 | 2.97×10-8 |
| rs6597649 | 9 | 133786652 | T | C | 0.4 | 0.4 | 0.0334 | 0.006 | 3.05×10-8 |
| rs9563886 | 13 | 61720066 | T | C | 0.39 | 0.61 | -0.0336 | 0.006 | 3.08×10-8 |
| rs623025 | 1 | 201765094 | T | C | 0.26 | 0.26 | -0.0377 | 0.007 | 3.16×10-8 |
| rs28552587 | 8 | 103356226 | A | G | 0.44 | 0.56 | 0.0334 | 0.006 | 3.30×10-8 |
| rs238869 | 6 | 29355113 | T | C | 0.38 | 0.62 | -0.0336 | 0.006 | 3.36×10-8 |
| rs2737240 | 8 | 116657235 | A | G | 0.29 | 0.71 | 0.0363 | 0.007 | 3.37×10-8 |
| rs138014720 | 3 | 50070843 | A | T | 0.06 | 0.94 | 0.0695 | 0.013 | 3.46×10-8 |
| rs1553754 | 17 | 46563707 | T | G | 0.44 | 0.56 | -0.0336 | 0.006 | 3.51×10-8 |
| rs117630493 | 13 | 54018867 | C | G | 0.03 | 0.97 | -0.1009 | 0.018 | 3.61×10-8 |
| rs73079014 | 3 | 49863483 | T | C | 0.13 | 0.13 | -0.0492 | 0.009 | 3.65×10-8 |
| rs2867690 | 20 | 41972028 | T | C | 0.18 | 0.18 | 0.0421 | 0.008 | 3.70×10-8 |
| rs62383308 | 5 | 165460085 | A | G | 0.08 | 0.08 | -0.0598 | 0.011 | 3.98×10-8 |
| rs4699157 | 4 | 106055212 | T | C | 0.04 | 0.96 | -0.0812 | 0.015 | 3.98×10-8 |
| rs10947987 | 6 | 41754370 | T | C | 0.44 | 0.44 | -0.0325 | 0.006 | 4.08×10-8 |
| rs699844 | 1 | 74878253 | A | G | 0.08 | 0.92 | 0.0602 | 0.011 | 4.11×10-8 |
| rs75932578 | 7 | 106844694 | T | C | 0.22 | 0.22 | -0.0398 | 0.007 | 4.15×10-8 |
| rs6973090 | 7 | 102008352 | A | G | 0.25 | 0.25 | -0.0377 | 0.007 | 4.31×10-8 |
| rs9469434 | 6 | 33455574 | C | G | 0.29 | 0.29 | -0.0356 | 0.007 | 4.41×10-8 |
| rs9964420 | 18 | 56824041 | A | C | 0.3 | 0.3 | 0.0354 | 0.007 | 4.54×10-8 |
| rs6510033 | 19 | 30710785 | A | G | 0.27 | 0.73 | -0.0367 | 0.007 | 4.66×10-8 |
| rs4260410 | 3 | 178469932 | T | C | 0.33 | 0.33 | 0.0344 | 0.006 | 4.87×10-8 |

**Supplement Table IV SNPs analysis of long sleep duration**

| SNP | Chromosome | Position | Effect allele | Other allele | MAF | EAF | Beta | SE | P value |
| --- | --- | --- | --- | --- | --- | --- | --- | --- | --- |
| rs6737318 | 2 | 114083120 | G | A | 0.22 | 0.22 | 0.076 | 0.011 | 3.40×10-13 |
| rs75458655 | 11 | 118115331 | T | C | 0.02 | 0.02 | 0.1848 | 0.0292 | 5.40×10-12 |
| rs17688916 | 17 | 43778680 | T | A | 0.2 | 0.8 | 0.0714 | 0.0125 | 1.10×10-11 |
| rs17817288 | 16 | 53807764 | A | G | 0.48 | 0.52 | 0.0392 | 0.0094 | 8.90×10-9 |
| rs3751046 | 11 | 122828342 | G | A | 0.15 | 0.15 | 0.0695 | 0.0135 | 2.00×10-8 |
| rs7534398 | 1 | 7767464 | A | T | 0.2 | 0.2 | 0.0469 | 0.0118 | 2.10×10-8 |
| rs10899257 | 11 | 76415209 | A | G | 0.14 | 0.14 | 0.0677 | 0.013 | 4.60×10-8 |

**Supplement Table V MR-Egger test for MR analysis of sleep phenotypes and cognitive functions**

| Exposure | Outcome | Egger_intercept | SE | P |
| --- | --- | --- | --- | --- |
| Short sleep duration | Cognitive performance | 0.01024312 | 0.009055157 | 0.2707275 |
| Short sleep duration | Fluid intelligence score | 0.02743893 | 0.01950303 | 0.1740832 |
| Short sleep duration | Memory performance | 0.0102271 | 0.007223354 | 0.1714841 |
| Short sleep duration | TM: Interval in trail 2 path | -0.007268553 | 0.007502336 | 0.3436525 |
| Short sleep duration | TM: Duration to complete trail 2 path | -0.007274059 | 0.007917039 | 0.3686412 |
| Short sleep duration | SDS: Number of correct matches | 0.003271626 | 0.007367698 | 0.6615485 |
| Short sleep duration | SDS: Number of matches attempted | 0.000870268 | 0.007729078 | 0.9114197 |
| Short sleep duration | SDS: Duration to entering value | -0.002925015 | 0.008094208 | 0.7214322 |
| Short sleep duration | PM: Number of incorrect matches | -0.002925015 | 0.008094208 | 0.7214322 |
| Short sleep duration | PM: Time to complete round | -0.001041102 | 0.004293533 | 0.8107597 |
| Short sleep duration | Reaction Time | -0.08825373 | 0.04996933 | 0.1050702 |
| Short sleep duration | Alzheimer's disease | 0.003612144 | 0.02594096 | 0.8905834 |
| Short sleep duration | Lewy body dementia | 0.009242932 | 0.04554824 | 0.8414689 |
| Short sleep duration | Vascular dementia | -0.07748788 | 0.1498815 | 0.6105626 |
| Short sleep duration | Frontotemporal dementia | 0.06453143 | 0.1147512 | 0.630494 |
| Long sleep duration | Cognitive performance | -0.009090332 | 0.005227452 | 0.1570287 |
| Long sleep duration | Fluid intelligence score | -0.02094968 | 0.02043203 | 0.3522269 |
| Long sleep duration | Memory performance | -0.01457701 | 0.008816622 | 0.1591658 |
| Long sleep duration | TM: Interval in trail 2 path | 0.00637643 | 0.006043375 | 0.3396669 |
| Long sleep duration | TM: Duration to complete trail 2 path | 0.000522171 | 0.00622804 | 0.9364354 |
| Long sleep duration | SDS: Number of correct matches | -0.002392007 | 0.00584654 | 0.6993786 |
| Long sleep duration | SDS: Number of matches attempted | -0.002471161 | 0.005844896 | 0.6900157 |
| Long sleep duration | SDS: Duration to entering value | 0.003779178 | 0.005829909 | 0.5454184 |
| Long sleep duration | PM: Number of incorrect matches | 0.001827702 | 0.003109658 | 0.5822405 |
| Long sleep duration | PM: Time to complete round | 0.002162176 | 0.003064052 | 0.5119104 |
| Long sleep duration | Reaction Time | -0.09914813 | 0.1458554 | 0.6199257 |
| Long sleep duration | Alzheimer's disease | -0.0189416 | 0.03707112 | 0.631138 |
| Long sleep duration | Lewy body dementia | -0.02984793 | 0.0563113 | 0.6187607 |
| Long sleep duration | Vascular dementia | 0.02878931 | 0.1523181 | 0.8575193 |
| Long sleep duration | Frontotemporal dementia | / | / | / |
| Insomnia | Cognitive performance | -7.91E-05 | 0.002157437 | 0.9707981 |
| Insomnia | Fluid intelligence score | -0.001491233 | 0.005089699 | 0.7699427 |
| Insomnia | Memory performance | -0.001164457 | 0.002290904 | 0.6120079 |
| Insomnia | TM: Interval in trail 2 path | -0.002962327 | 0.002171116 | 0.1745187 |
| Insomnia | TM: Duration to complete trail 2 path | -0.002723932 | 0.002116872 | 0.2001964 |
| Insomnia | SDS: Number of correct matches | 0.00031213 | 0.002092155 | 0.8816078 |
| Insomnia | SDS: Number of matches attempted | 0.000320934 | 0.002075533 | 0.8773274 |
| Insomnia | SDS: Duration to entering value | 0.000320934 | 0.002075533 | 0.8773274 |
| Insomnia | PM: Number of incorrect matches | -0.00019985 | 0.001330552 | 0.8808119 |
| Insomnia | PM: Time to complete round | -0.000520079 | 0.0014648 | 0.7230602 |
| Insomnia | Reaction Time | -0.0108181 | 0.01875351 | 0.5659983 |
| Insomnia | Alzheimer's disease | -0.005203585 | 0.008040451 | 0.5185705 |
| Insomnia | Lewy body dementia | -0.02105787 | 0.01412943 | 0.1385349 |
| Insomnia | Vascular dementia | -0.0235294 | 0.04083379 | 0.5653761 |
| Insomnia | Frontotemporal dementia | 0.001949037 | 0.08673524 | 0.9823065 |

**Supplement Table VI *Cochran’s Q statistics test for MR analysis of sleep phenotypes and cognitive functions***

| **Exposure** | **Outcome** | **Method** | **Q** | **Q_df** | **P-value** |
| --- | --- | --- | --- | --- | --- |
| Short sleep duration | Cognitive performance | Inverse variance weighted | 232.4954 | 22 | 4.44E-37 |
|  |  | MR Egger | 219.1424 | 21 | 5.96E-35 |
|  | Fluid intelligence score | Inverse variance weighted | 157.0474 | 22 | 2.22E-22 |
|  |  | MR Egger | 143.5198 | 21 | 2.97E-20 |
|  | Memory Performance | Inverse variance weighted | 43.13408 | 22 | 4.54E-03 |
|  |  | MR Egger | 39.37542 | 21 | 8.85E-03 |
|  | TM: Interval in trail 2 path | Inverse variance weighted | 68.04264 | 22 | 1.34E-06 |
|  |  | MR Egger | 65.13142 | 21 | 2.07E-06 |
|  | TM: Duration to complete trail 2 path | Inverse variance weighted | 71.21028 | 22 | 4.26E-07 |
|  |  | MR Egger | 68.45836 | 21 | 6.19E-07 |
|  | SDS: Number of correct matches | Inverse variance weighted | 68.05665 | 22 | 1.33E-06 |
|  |  | MR Egger | 67.42357 | 21 | 9.04E-07 |
|  | SDS: Number of matches attempted | Inverse variance weighted | 74.29251 | 22 | 1.37E-07 |
|  |  | MR Egger | 74.24769 | 21 | 7.21E-08 |
|  | SDS: Duration to entering value | Inverse variance weighted | 82.40689 | 22 | 6.48E-09 |
|  |  | MR Egger | 81.89760 | 21 | 3.88E-09 |
|  | PM: Number of incorrect matches | Inverse variance weighted | 82.40689 | 22 | 6.48E-09 |
|  |  | MR Egger | 81.89760 | 21 | 3.88E-09 |
|  | PM: Time to complete round | Inverse variance weighted | 94.55762 | 22 | 5.68E-11 |
|  |  | MR Egger | 94.29361 | 21 | 2.90E-11 |
|  | Reaction Time | Inverse variance weighted | 23.64807 | 12 | 2.27E-02 |
|  |  | MR Egger | 18.42361 | 11 | 7.23E-02 |
|  | Alzheimer's disease | Inverse variance weighted | 19.33706 | 22 | 6.24E-01 |
|  |  | MR Egger | 19.31767 | 21 | 5.65E-01 |
|  | Lewy body dementia | Inverse variance weighted | 25.78455 | 19 | 1.36E-01 |
|  |  | MR Egger | 25.72570 | 18 | 1.06E-01 |
|  | Vascular dementia | Inverse variance weighted | 30.36473 | 22 | 1.10E-01 |
|  |  | MR Egger | 29.98311 | 21 | 9.23E-02 |
|  | Frontotemporal dementia | Inverse variance weighted | 1.781566 | 3 | 6.19E-01 |
|  |  | MR Egger | 1.465318 | 2 | 4.81E-01 |
| Long sleep duration | Cognitive performance | Inverse variance weighted | 12.107263 | 5 | 3.33E-02 |
|  |  | MR Egger | 6.894813 | 4 | 1.42E-01 |
|  | Fluid intelligence score | Inverse variance weighted | 22.55077 | 6 | 9.62E-04 |
|  |  | MR Egger | 18.63296 | 5 | 2.25E-03 |
|  | Memory Performance | Inverse variance weighted | 10.856103 | 6 | 9.29E-02 |
|  |  | MR Egger | 7.018803 | 5 | 2.19E-01 |
|  | TM: Interval in trail 2 path | Inverse variance weighted | 3.309711 | 6 | 7.69E-01 |
|  |  | MR Egger | 2.196453 | 5 | 8.21E-01 |
|  | TM: Duration to complete trail 2 path | Inverse variance weighted | 1.210051 | 6 | 9.76E-01 |
|  |  | MR Egger | 1.203022 | 5 | 9.45E-01 |
|  | SDS: Number of correct matches | Inverse variance weighted | 3.144874 | 6 | 7.90E-01 |
|  |  | MR Egger | 2.977485 | 5 | 7.03E-01 |
|  | SDS: Number of matches attempted | Inverse variance weighted | 3.430508 | 6 | 7.53E-01 |
|  |  | MR Egger | 3.251757 | 5 | 6.61E-01 |
|  | SDS: Duration to entering value | Inverse variance weighted | 2.822994 | 6 | 8.31E-01 |
|  |  | MR Egger | 2.402779 | 5 | 7.91E-01 |
|  | PM: Number of incorrect matches | Inverse variance weighted | 6.404076 | 6 | 3.79E-01 |
|  |  | MR Egger | 5.990212 | 5 | 3.07E-01 |
|  | PM: Time to complete round | Inverse variance weighted | 6.243736 | 6 | 3.96E-01 |
|  |  | MR Egger | 5.678235 | 5 | 3.39E-01 |
|  | Reaction Time | Inverse variance weighted | 5.955684 | 2 | 5.09E-02 |
|  |  | MR Egger | 4.073412 | 1 | 4.36E-02 |
|  | Alzheimer's disease | Inverse variance weighted | 7.470748 | 6 | 2.79E-01 |
|  |  | MR Egger | 7.100023 | 5 | 2.13E-01 |
|  | Lewy body dementia | Inverse variance weighted | 6.518167 | 6 | 3.68E-01 |
|  |  | MR Egger | 6.171390 | 5 | 2.90E-01 |
|  | Vascular dementia | Inverse variance weighted | 1.466067 | 6 | 9.62E-01 |
|  |  | MR Egger | 1.430343 | 5 | 9.21E-01 |
|  | Frontotemporal dementia | Inverse variance weighted | / | / | / |
|  |  | MR Egger | / | / | / |
| Insomnia | Cognitive performance | Inverse variance weighted | 718.563 | 149 | 2.02E-75 |
|  |  | MR Egger | 718.5564 | 148 | 9.16E-76 |
|  | Fluid intelligence score | Inverse variance weighted | 546.0149 | 148 | 5.74E-47 |
|  |  | MR Egger | 545.6962 | 147 | 3.34E-47 |
|  | Memory Performance | Inverse variance weighted | 222.8056 | 148 | 6.85E-05 |
|  |  | MR Egger | 222.4147 | 147 | 5.89E-05 |
|  | TM: Interval in trail 2 path | Inverse variance weighted | 308.5954 | 148 | 2.31E-13 |
|  |  | MR Egger | 304.7361 | 147 | 4.42E-13 |
|  | TM: Duration to complete trail 2 path | Inverse variance weighted | 276.2906 | 148 | 8.28E-10 |
|  |  | MR Egger | 273.2131 | 147 | 1.25E-09 |
|  | SDS: Number of correct matches | Inverse variance weighted | 303.5244 | 148 | 8.81E-13 |
|  |  | MR Egger | 303.4784 | 147 | 6.16E-13 |
|  | SDS: Number of matches attempted | Inverse variance weighted | 298.9236 | 148 | 2.92E-12 |
|  |  | MR Egger | 298.8750 | 147 | 2.06E-12 |
|  | SDS: Duration to entering value | Inverse variance weighted | 298.9236 | 148 | 2.92E-12 |
|  |  | MR Egger | 298.8750 | 147 | 2.06E-12 |
|  | PM: Number of incorrect matches | Inverse variance weighted | 518.0095 | 148 | 1.51E-42 |
|  |  | MR Egger | 517.9300 | 147 | 8.26E-43 |
|  | PM: Time to complete round | Inverse variance weighted | 613.5549 | 148 | 5.94E-58 |
|  |  | MR Egger | 613.0292 | 147 | 3.54E-58 |
|  | Reaction Time | Inverse variance weighted | 66.92737 | 67 | 4.80E-01 |
|  |  | MR Egger | 66.59162 | 66 | 4.56E-01 |
|  | Alzheimer's disease | Inverse variance weighted | 166.1691 | 142 | 8.09E-02 |
|  |  | MR Egger | 165.677 | 141 | 7.63E-02 |
|  | Lewy body dementia | Inverse variance weighted | 142.9648 | 132 | 2.43E-01 |
|  |  | MR Egger | 140.5812 | 131 | 2.68E-01 |
|  | Vascular dementia | Inverse variance weighted | 180.0246 | 143 | 1.95E-02 |
|  |  | MR Egger | 179.6047 | 142 | 1.79E-02 |
|  | Frontotemporal dementia | Inverse variance weighted | 22.22506 | 20 | 3.28E-01 |
|  |  | MR Egger | 22.22447 | 19 | 2.73E-01 |

**Supplement Table VII *The results of MR analyses using the Outlier-corrected method in MR-PRESSO with the results of the original MR analyses***

| **Exposure** | **Outcome** | **MR Analysis** | **Causal Estimate** | **Sd** | **T-stat** | **P-value** | **Global Test$P-value** | **Distortion Test$P-value** |
| --- | --- | --- | --- | --- | --- | --- | --- | --- |
| Long sleep duration | Cognitive performance | Raw | \ | \ | \ | \ | 0.0553 | No significant outliers |
|  |  | Outlier-corrected | \ | \ | \ | \ |  |  |
|  | Fluid intelligence score | Raw | 0.0254 | 0.1099 | 0.2309 | 0.8251 | 0.0023 | 0.0713 |
|  |  | Outlier-corrected | 0.0054 | 0.1036 | 0.0520 | 0.9610 |  |  |
|  | Memory performance | Raw | \ | \ | \ | \ | 0.1260 | No significant outliers |
|  |  | Outlier-corrected | \ | \ | \ | \ |  |  |
|  | TM: Interval in trail 2 path | Raw | \ | \ | \ | \ | 0.8043 | No significant outliers |
|  |  | Outlier-corrected | \ | \ | \ | \ |  |  |
|  | TM: Duration to complete trail 2 path | Raw | \ | \ | \ | \ | 0.9683 | No significant outliers |
|  |  | Outlier-corrected | \ | \ | \ | \ |  |  |
|  | SDS: Number of correct matches | Raw | \ | \ | \ | \ | 0.7870 | No significant outliers |
|  |  | Outlier-corrected | \ | \ | \ | \ |  |  |
|  | SDS: Number of matches attempted | Raw | \ | \ | \ | \ | 0.7380 | No significant outliers |
|  |  | Outlier-corrected | \ | \ | \ | \ |  |  |
|  | SDS: Duration to entering value | Raw | \ | \ | \ | \ | 0.8527 | No significant outliers |
|  |  | Outlier-corrected | \ | \ | \ | \ |  |  |
|  | PM: Number of incorrect matches | Raw | \ | \ | \ | \ | 0.3957 | No significant outliers |
|  |  | Outlier-corrected | \ | \ | \ | \ |  |  |
|  | PM: Time to complete round | Raw | \ | \ | \ | \ | 0.3903 | No significant outliers |
|  |  | Outlier-corrected | \ | \ | \ | \ |  |  |
|  | Reaction Time | Raw | \ | \ | \ | \ | * | \ |
|  |  | Outlier-corrected | \ | \ | \ | \ |  |  |
|  | Alzheimer's disease | Raw | \ | \ | \ | \ | 0.3580 | No significant outliers |
|  |  | Outlier-corrected | \ | \ | \ | \ |  |  |
|  | Lewy body dementia | Raw | \ | \ | \ | \ | 0.3917 | No significant outliers |
|  |  | Outlier-corrected | \ | \ | \ | \ |  |  |
|  | Vascular dementia | Raw | \ | \ | \ | \ | 0.9663 | No significant outliers |
|  |  | Outlier-corrected | \ | \ | \ | \ |  |  |
|  | Frontotemporal dementia | Raw | \ | \ | \ | \ | * | \ |
|  |  | Outlier-corrected | \ | \ | \ | \ |  |  |
| Short sleep duration | Cognitive performance | Raw | -0.1477 | 0.0634 | -2.3290 | 0.0294 | <0.0003 | 0.5807 |
|  |  | Outlier-corrected | -0.1334 | 0.0391 | -3.4116 | 0.0039 |  |  |
|  | Fluid intelligence score | Raw | -0.3775 | 0.1387 | -2.7212 | 0.0125 | <0.0003 | 0.7140 |
|  |  | Outlier-corrected | -0.2202 | 0.0720 | -3.0604 | 0.0075 |  |  |
|  | Memory performance | Raw | \ | \ | \ | \ | 0.0567 | No significant outliers |
|  |  | Outlier-corrected | \ | \ | \ | \ |  |  |
|  | TM: Interval in trail 2 path | Raw | 0.1081 | 0.0521 | 2.0746 | 0.0499 | <0.0003 | 0.7637 |
|  |  | Outlier-corrected | 0.0975 | 0.0458 | 2.1281 | 0.0460 |  |  |
|  | TM: Duration to complete trail 2 path | Raw | 0.1098 | 0.0549 | 2.0004 | 0.0580 | <0.0003 | 0.6897 |
|  |  | Outlier-corrected | 0.0948 | 0.0488 | 1.9420 | 0.0664 |  |  |
|  | SDS: Number of correct matches | Raw | \ | \ | \ | \ | 0.6870 | No significant outliers |
|  |  | Outlier-corrected | \ | \ | \ | \ |  |  |
|  | SDS: Number of matches attempted | Raw | \ | \ | \ | \ | 0.8112 | No significant outliers |
|  |  | Outlier-corrected | \ | \ | \ | \ |  |  |
|  | SDS: Duration to entering value | Raw | \ | \ | \ | \ | 0.8996 | No significant outliers |
|  |  | Outlier-corrected | \ | \ | \ | \ |  |  |
|  | PM: Number of incorrect matches | Raw | 0.0202 | 0.0238 | 0.8487 | 0.4052 | <0.0003 | 0.2447 |
|  |  | Outlier-corrected | 0.0094 | 0.0198 | 0.4779 | 0.6382 |  |  |
|  | PM: Time to complete round | Raw | -0.0003 | 0.0293 | -0.0105 | 0.2925 | <0.0003 | 0.1197 |
|  |  | Outlier-corrected | 0.0069 | 0.0236 | 0.2925 | 0.7729 |  |  |
|  | Reaction Time | Raw | 0.0456 | 0.3629 | 0.1256 | 0.9021 | 0.0233 | 0.2480 |
|  |  | Outlier-corrected | -0.1915 | 0.3236 | -0.5918 | 0.5660 |  |  |
|  | Alzheimer's disease | Raw | \ | \ | \ | \ | 0.6373 | No significant outliers |
|  |  | Outlier-corrected | \ | \ | \ | \ |  |  |
|  | Lewy body dementia | Raw | \ | \ | \ | \ | 0.1490 | No significant outliers |
|  |  | Outlier-corrected | \ | \ | \ | \ |  |  |
|  | Vascular dementia | Raw | \ | \ | \ | \ | 0.1170 | No significant outliers |
|  |  | Outlier-corrected | \ | \ | \ | \ |  |  |
|  | Frontotemporal dementia | Raw | \ | \ | \ | \ | 0.6677 | No significant outliers |
|  |  | Outlier-corrected | \ | \ | \ | \ |  |  |
| Insomnia | Cognitive performance | Raw | -0.0170 | 0.0130 | -1.3067 | 0.1933 | <0.0003 | 0.1817 |
|  |  | Outlier-corrected | -0.0106 | 0.0092 | -1.1474 | 0.2533 |  |  |
|  | Fluid intelligence score | Raw | -0.0457 | 0.0305 | -1.4982 | 0.1362 | <0.0003 | 0.9357 |
|  |  | Outlier-corrected | -0.0439 | 0.0249 | -1.7625 | 0.0802 |  |  |
|  | Memory performance | Raw | -0.0024 | 0.0138 | -0.1723 | 0.8634 | 0.0003 | 0.1360 |
|  |  | Outlier-corrected | 0.0038 | 0.0130 | 0.2925 | 0.7703 |  |  |
|  | TM: Interval in trail 2 path | Raw | 0.0094 | 0.0131 | 0.7194 | 0.4730 | <0.0003 | 0.2517 |
|  |  | Outlier-corrected | 0.0032 | 0.0121 | 0.2658 | 0.7908 |  |  |
|  | TM: Duration to complete trail 2 path | Raw | 0.0055 | 0.0127 | 0.4317 | 0.6666 | <0.0003 | 0.1280 |
|  |  | Outlier-corrected | -0.0033 | 0.0119 | -0.2741 | 0.7844 |  |  |
|  | SDS: Number of correct matches | Raw | -0.0118 | 0.0125 | -0.9449 | 0.3462 | <0.0003 | 0.6300 |
|  |  | Outlier-corrected | -0.0084 | 0.0117 | -0.7198 | 0.4728 |  |  |
|  | SDS: Number of matches attempted | Raw | -0.0116 | 0.0124 | -0.9355 | 0.3510 | <0.0003 | 0.9483 |
|  |  | Outlier-corrected | -0.0109 | 0.0114 | -0.9565 | 0.3404 |  |  |
|  | SDS: Duration to entering value | Raw | 0.0068 | 0.0123 | 0.5589 | 0.5771 | <0.0003 | 0.2017 |
|  |  | Outlier-corrected | 0.0022 | 0.0113 | 0.1918 | 0.8482 |  |  |
|  | PM: Number of incorrect matches | Raw | 0.0046 | 0.0080 | 0.5725 | 0.5679 | <0.0003 | 0.9607 |
|  |  | Outlier-corrected | 0.0049 | 0.0064 | 0.7693 | 0.4430 |  |  |
|  | PM: Time to complete round | Raw | 0.0046 | 0.0080 | 0.5725 | 0.5679 | <0.0003 | 0.8397 |
|  |  | Outlier-corrected | 0.0062 | 0.0064 | 0.9687 | 0.3344 |  |  |
|  | Reaction Time | Raw | \ | \ | \ | \ | 0.4750 | No significant outliers |
|  |  | Outlier-corrected | \ | \ | \ | \ |  |  |
|  | Alzheimer's disease | Raw | \ | \ | \ | \ | 0.0870 | No significant outliers |
|  |  | Outlier-corrected | \ | \ | \ | \ |  |  |
|  | Lewy body dementia | Raw | \ | \ | \ | \ | 0.2320 | No significant outliers |
|  |  | Outlier-corrected | \ | \ | \ | \ |  |  |
|  | Vascular dementia | Raw | 0.1218 | 0.2495 | 0.4880 | 0.6263 | 0.0177 | 0.9377 |
|  |  | Outlier-corrected | 0.1423 | 0.2233 | 0.6373 | 0.5250 |  |  |
|  | Frontotemporal dementia | Raw | \ | \ | \ | \ | 0.3670 | No significant outliers |
|  |  | Outlier-corrected | \ | \ | \ | \ |  |  |

**Supplement Table VIII *The no. of SNPs determined in this MR study***

The criteria for SNP selection may include but are not limited to:

1. Genome-wide association studies (GWAS) reporting significant associations between the SNPs and the exposure variable. We tried to find new, large sample-sized sources of GWAS.

2. SNPs with established functionality or biological relevance related to the exposure，p<5e-8.

3. Consideration of linkage disequilibrium patterns to avoid redundancy and ensure independence of SNPs in the analysis.

4. Sensitivity analyses to evaluate the robustness of results to different SNP inclusion criteria.

Through this comprehensive approach, this study aimed to maximize the validity and strength of Mendelian randomization analysis, ensuring that the selected SNPs provide reliable instrumental variables for estimating causal effects.

For short sleep duration:

Firstly, we selected the GWAS data of Dashti et al (2019) with a large sample size, and 26 SNPs exceeding the genome-wide statistical significance threshold (p < 5×10-8) were identified as instrumental variables for short duration sleep. Afterwards, to avoid bias, the remaining SNPs (25 SNPs for cognitive performance, 25 SNPs for fluid intelligence score, 25 SNPs for memory performance, 25 SNPs for TM: Interval in trail 2 path, 25 SNPs for TM: duration to complete trail 2 path, 25 SNPs for SDS: number of correct matches, 25 SNPs for SDS: number of matches attempted, 25 SNPs for SDS: duration to entering value, 25 SNPs for PM: number of incorrect matches, 25 SNPs for PM: time to complete round, 15 SNPs for reaction time, 25 SNPs for Alzheimer's Disease, 22 SNPs for Lewy body dementia, 25 SNPs for Vascular dementia, 4 SNPs for Frontotemporal dementia) were analysed by eliminating linkage disequilibrium (threshold r2 < 0.001 and a genetic distance of 10,000 kb) AND extracting data from the Sleep Phenotype GWAS database and the Cognitive Function GWAS database, and collating and merging them. We removed palindromic SNPs(rs2186122, rs9367621 be removed for cognitive performance; rs2186122, rs9367621 be removed for fluid intelligence score; rs2186122, rs9367621 be removed for memory performance; rs2186122, rs9367621 be removed for TM: Interval in trail 2 path; rs2186122, rs9367621 be removed for TM: duration to complete trail 2 path; rs2186122, rs9367621 be removed for SDS: number of correct matches; rs2186122, rs9367621 be removed for SDS: number of matches attempted; rs2186122, rs9367621 be removed for SDS: duration to entering value; rs2186122, rs9367621 be removed for PM: number of incorrect matches; rs2186122, rs9367621 be removed for PM: time to complete round; rs2186122, rs9367621 be removed for reaction time; rs2186122, rs9367621 be removed for Alzheimer's Disease; rs2186122, rs9367621 be removed for Lewy body dementia; rs2186122, rs9367621 be removed for Vascular dementia; 0 SNP be removed for Frontotemporal dementia) to ensure that the instrumental variables in the exposure and outcome datasets were consistent and all derived from the same orientation of the DNA strand. In addition, to investigate whether single nucleotide polymorphisms associated with sleep representations were significantly associated with other confounders at the genome-wide level, these single nucleotide polymorphisms associated with sleep representations were searched in the PhenoScanner database (http://www.phenoscanner.medschl.cam.ac.uk/). Potential confounders associated with influencing cognitive outcomes were excluded. Therefore, we determined the number of SNPs used for MR analysis (23 SNPs for cognitive performance, 23 SNPs for fluid intelligence score, 23 SNPs for memory performance, 23 SNPs for TM: Interval in trail 2 path, 23 SNPs for TM: duration to complete trail 2 path, 23 SNPs for SDS: number of correct matches, 23 SNPs for SDS: number of matches attempted, 23 SNPs for SDS: duration to entering value, 23 SNPs for PM: number of incorrect matches, 23 SNPs for PM: time to complete round, 13 SNPs for reaction time, 23 SNPs for Alzheimer's Disease, 20 SNPs for Lewy body dementia, 23 SNPs for Vascular dementia, 4 SNPs for Frontotemporal dementia). In the sensitivity analysis, MR-PRESSO test and leave-one-out analyses were conducted to evaluate the robustness of results to different SNP inclusion criteria.

For insomnia:

Firstly, we selected the GWAS data of Jansen et al (2019) with a large sample size, and 240 SNPs exceeding the genome-wide statistical significance threshold (p < 5×10-8) were identified as instrumental variables for short duration sleep. Afterwards, to avoid bias, the remaining SNPs (156 SNPs for cognitive performance, 156 SNPs for fluid intelligence score, 156 SNPs for memory performance, 156 SNPs for TM: Interval in trail 2 path, 156 SNPs for TM: duration to complete trail 2 path, 156 SNPs for SDS: number of correct matches, 156 SNPs for SDS: number of matches attempted, 156 SNPs for SDS: duration to entering value, 156 SNPs for PM: number of incorrect matches, 156 SNPs for PM: time to complete round, 72 SNPs for reaction time, 152 SNPs for Alzheimer's Disease, 140 SNPs for Lewy body dementia, 153 SNPs for Vascular dementia, 21 SNPs for Frontotemporal dementia) were analysed by eliminating linkage disequilibrium (threshold r2 < 0.001 and a genetic distance of 10,000 kb) AND extracting data from the Sleep Phenotype GWAS database and the Cognitive Function GWAS database, and collating and merging them. We removed palindromic SNPs(rs1731951, rs2030672, rs2221119, rs4858708, rs7044885, rs8180817 be removed for cognitive performance; rs12991815, rs1731951, rs2030672, rs2221119, rs4858708, rs7044885, rs8180817 be removed for fluid intelligence score; rs12991815, rs1731951, rs2030672, rs2221119, rs4858708, rs7044885, rs8180817 be removed for memory performance; rs12991815, rs1731951, rs2030672, rs2221119, rs4858708, rs7044885, rs8180817 be removed for TM: Interval in trail 2 path; rs12991815, rs1731951, rs2030672, rs2221119, rs4858708, rs7044885, rs8180817 be removed for TM: duration to complete trail 2 path; rs12991815, rs1731951, rs2030672, rs2221119, rs4858708, rs7044885, rs8180817 be removed for SDS: number of correct matches; rs12991815, rs1731951, rs2030672, rs2221119, rs4858708, rs7044885, rs8180817 be removed for SDS: number of matches attempted; rs12991815, rs1731951, rs2030672, rs2221119, rs4858708, rs7044885, rs8180817 be removed for SDS: duration to entering value; rs12991815, rs1731951, rs2030672, rs2221119, rs4858708, rs7044885, rs8180817 be removed for PM: number of incorrect matches; rs12991815, rs1731951, rs2030672, rs2221119, rs4858708, rs7044885, rs8180817 be removed for PM: time to complete round; rs12991815, rs1731951, rs2030672, rs6119267 be removed for reaction time; rs12991815, rs1731951, rs2030672, rs2221119, rs4858708, rs6119267, rs7044885, rs8180817, rs830716 be removed for Alzheimer's Disease; rs12991815, rs1731951, rs2030672, rs2221119, rs4858708, rs7044885, rs8180817 be removed for Lewy body dementia; rs12991815, rs1731951, rs2030672, rs2221119, rs4858708, rs6119267, rs7044885, rs8180817, rs830716 be removed for Vascular dementia; 0 SNP be removed for Frontotemporal dementia) to ensure that the instrumental variables in the exposure and outcome datasets were consistent and all derived from the same orientation of the DNA strand. In addition, to investigate whether single nucleotide polymorphisms associated with sleep representations were significantly associated with other confounders at the genome-wide level, these single nucleotide polymorphisms associated with sleep representations were searched in the PhenoScanner database (http://www.phenoscanner.medschl.cam.ac.uk/). Potential confounders associated with influencing cognitive outcomes were excluded. Therefore, we determined the number of SNPs used for MR analysis (150 SNPs for cognitive performance, 149 SNPs for fluid intelligence score, 149 SNPs for memory performance, 149 SNPs for TM: Interval in trail 2 path, 149 SNPs for TM: duration to complete trail 2 path, 149 SNPs for SDS: number of correct matches, 149 SNPs for SDS: number of matches attempted, 149 SNPs for SDS: duration to entering value, 149 SNPs for PM: number of incorrect matches, 149 SNPs for PM: time to complete round, 68 SNPs for reaction time, 143 SNPs for Alzheimer's Disease, 133 SNPs for Lewy body dementia, 144 SNPs for Vascular dementia, 21 SNPs for Frontotemporal dementia). In the sensitivity analysis, MR-PRESSO test and leave-one-out analyses were conducted to evaluate the robustness of results to different SNP inclusion criteria.

For long sleep duration:

Firstly, we selected the GWAS data of Dashti et al (2019) with a large sample size, and SNPs exceeding the genome-wide statistical significance threshold (p < 5×10-8) were identified as instrumental variables for short sleep duration. Afterward, to avoid bias, the remaining SNPs (6 SNPs for cognitive performance, 7 SNPs for fluid intelligence score, 7 SNPs for memory performance, 7 SNPs for TM: Interval in trail 2 path, 7 SNPs for TM: duration to complete trail 2 path, 7 SNPs for SDS: number of correct matches, 7 SNPs for SDS: number of matches attempted, 7 SNPs for SDS: duration to entering value, 7 SNPs for PM: number of incorrect matches, 7 SNPs for PM: time to complete round, 3 SNPs for reaction time, 7 SNPs for Alzheimer's Disease, 7 SNPs for Lewy body dementia, 7 SNPs for Vascular dementia, 1 SNPs for Frontotemporal dementia) were analysed by eliminating linkage disequilibrium (threshold r2 < 0.001 and a genetic distance of 10,000 kb) AND extracting data from the Sleep Phenotype GWAS database and the Cognitive Function GWAS database, and collating and merging them. We removed palindromic SNPs(0 SNP be removed for cognitive performance; 0 SNP be removed for fluid intelligence score; 0 SNP be removed for memory performance; 0 SNP be removed for TM: Interval in trail 2 path; 0 SNP be removed for TM: duration to complete trail 2 path; 0 SNP be removed for SDS: number of correct matches; 0 SNP be removed for SDS: number of matches attempted; 0 SNP be removed for SDS: duration to entering value; 0 SNP be removed for PM: number of incorrect matches; 0 SNP be removed for PM: time to complete round; 0 SNP be removed for reaction time; 0 SNP be removed for Alzheimer's Disease; 0 SNP be removed for Lewy body dementia; 0 SNP be removed for Vascular dementia; 0 SNP be removed for Frontotemporal dementia) to ensure that the instrumental variables in the exposure and outcome datasets were consistent and all derived from the same orientation of the DNA strand. In addition, to investigate whether single nucleotide polymorphisms associated with sleep representations were significantly associated with other confounders at the genome-wide level, these single nucleotide polymorphisms associated with sleep representations were searched in the PhenoScanner database (http://www.phenoscanner.medschl.cam.ac.uk/). Potential confounders associated with influencing cognitive outcomes were excluded. Therefore, we determined the number of SNPs used for MR analysis (6 SNPs for cognitive performance, 7 SNPs for fluid intelligence score, 7 SNPs for memory performance, 7 SNPs for TM: Interval in trail 2 path, 7 SNPs for TM: duration to complete trail 2 path, 7 SNPs for SDS: number of correct matches, 7 SNPs for SDS: number of matches attempted, 7 SNPs for SDS: duration to entering value, 7 SNPs for PM: number of incorrect matches, 7 SNPs for PM: time to complete round, 3 SNPs for reaction time, 7 SNPs for Alzheimer's Disease, 7 SNPs for Lewy body dementia, 7 SNPs for Vascular dementia, 1 SNPs for Frontotemporal dementia). In the sensitivity analysis, MR-PRESSO test and leave-one-out analyses were conducted to evaluate the robustness of results to different SNP inclusion criteria.
