# Supplementary material for: Mendelian randomization study of self-reported long sleep duration, short sleep duration, and insomnia and cognitive function
Source: PLoS One. 2025 Aug 20;20(8):e0330782. doi: 10.1371/journal.pone.0330782 (PMC12367179; doi:10.1371/journal.pone.0330782)
Supplement: S1 Fig — Funnel plots for MR analysis of sleep phenotypes and cognitive functions. Leave-one-out analysis for MR analysis of sleep phenotypes and cognitive functions. (DOCX) [file pone.0330782.s002.docx]

**S1 Fig**

**Supplement Fig I *Scatterplot of Mendelian randomisation analysis***

**Exposure: Short sleep duration**

Outcome: Cognitve Performance

Fluid intelligence score (FIS)

Memory Performance


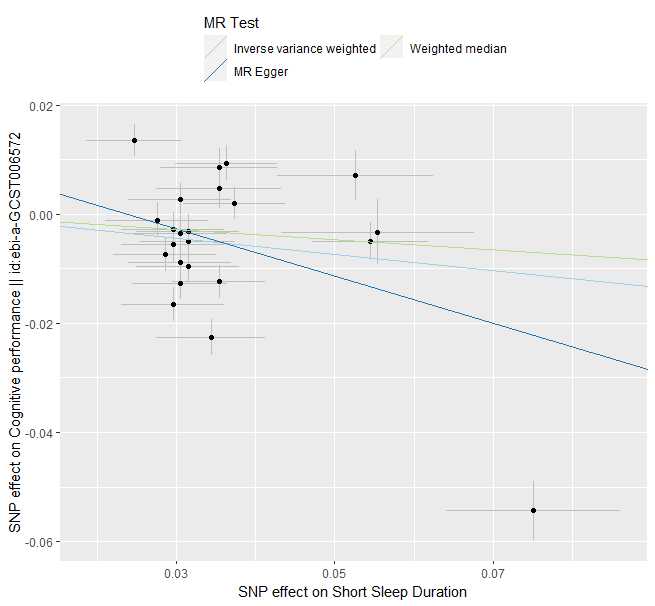

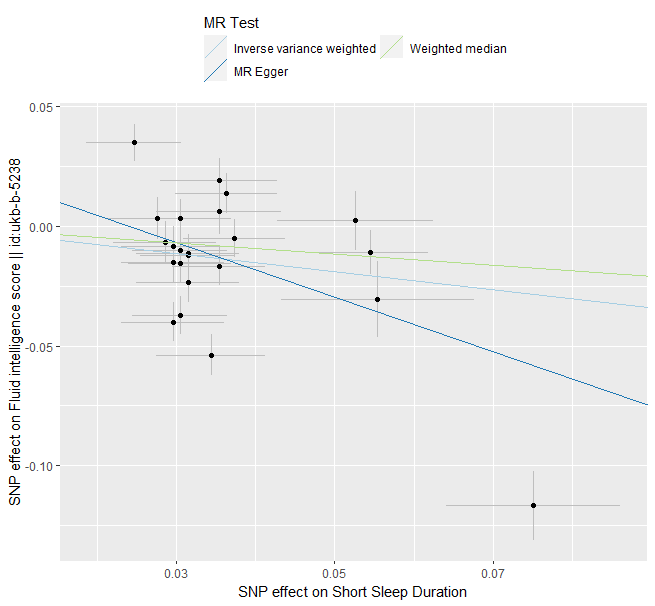

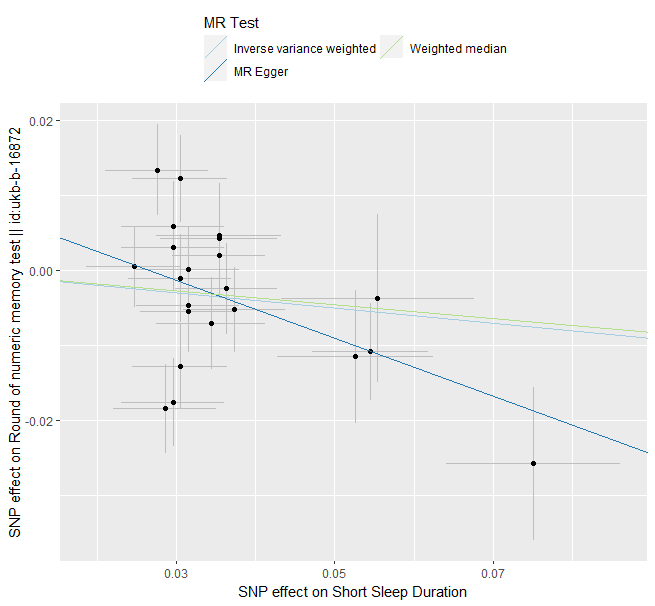


Outcome: TM: Interval in trail 2 path

TM: Duration to complete trail 2 path


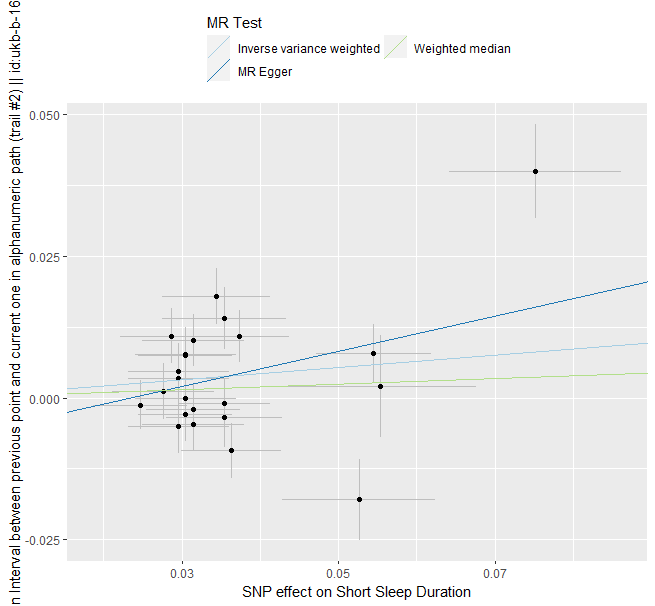

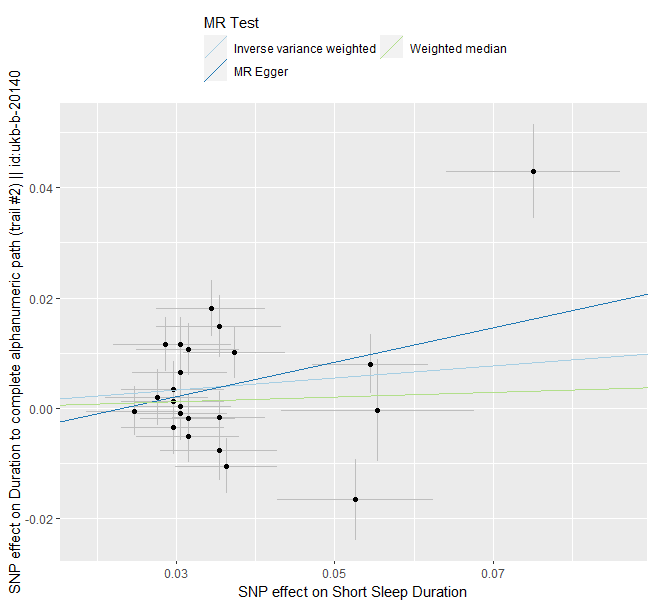


Outcome: SDS: Number of correct matches

SDS: Number of matches attempted

SDS: Duration to entering value


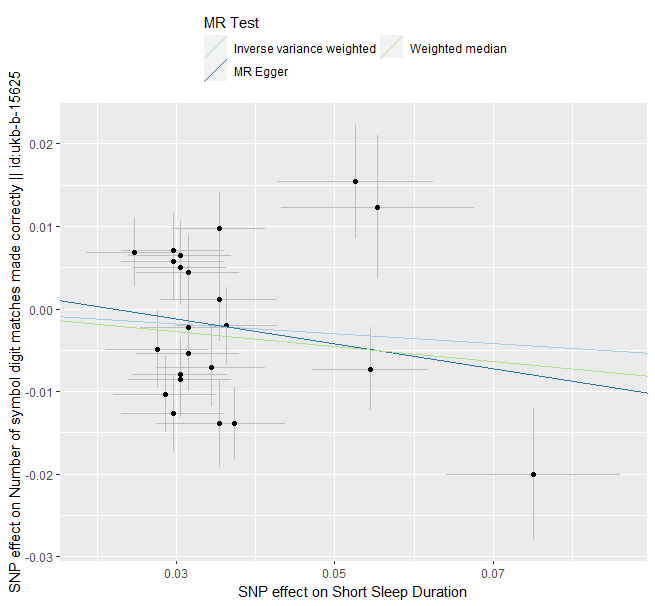

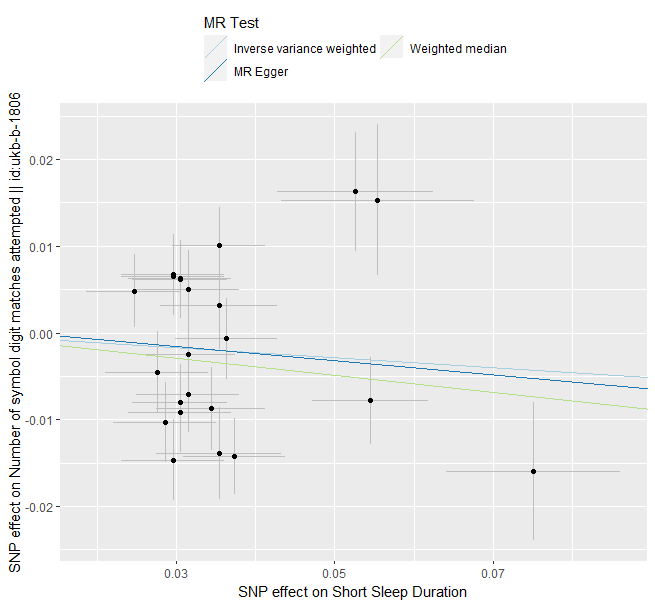

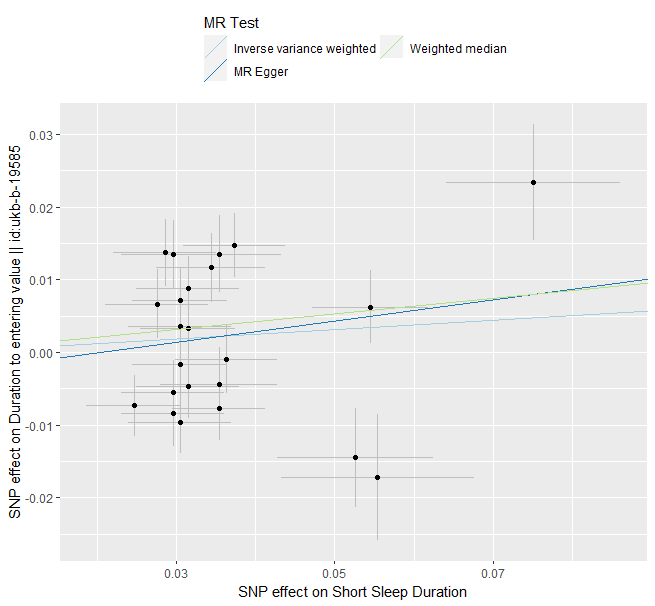


Outcome: PM: Number of incorrect matches

PM: Time to complete round


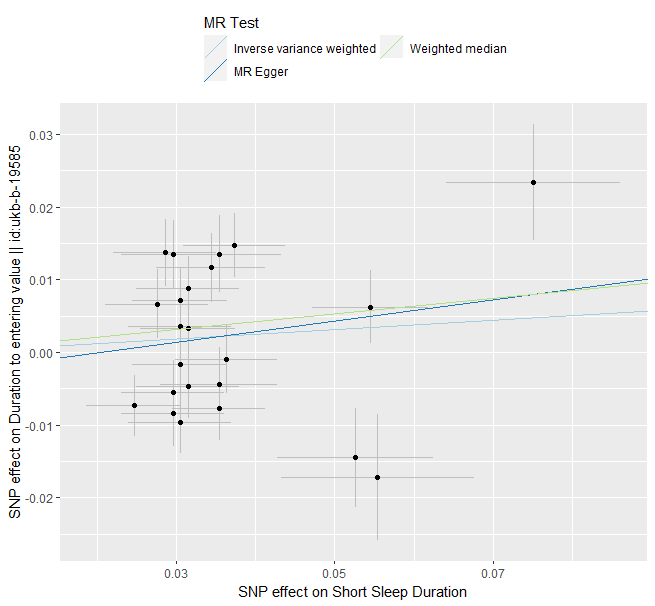

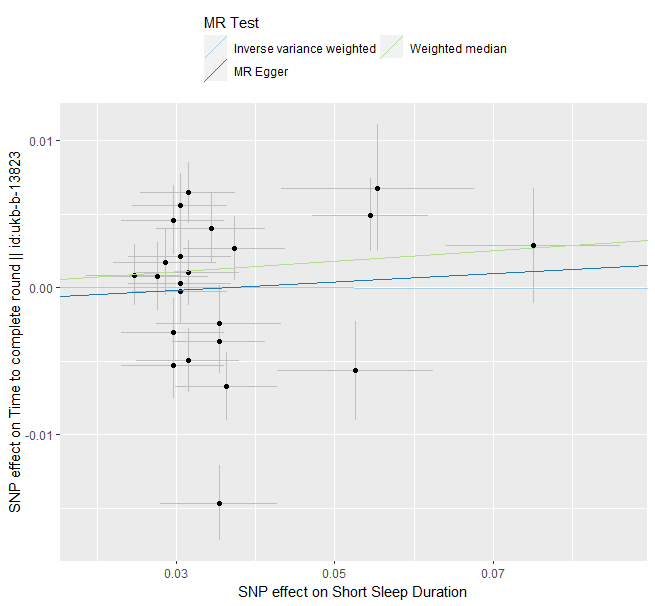


Outcome：Reaction Time


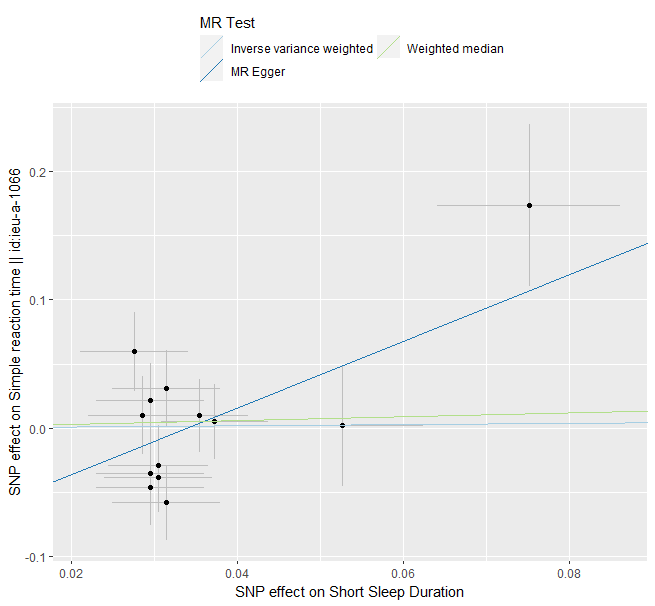


Outcome: Alzheimer's Disease

Lewy Body Dementia (LBD)

Vascular dementia

Frontotemporal dementia


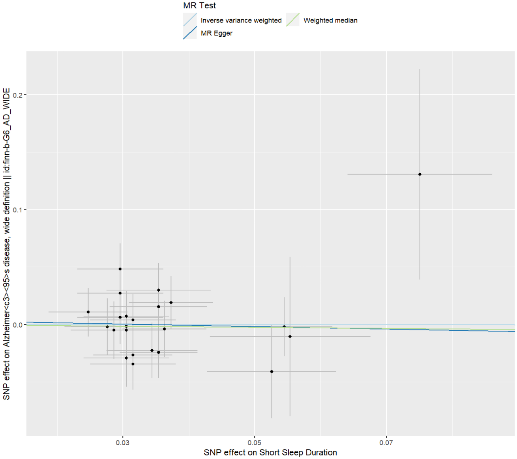

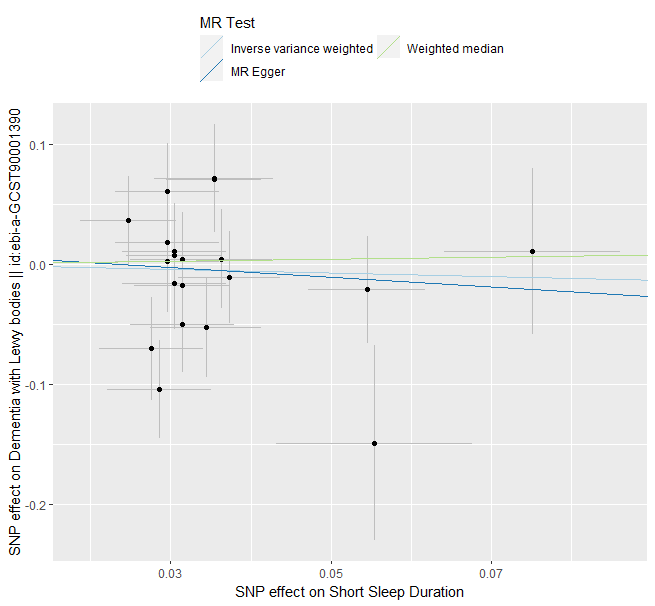


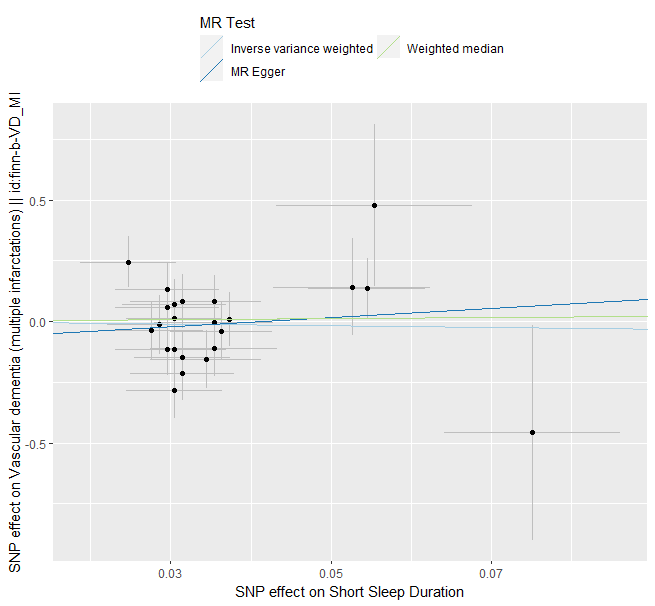

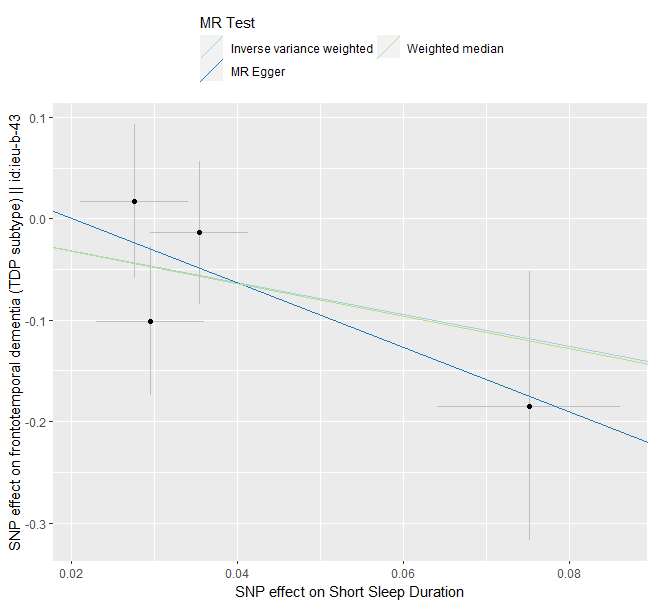


**Exposure: Insomnia**

Outcome: Cognitve Performance

Fluid intelligence score (FIS)

Memory Performance


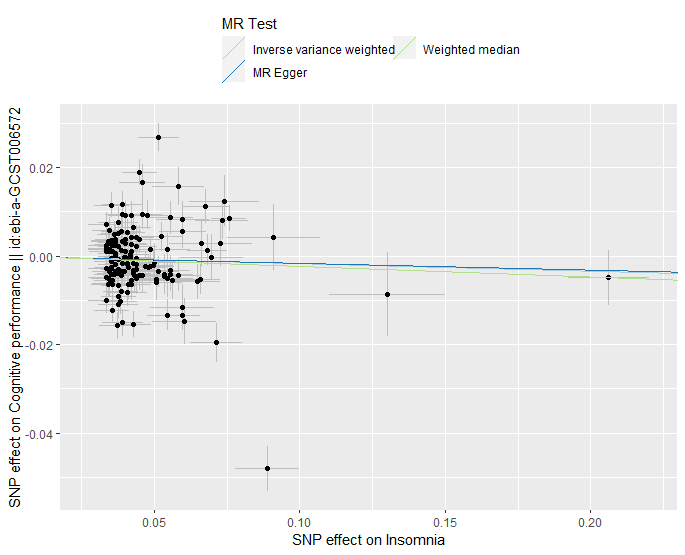

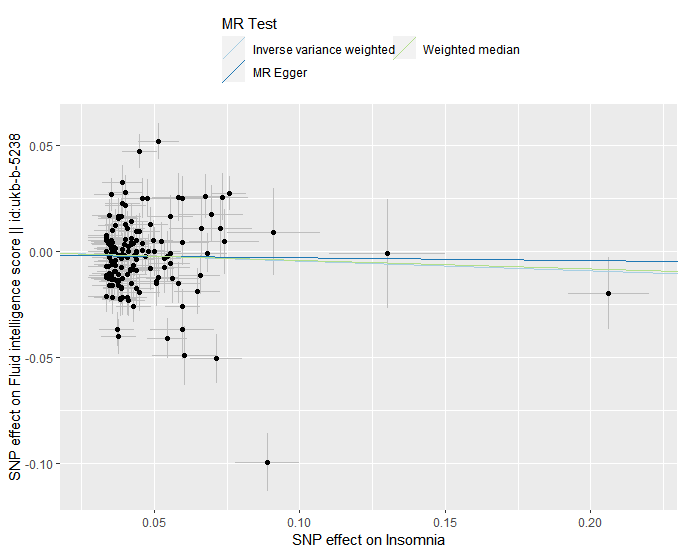

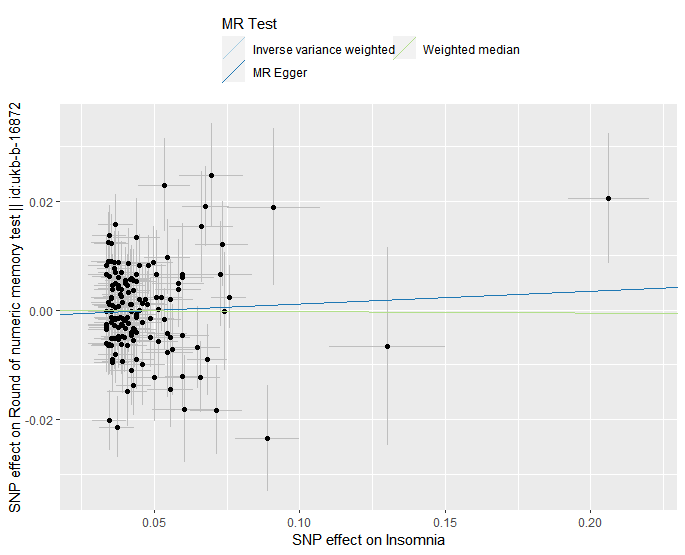


Outcome: TM: Interval in trail 2 path

TM: Duration to complete trail 2 path


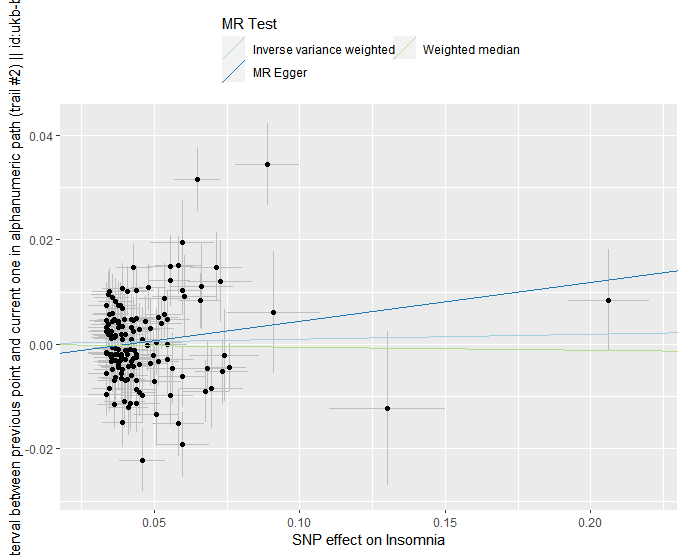

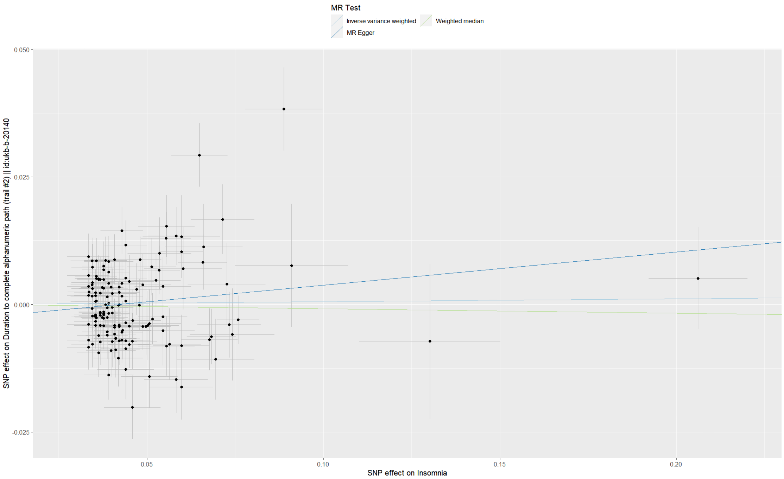


Outcome: SDS: Number of correct matches

SDS: Number of matches attempted

SDS: Duration to entering value


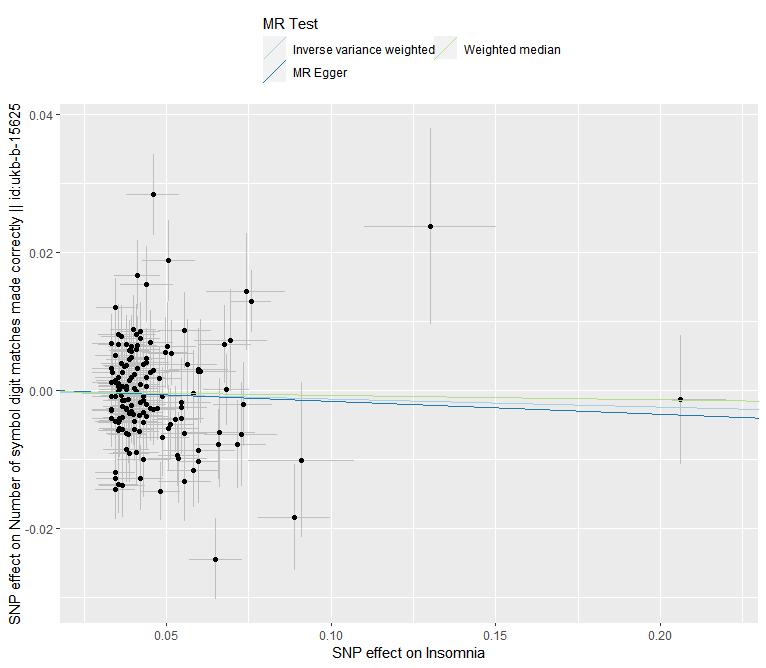

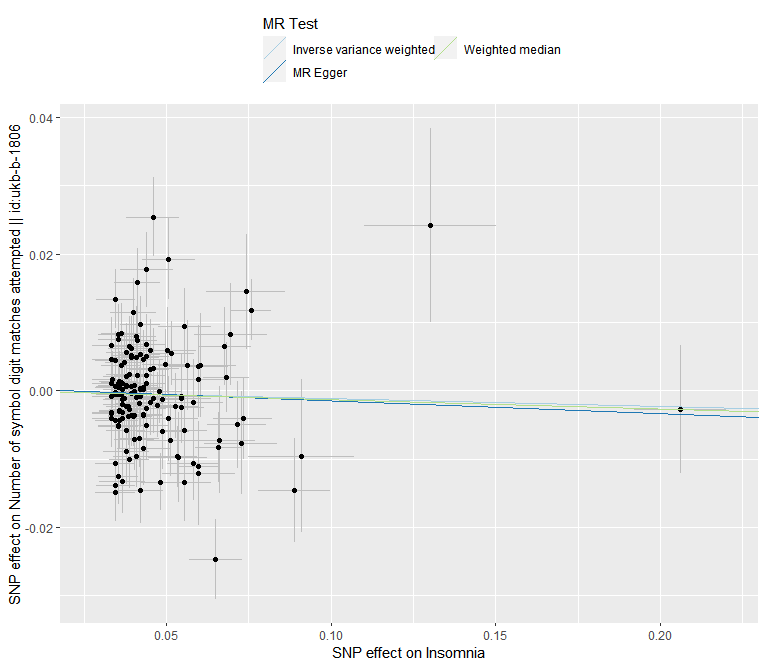

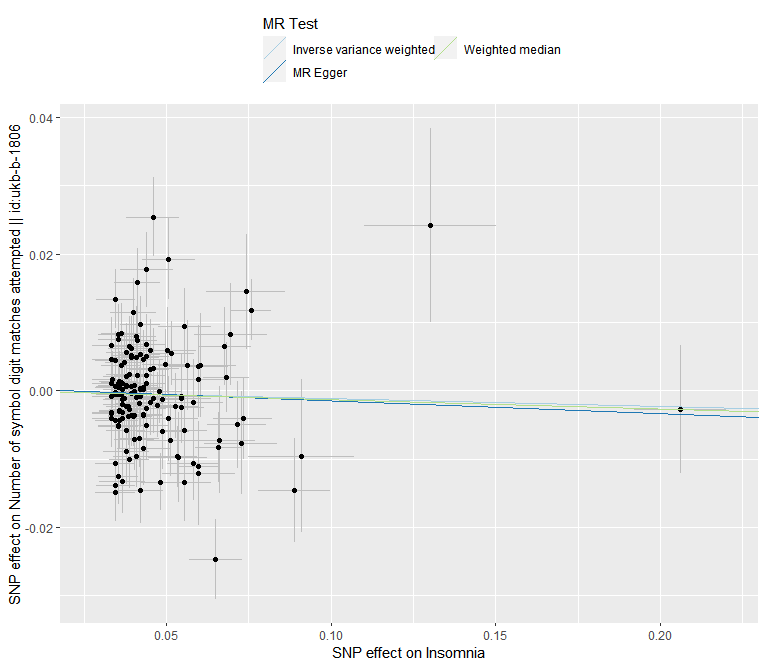


Outcome: PM: Number of incorrect matches

PM: Time to complete round


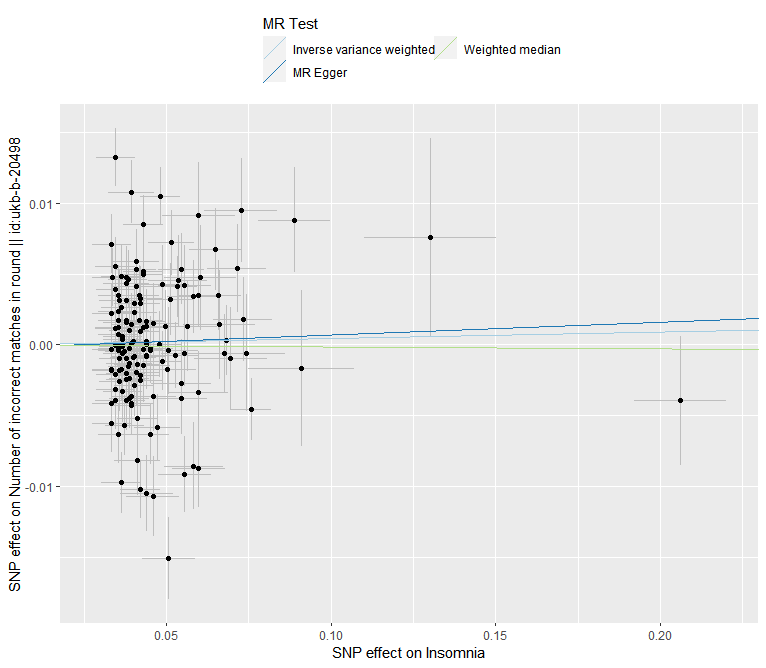

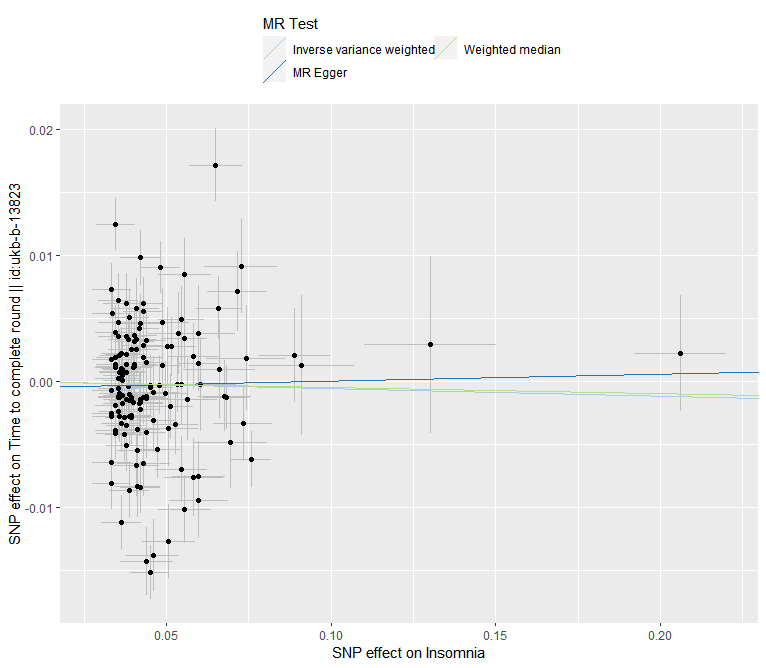


Outcome：Reaction Time


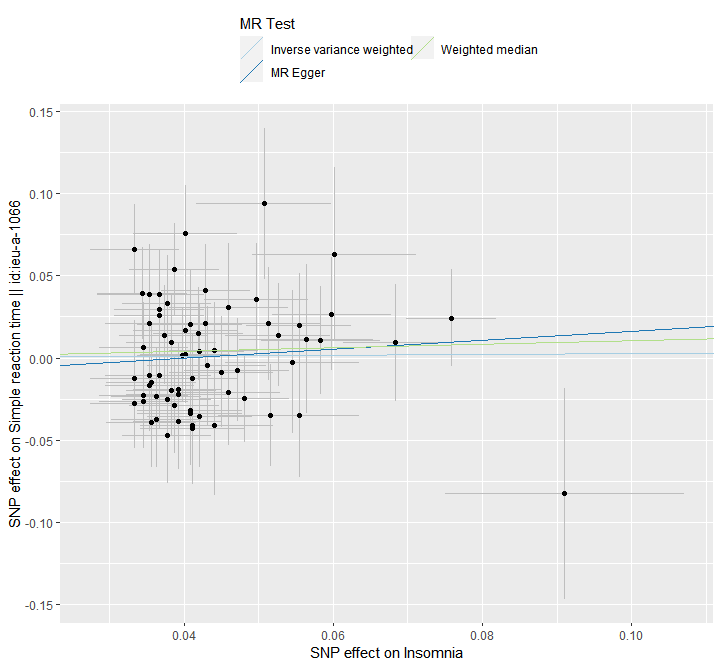


Outcome: Alzheimer's Disease

Lewy Body Dementia (LBD)

Vascular dementia

Frontotemporal dementia


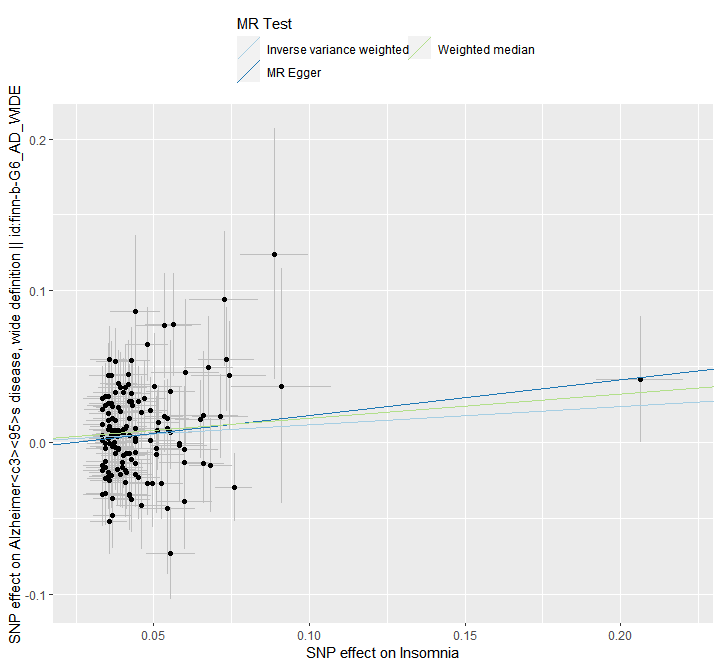

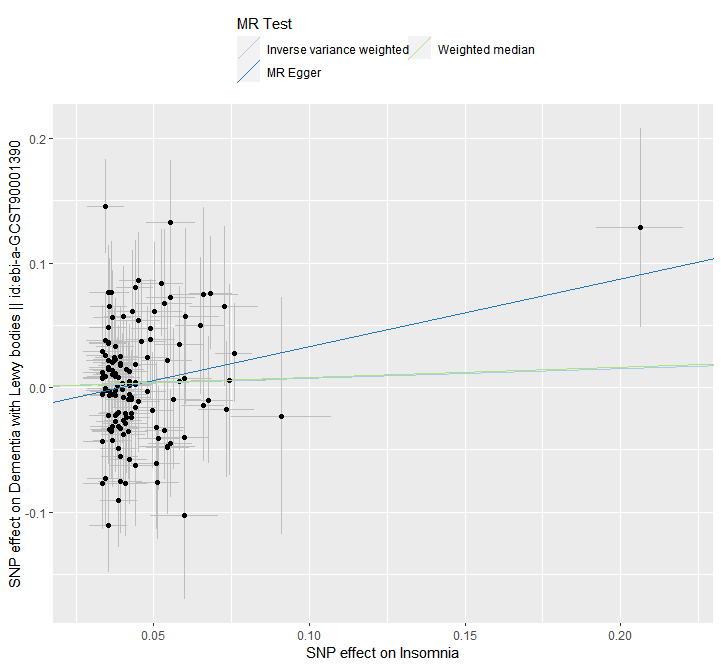


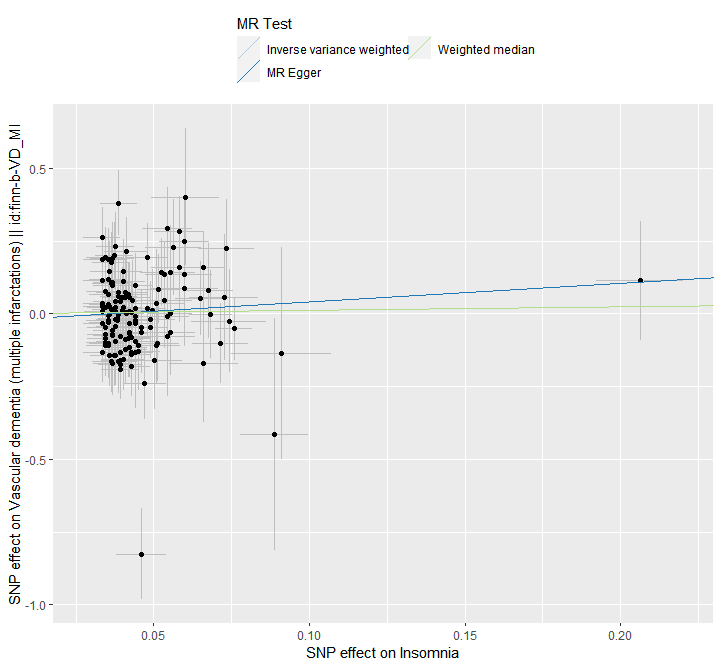

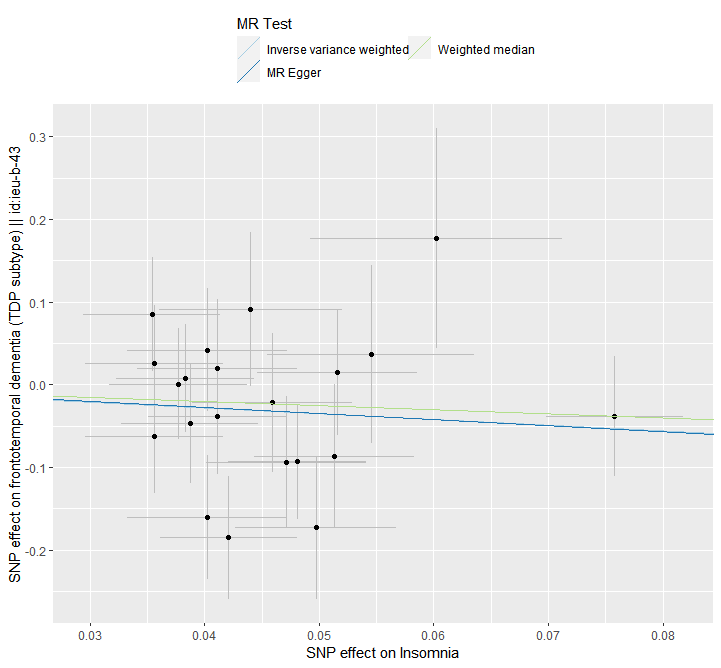


**Exposure: Long sleep duration**

Outcome: Cognitve Performance

Fluid intelligence score (FIS)

Memory Performance


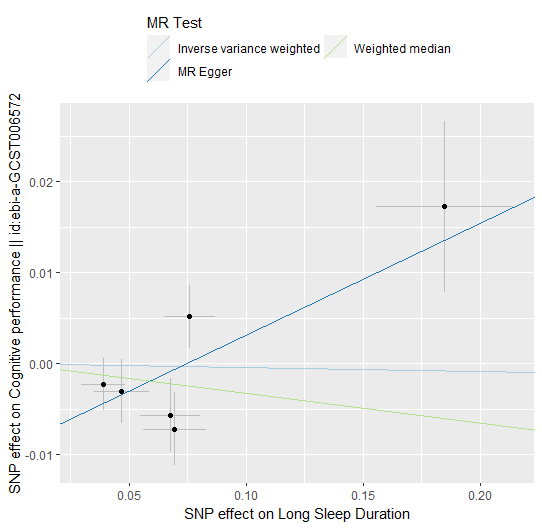

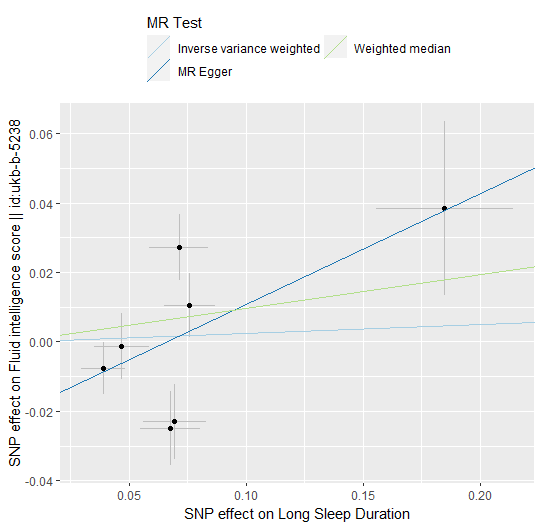

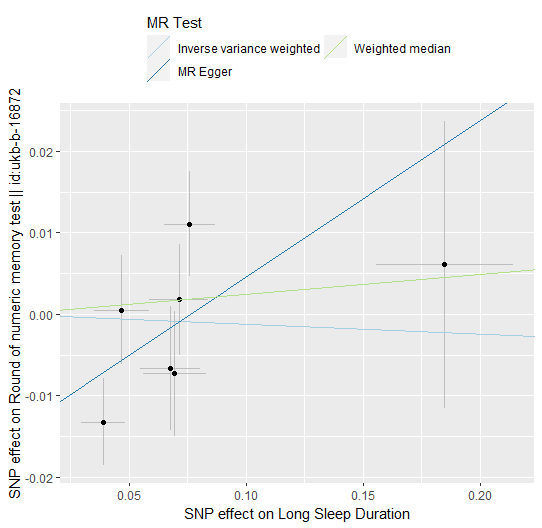


Outcome: TM: Interval in trail 2 path

TM: Duration to complete trail 2 path


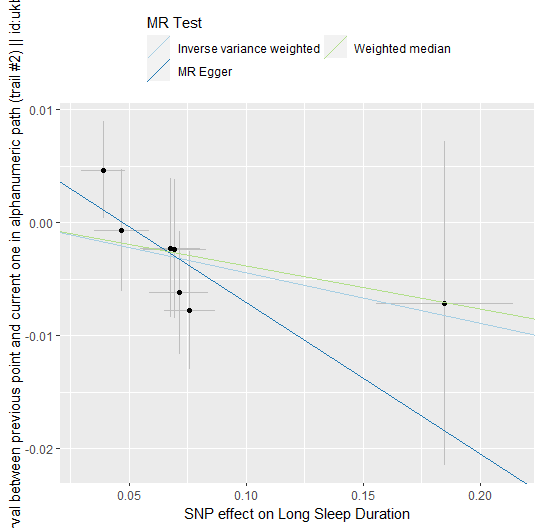

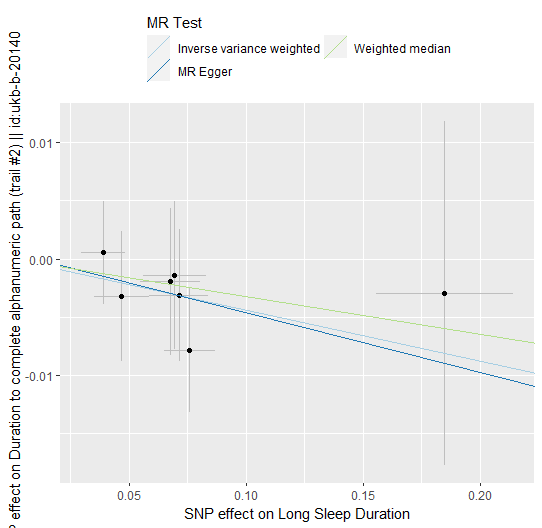


Outcome: SDS: Number of correct matches

SDS: Number of matches attempted

SDS: Duration to entering value


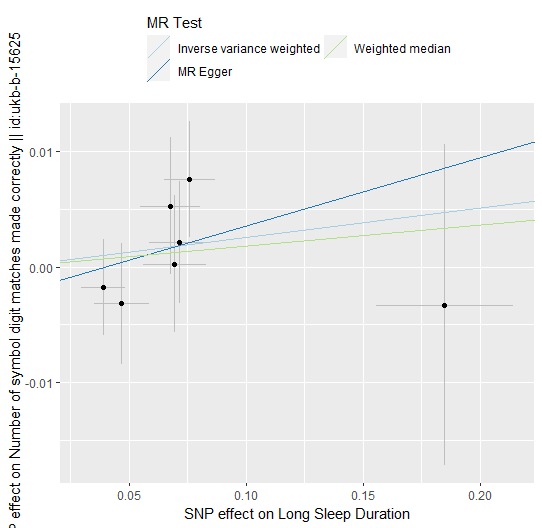

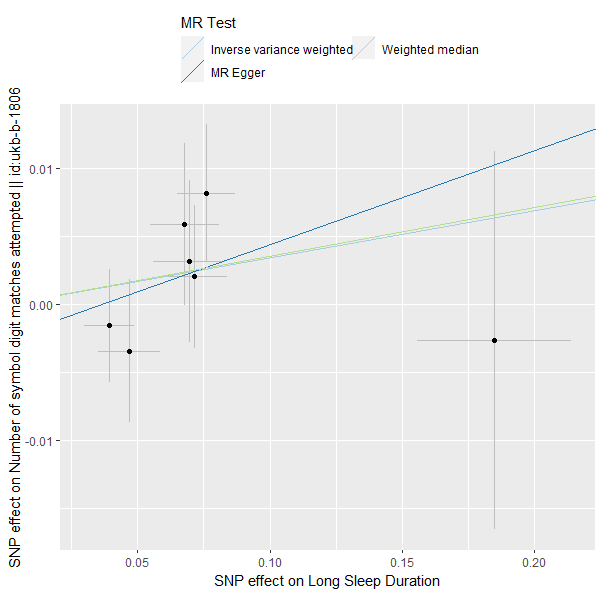

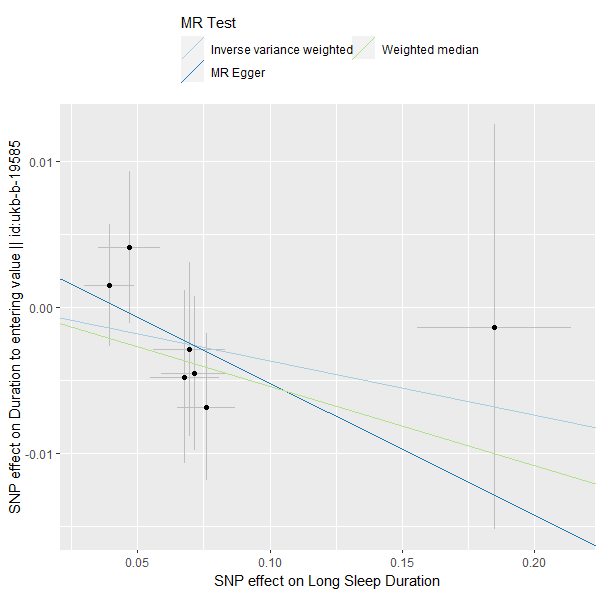


Outcome: PM: Number of incorrect matches

PM: Time to complete round


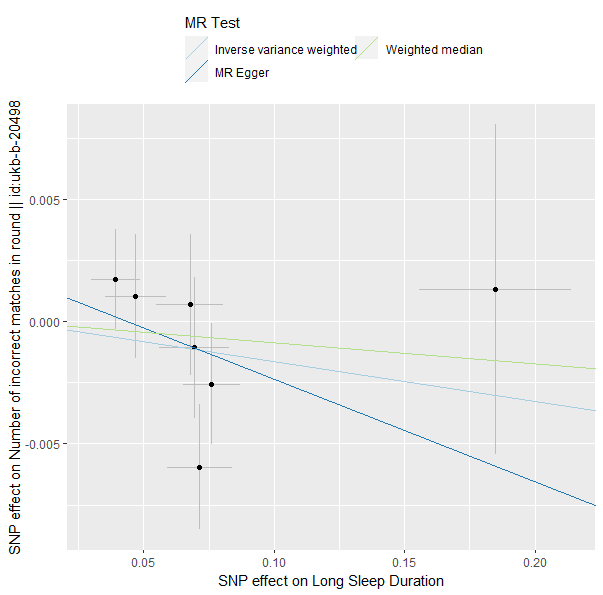

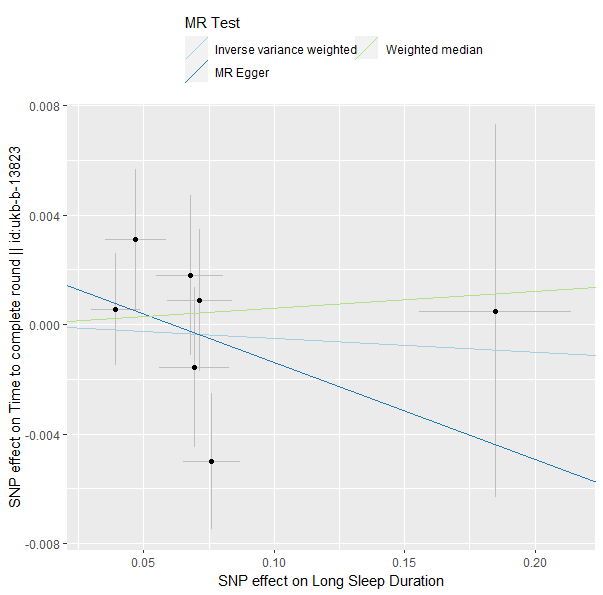


Outcome：Reaction Time


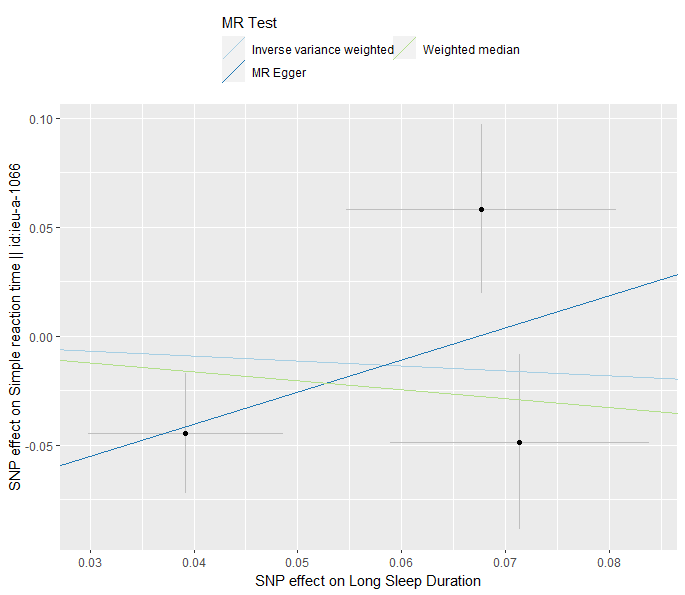


Outcome: Alzheimer's Disease

Lewy Body Dementia (LBD)

Vascular dementia


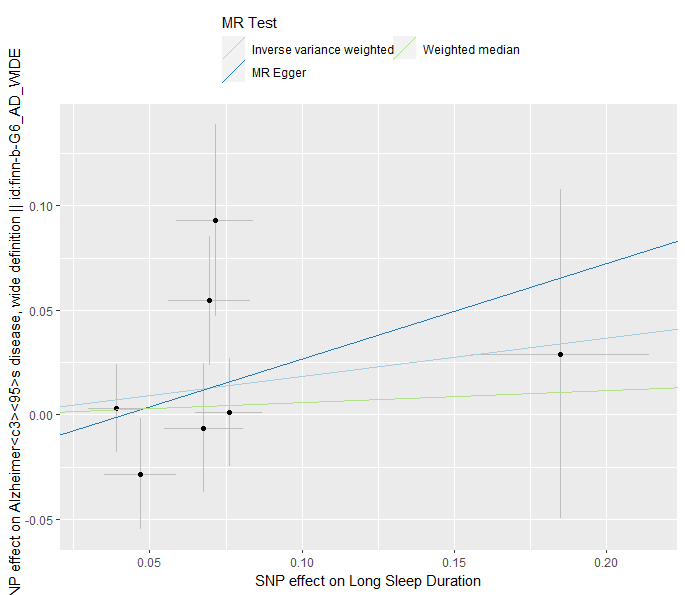

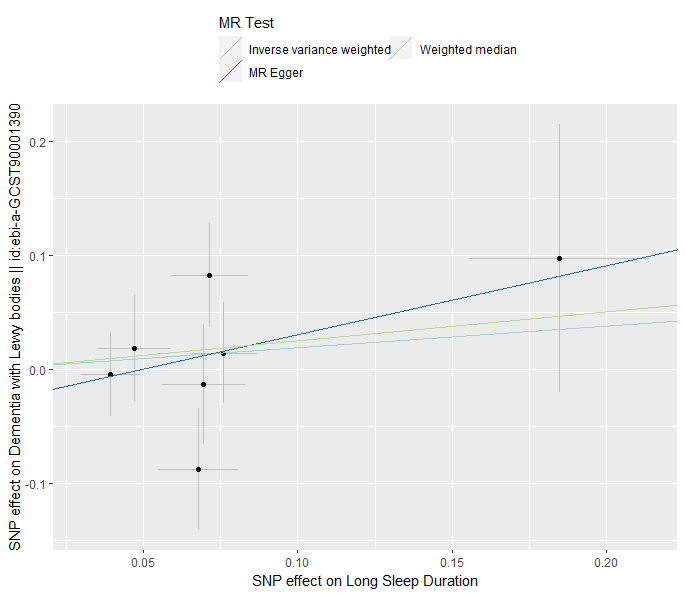


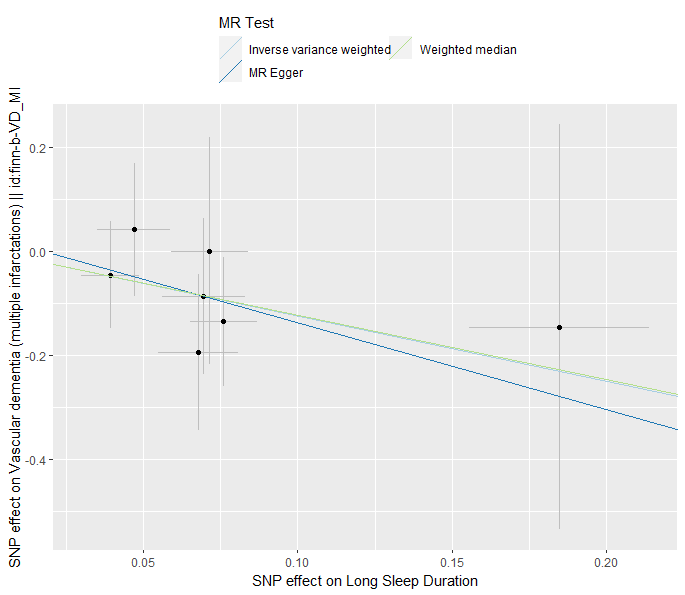


**Supplement Fig II *Funnel plots for MR analysis of sleep phenotypes and cognitive functions***

**Exposure: Short sleep duration**

Outcome: Cognitve Performance

Fluid intelligence score (FIS)

Memory Performance


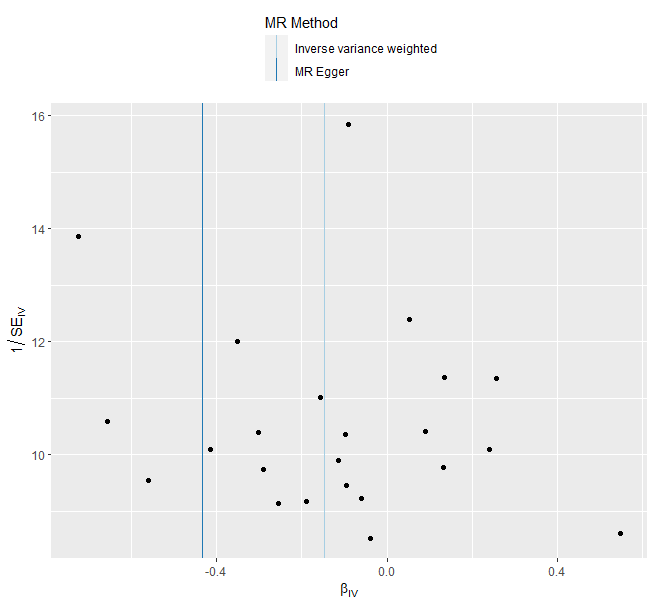

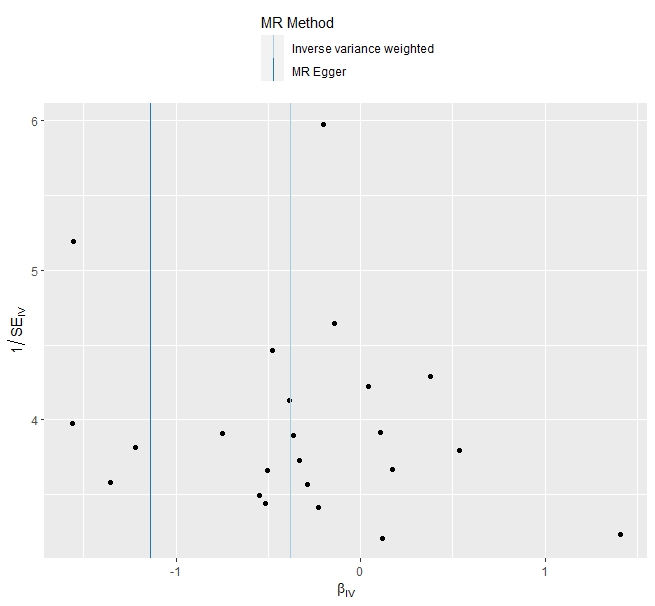

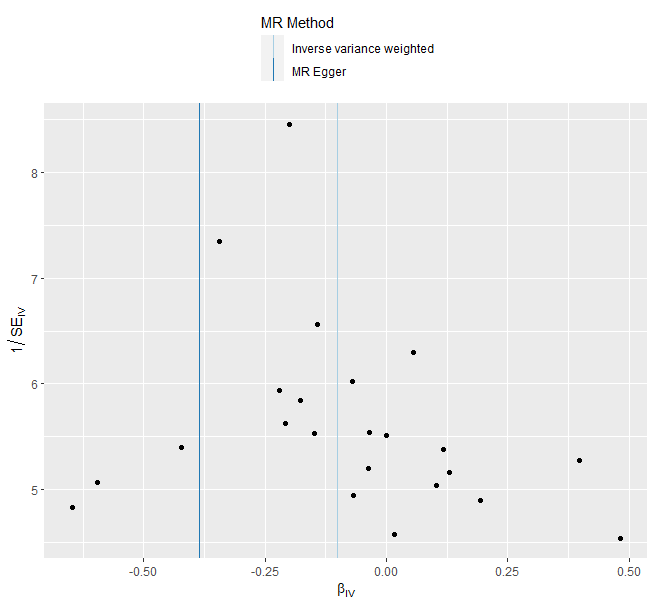


Outcome: TM: Interval in trail 2 path

TM: Duration to complete trail 2 path


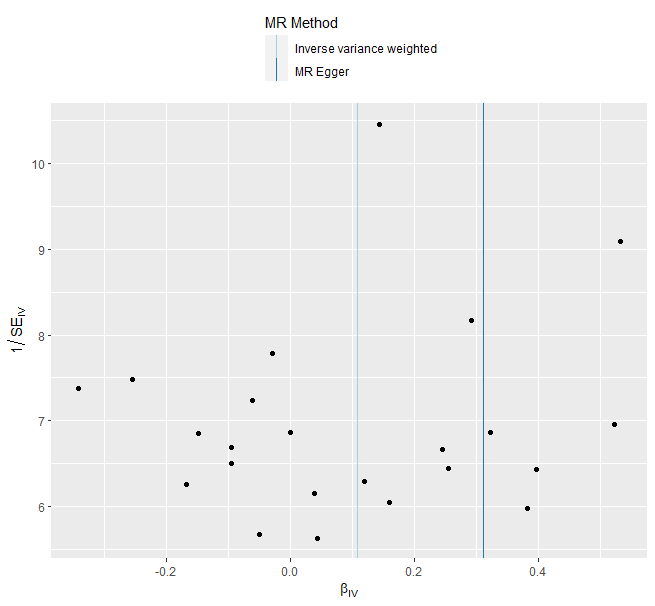

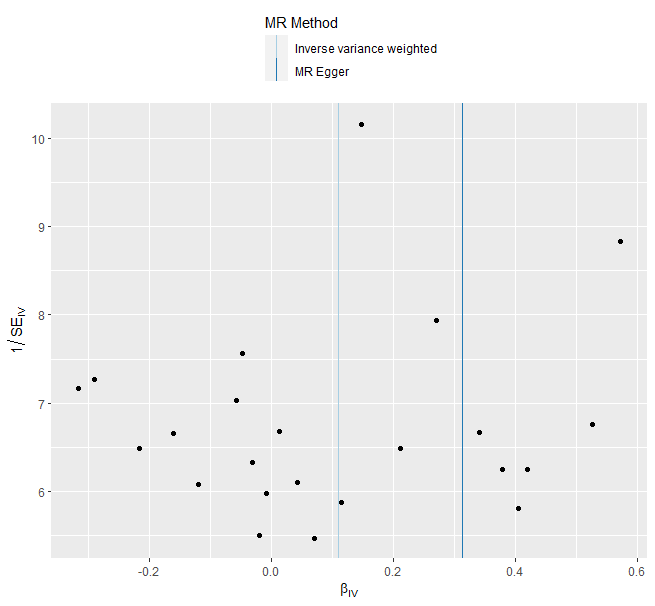


Outcome: SDS: Number of correct matches

SDS: Number of matches attempted

SDS: Duration to entering value


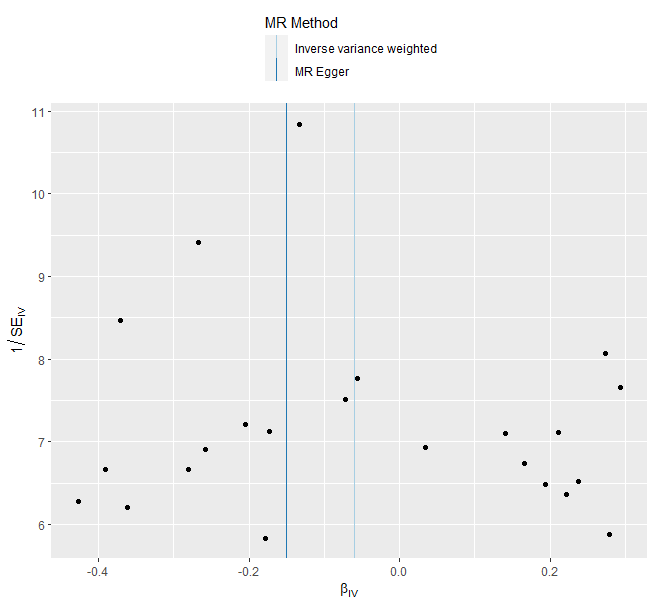

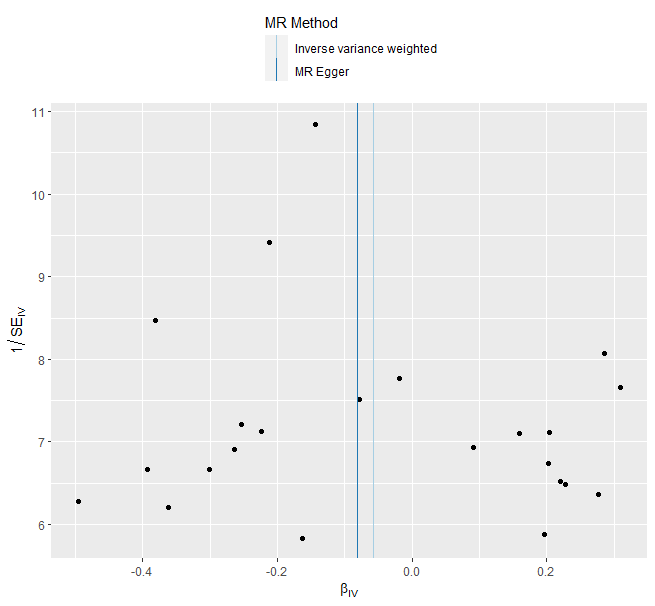

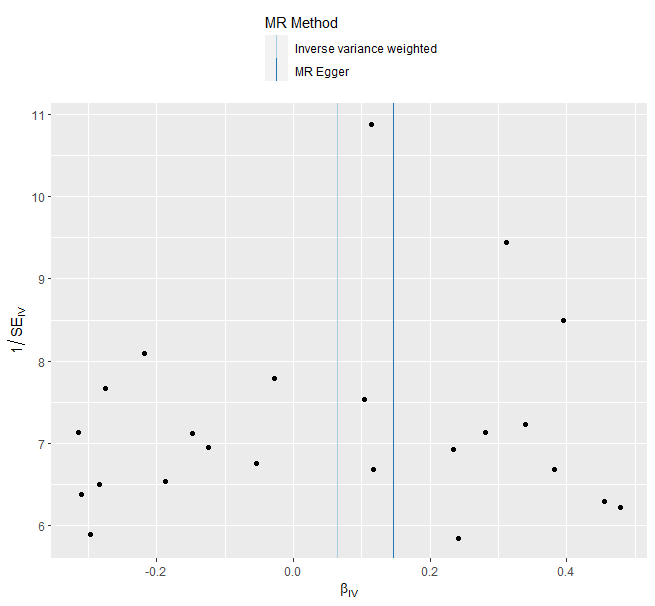


Outcome: PM: Number of incorrect matches

PM: Time to complete round


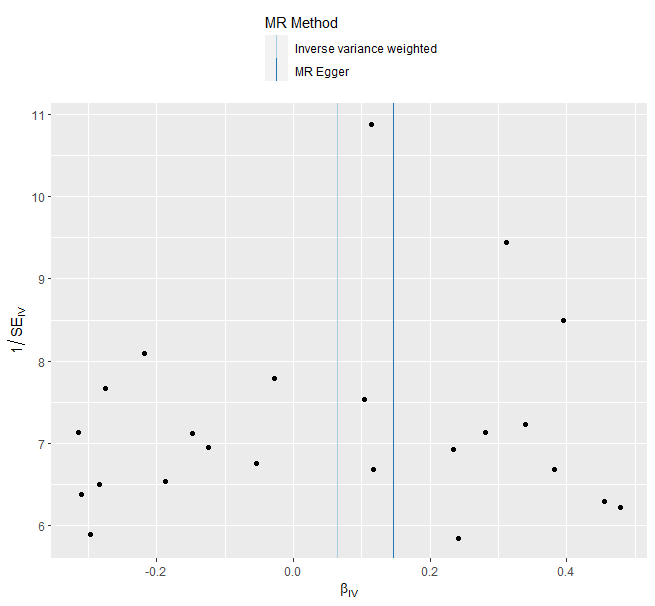

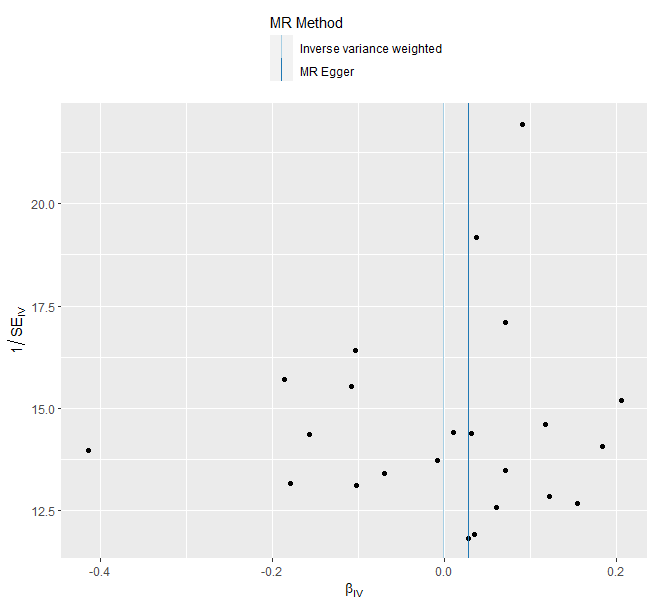


Outcome：Reaction Time


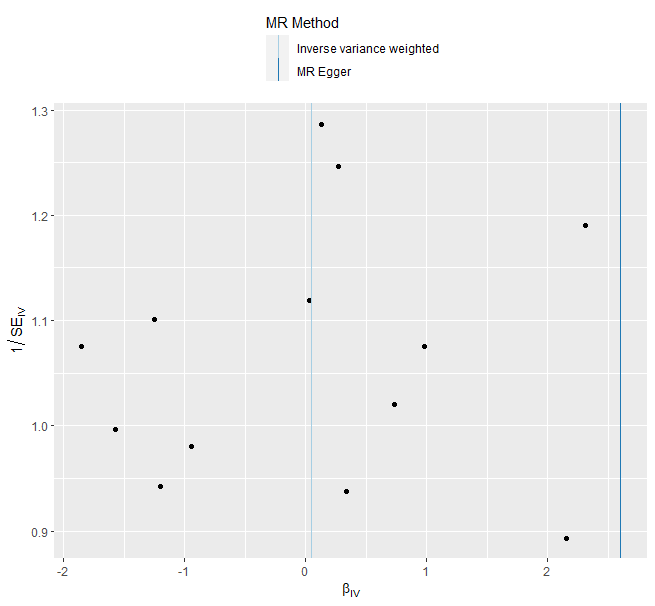


Outcome: Alzheimer's Disease

Lewy Body Dementia (LBD)

Vascular dementia

Frontotemporal dementia


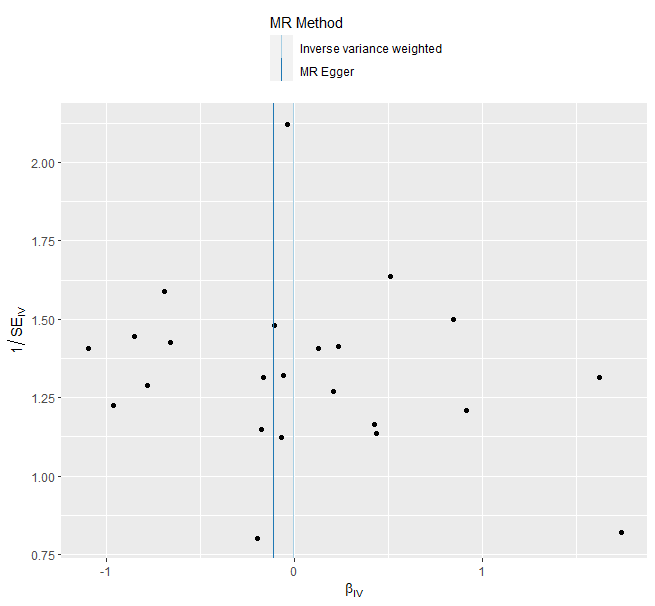

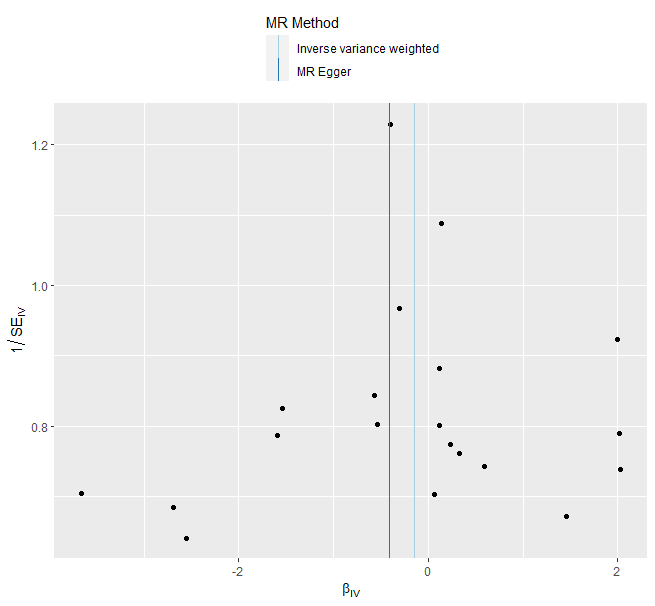


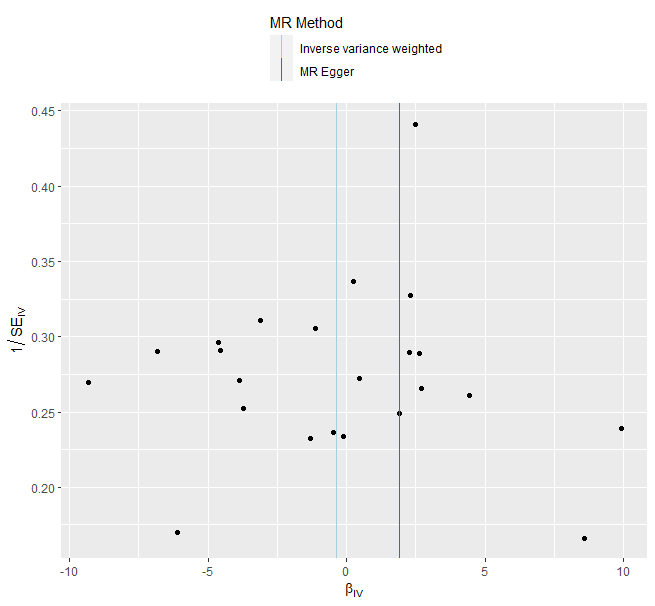

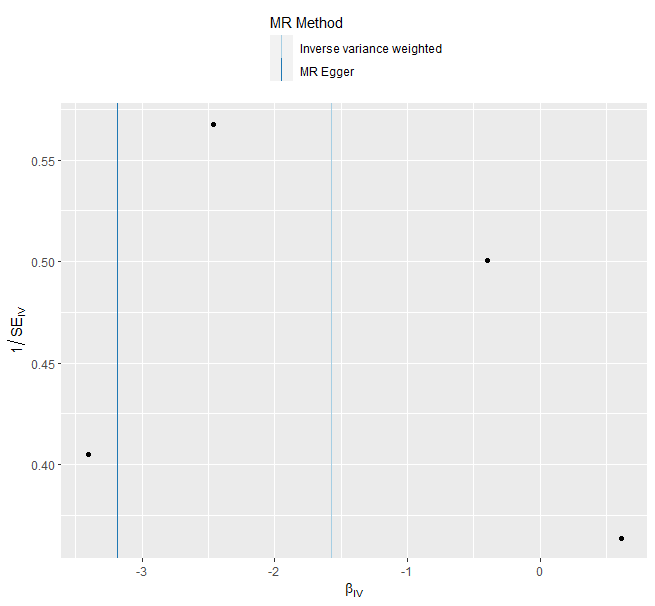


**Exposure: Insomnia**

Outcome: Cognitve Performance

Fluid intelligence score (FIS)

Memory Performance


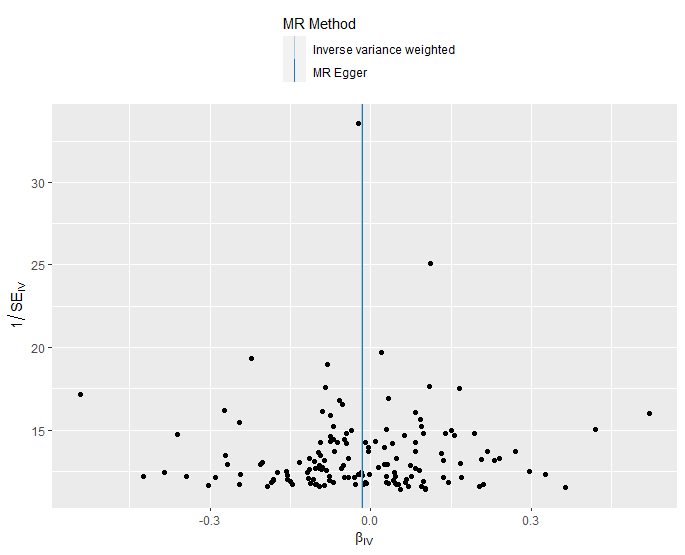

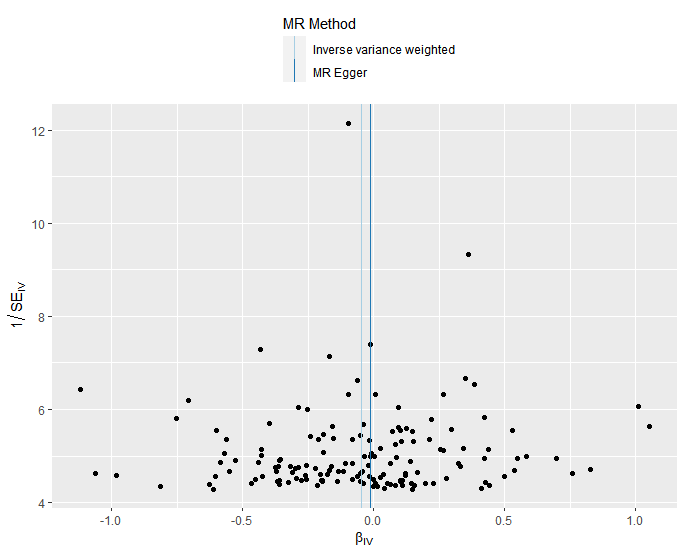

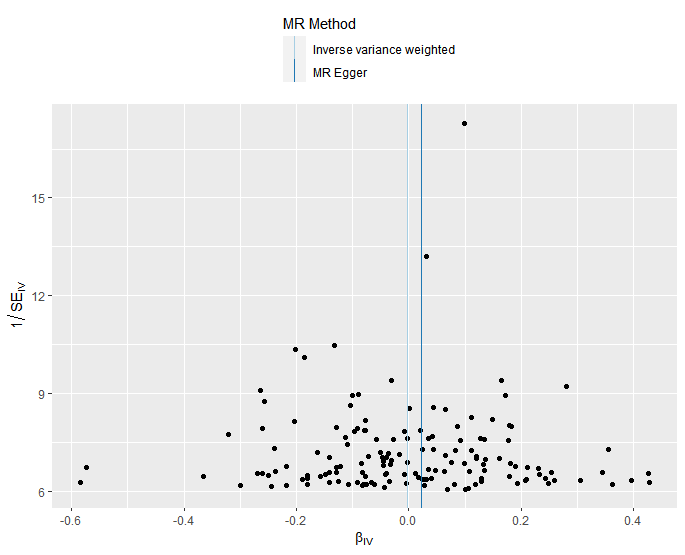


Outcome: TM: Interval in trail 2 path

TM: Duration to complete trail 2 path


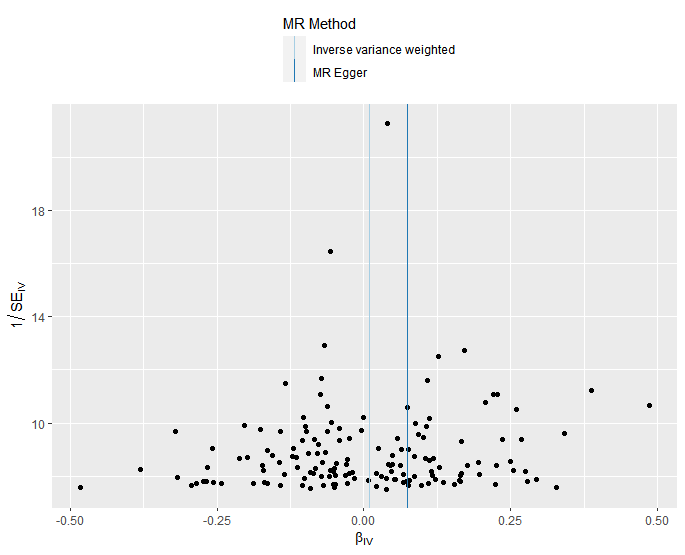

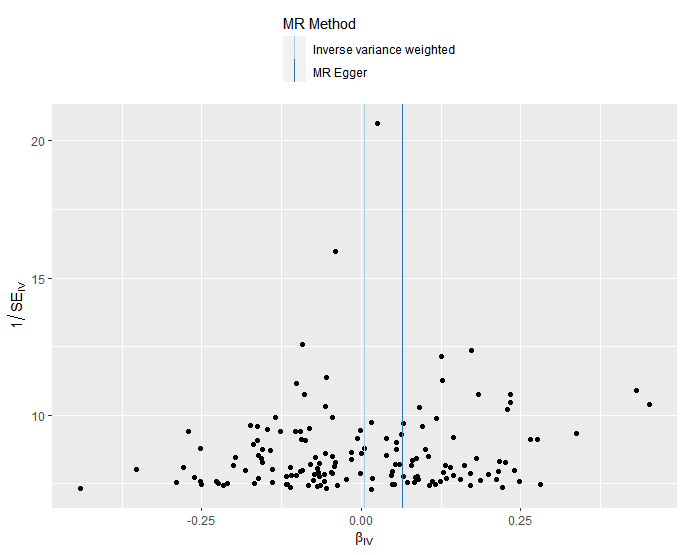


Outcome: SDS: Number of correct matches

SDS: Number of matches attempted

SDS: Duration to entering value


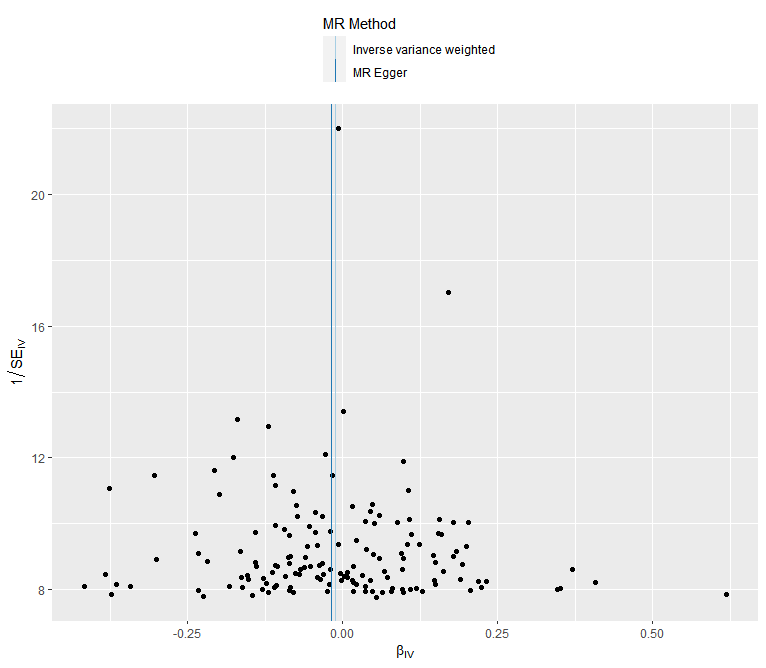

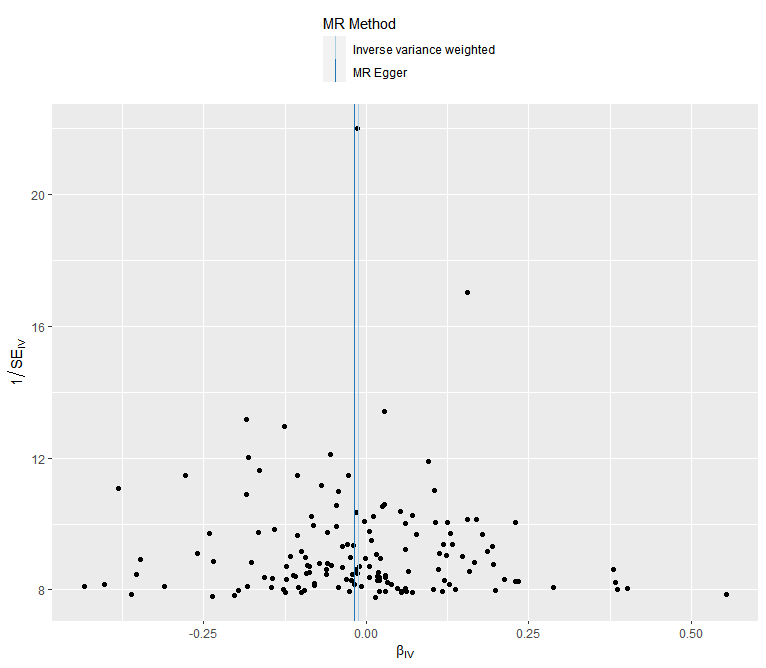

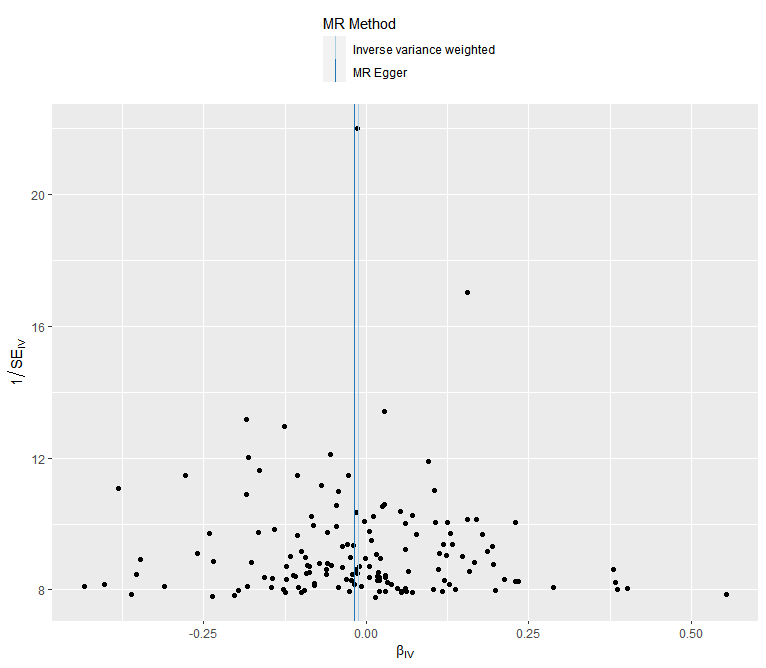


Outcome: PM: Number of incorrect matches

PM: Time to complete round


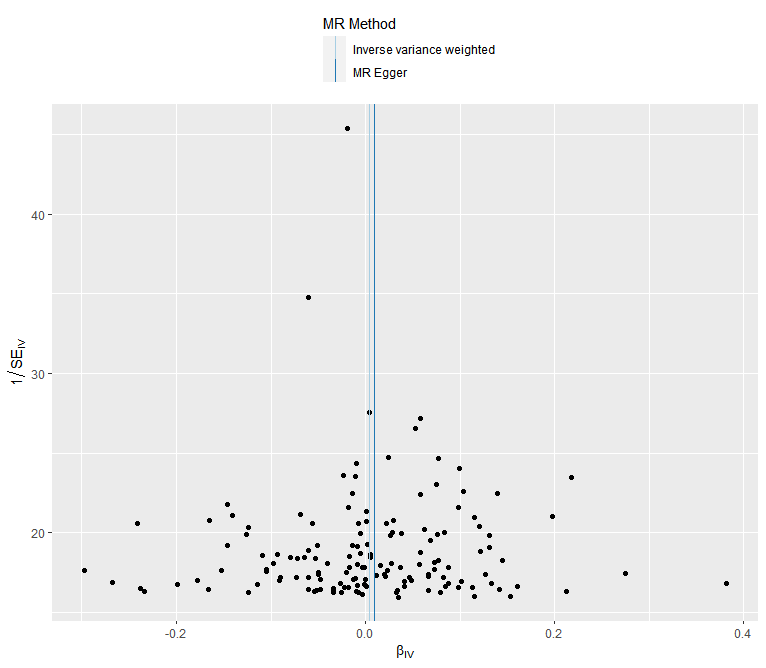

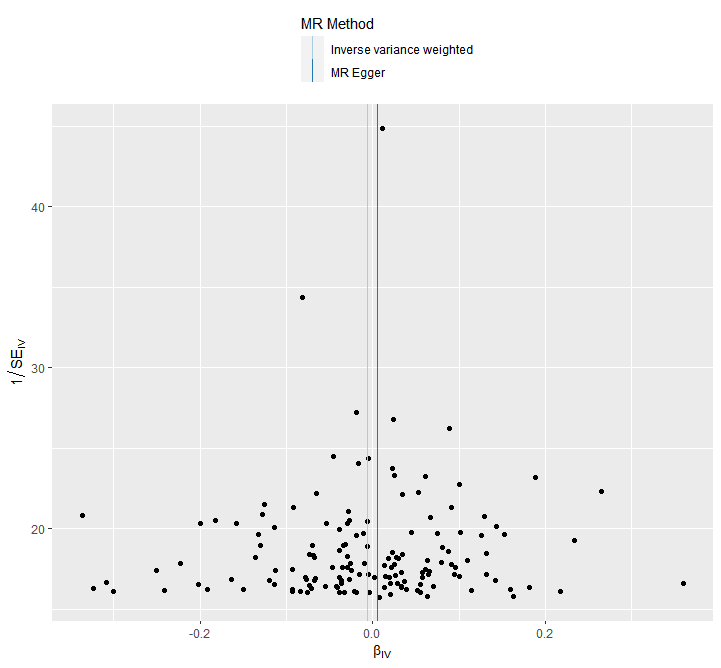


Outcome：Reaction Time


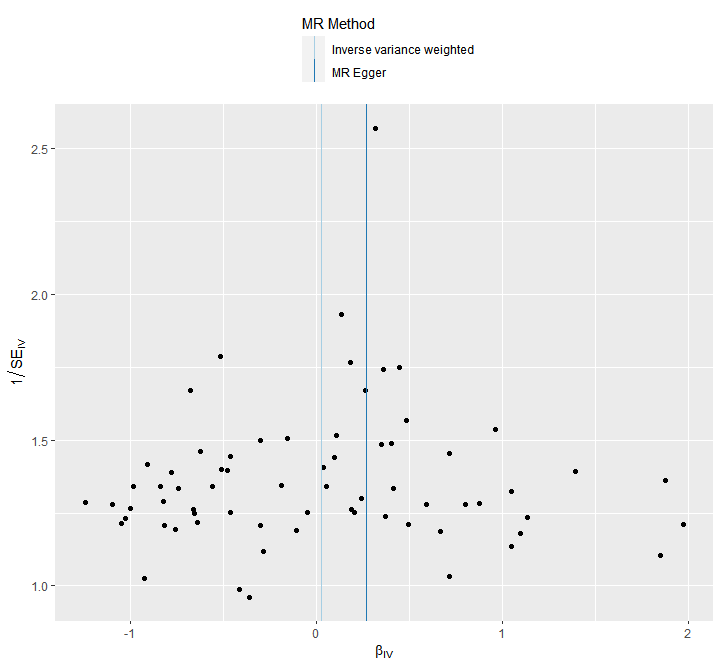


Outcome: Alzheimer's Disease

Lewy Body Dementia (LBD)

Vascular dementia

Frontotemporal dementia


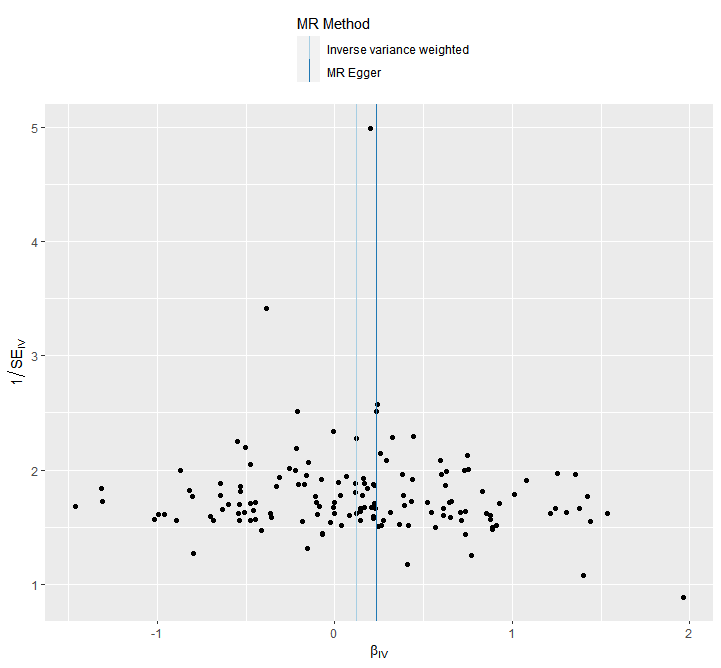

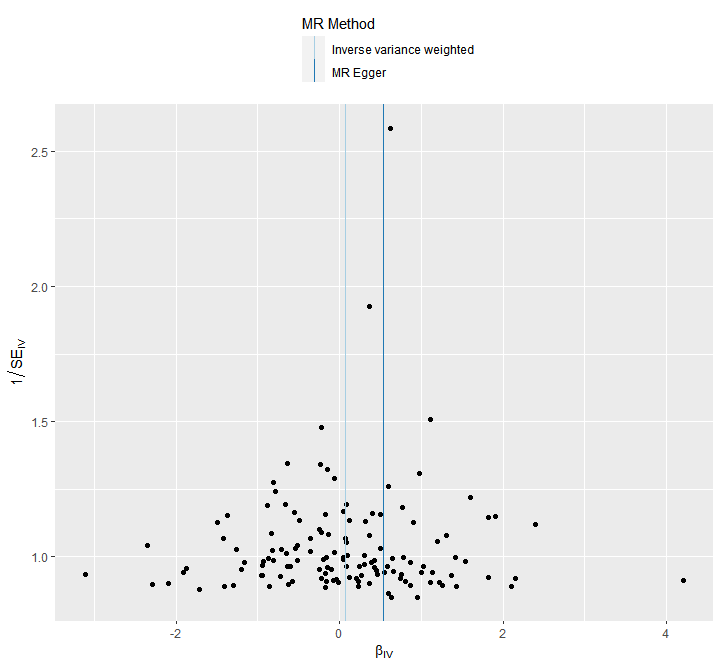


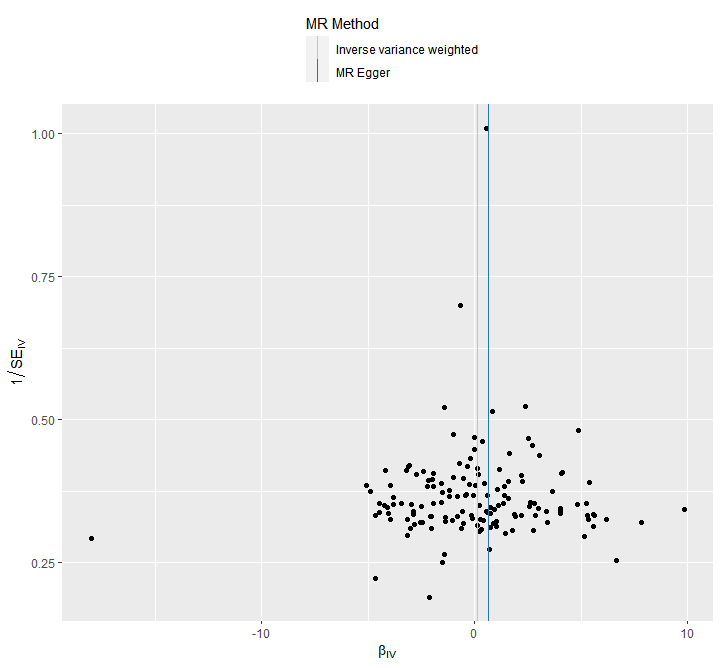

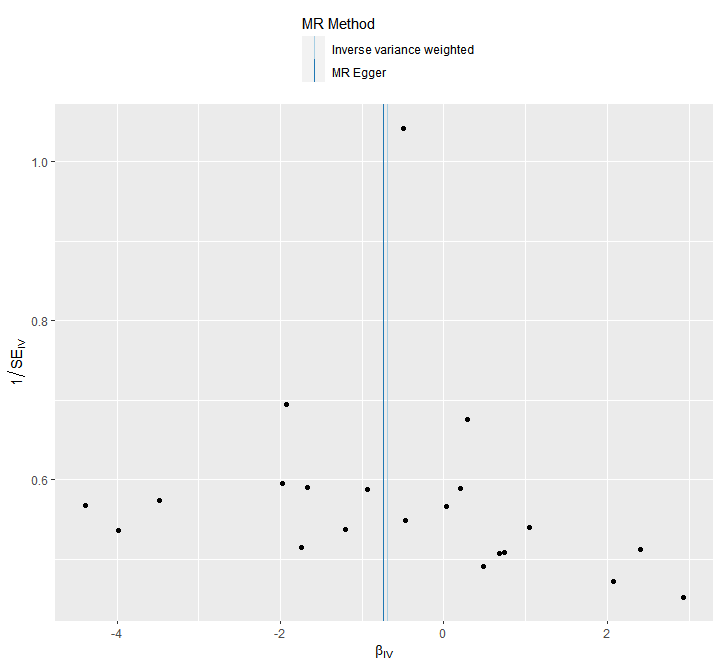


**Exposure: Long sleep duration**

Outcome: Cognitve Performance

Fluid intelligence score (FIS)

Memory Performance


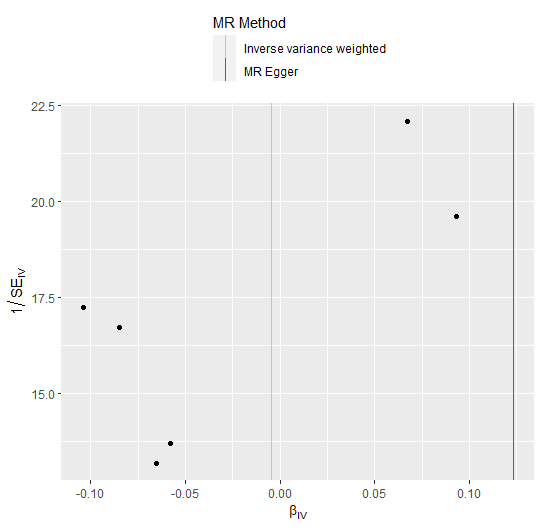

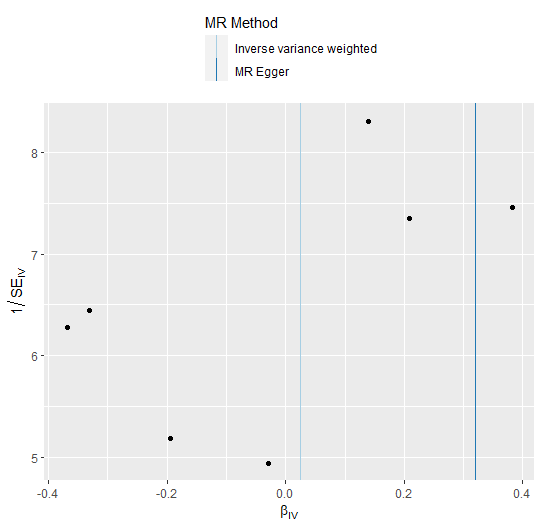

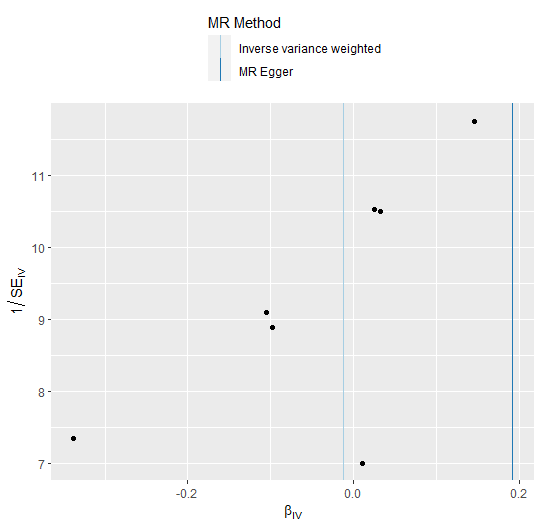


Outcome: TM: Interval in trail 2 path

TM: Duration to complete trail 2 path


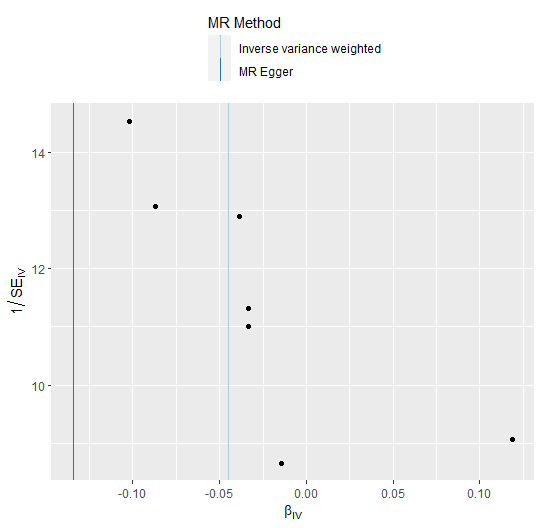

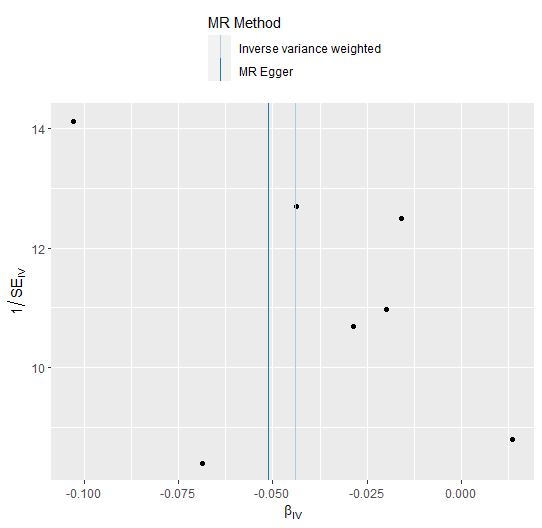


Outcome: SDS: Number of correct matches

SDS: Number of matches attempted

SDS: Duration to entering value


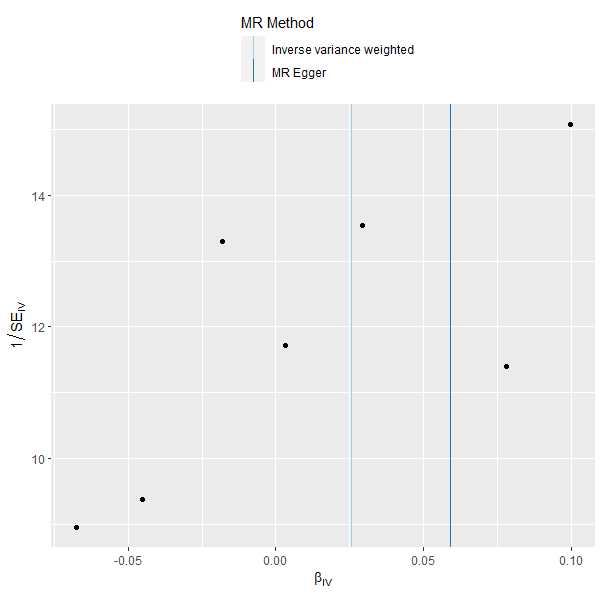

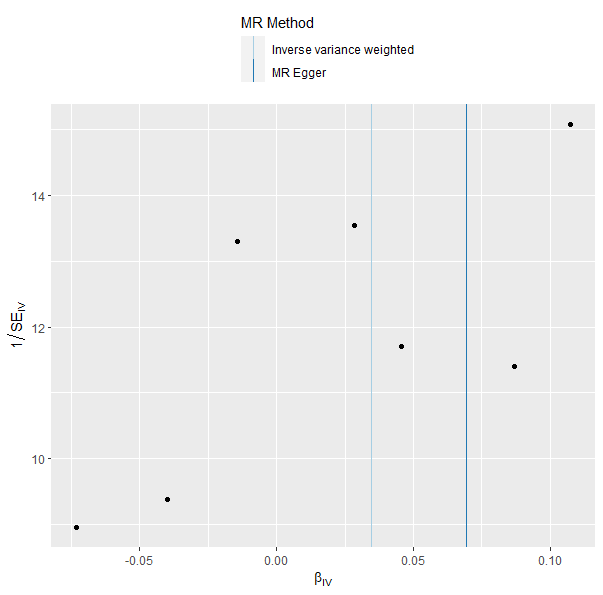

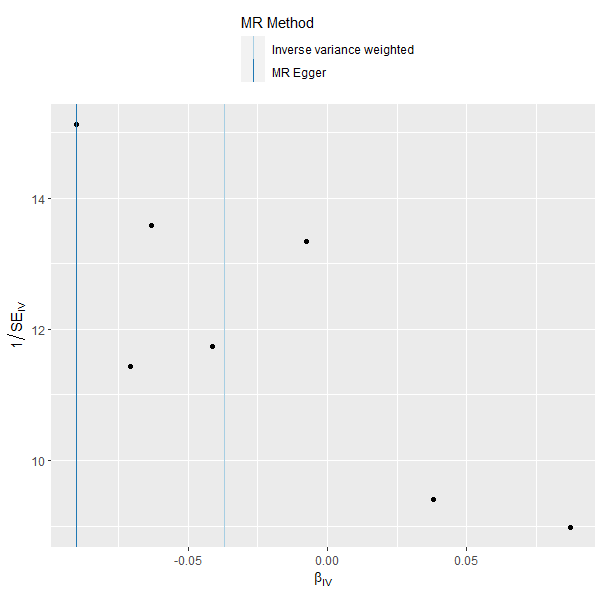


Outcome: PM: Number of incorrect matches

PM: Time to complete round


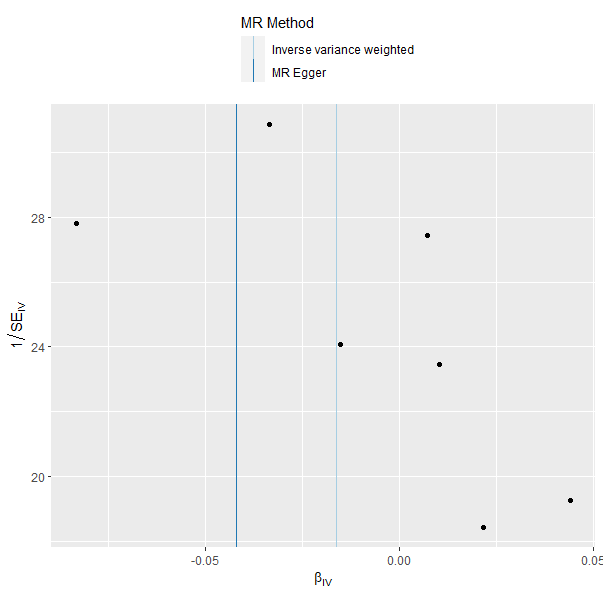

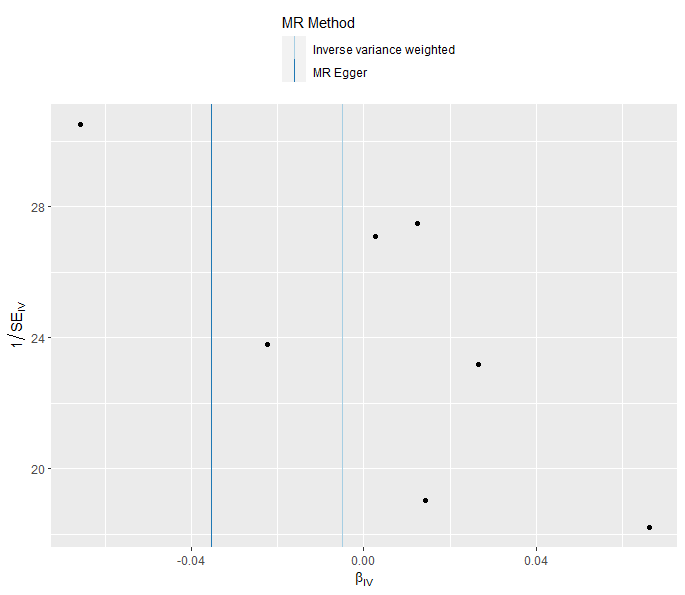


Outcome：Reaction Time


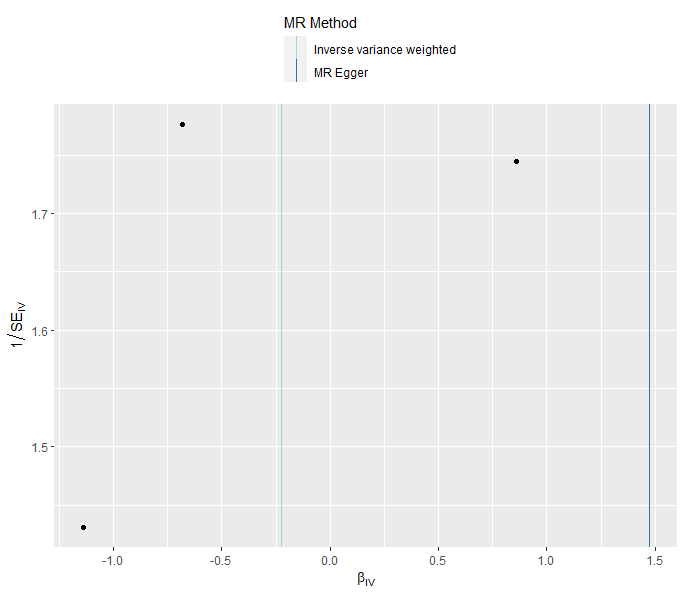


Outcome: Alzheimer's Disease

Lewy Body Dementia (LBD)

Vascular dementia


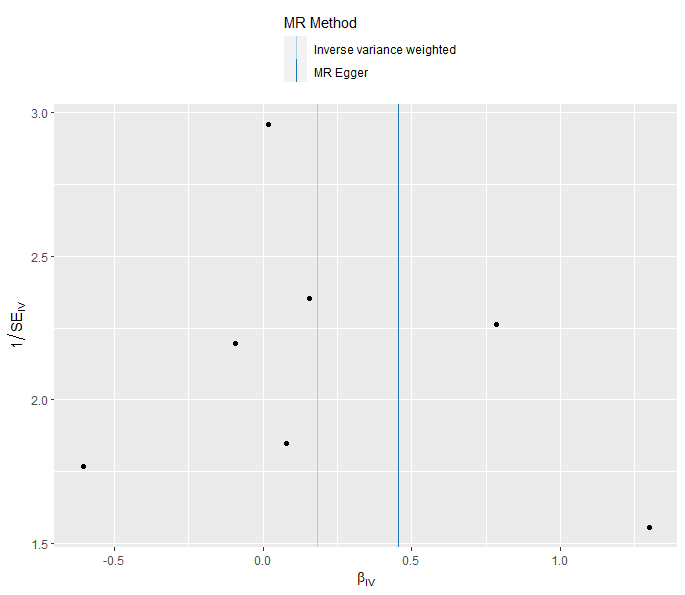

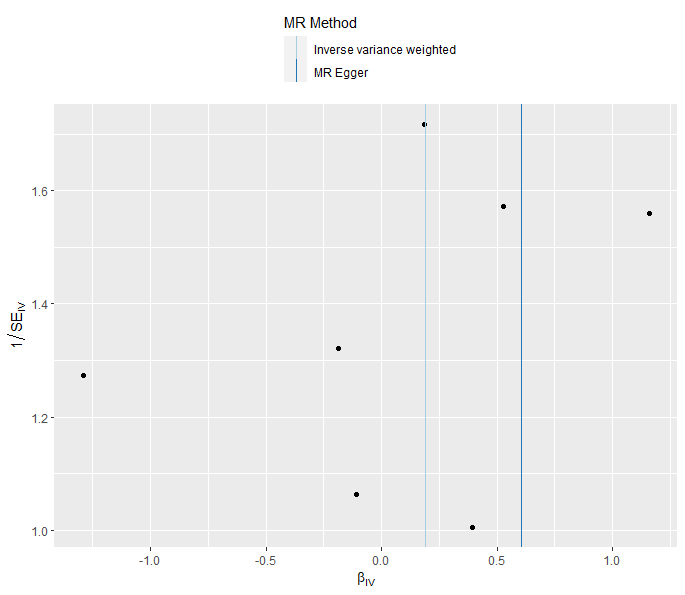


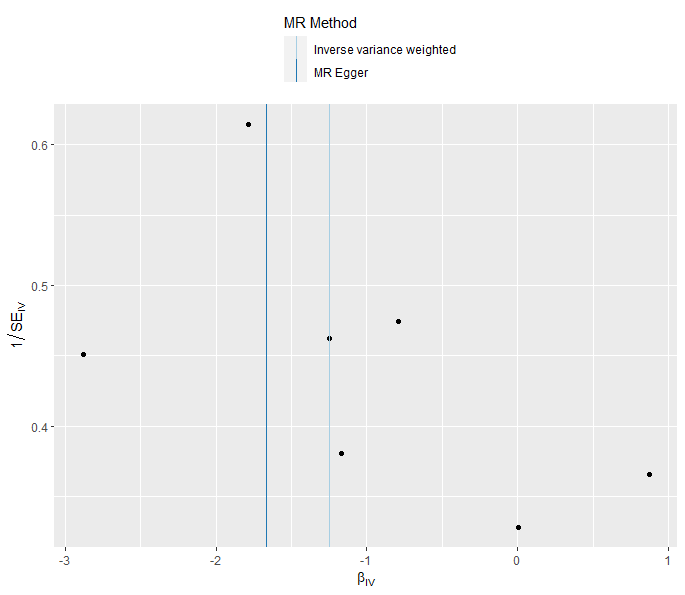


**Supplement Fig III *Leave-one-out analysis for MR analysis of sleep phenotypes and cognitive functions***

**Exposure: Short sleep duration**

Outcome: Cognitve Performance

Fluid intelligence score (FIS)

Memory Performance


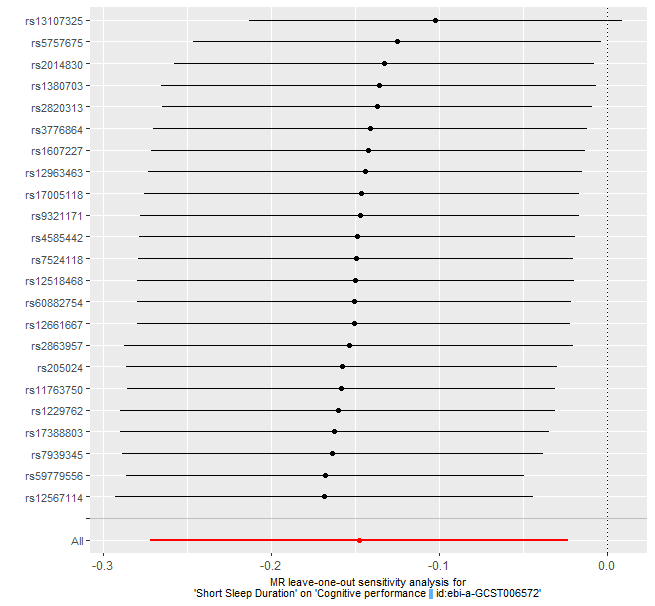

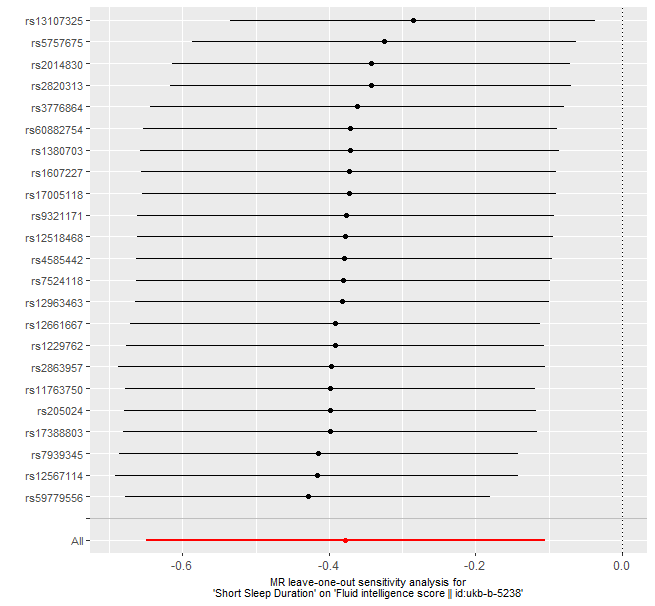

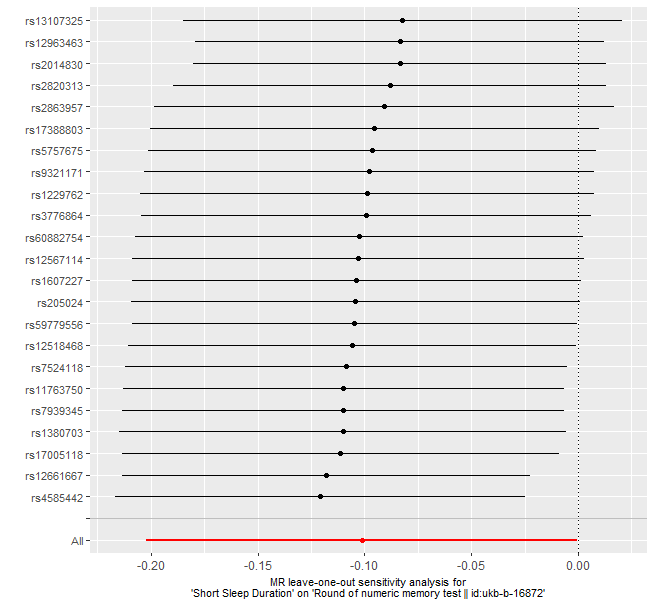


Outcome: TM: Interval in trail 2 path

TM: Duration to complete trail 2 path


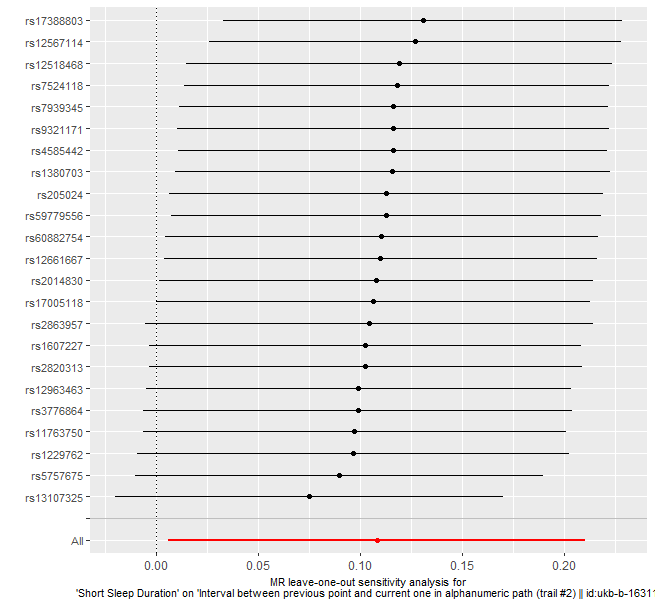

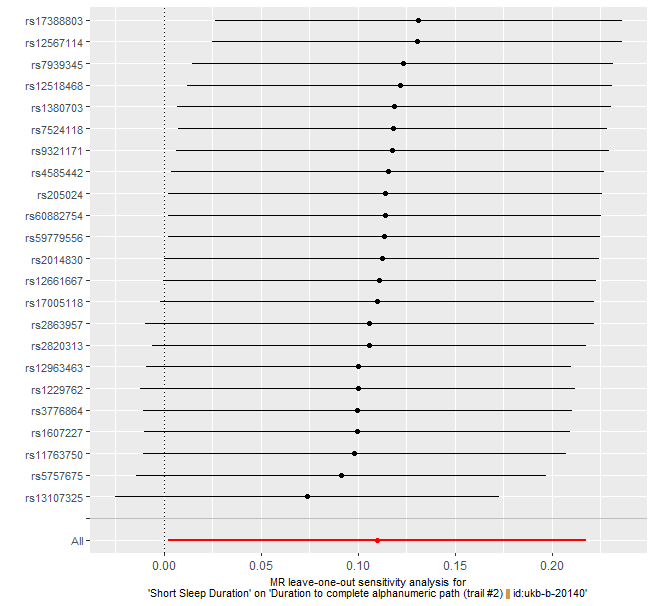


Outcome: SDS: Number of correct matches

SDS: Number of matches attempted

SDS: Duration to entering value


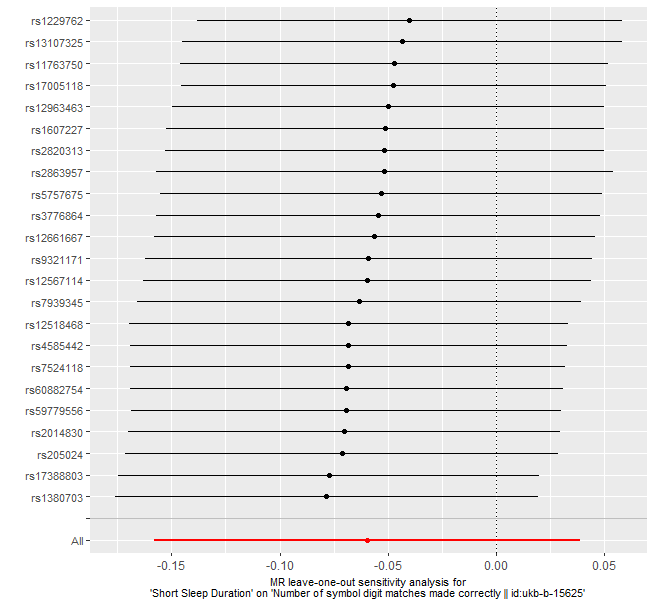

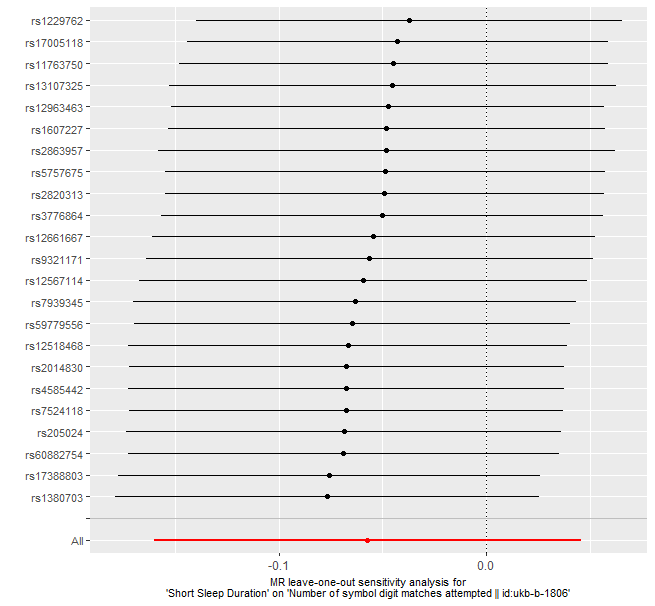

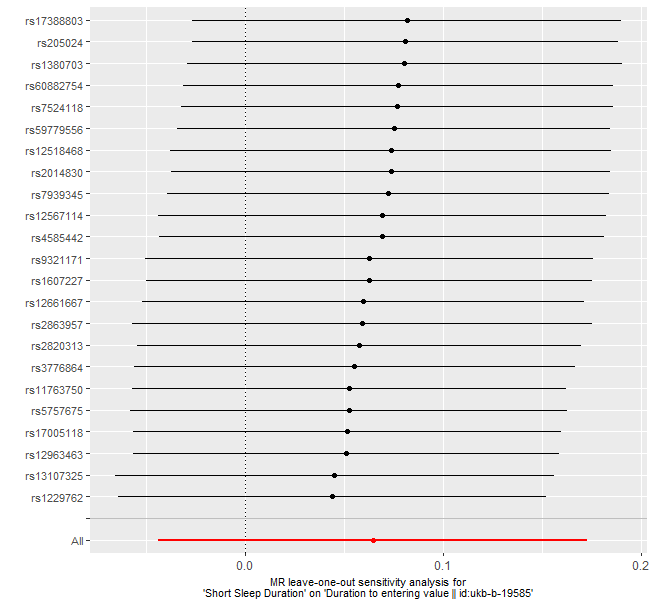


Outcome: PM: Number of incorrect matches

PM: Time to complete round


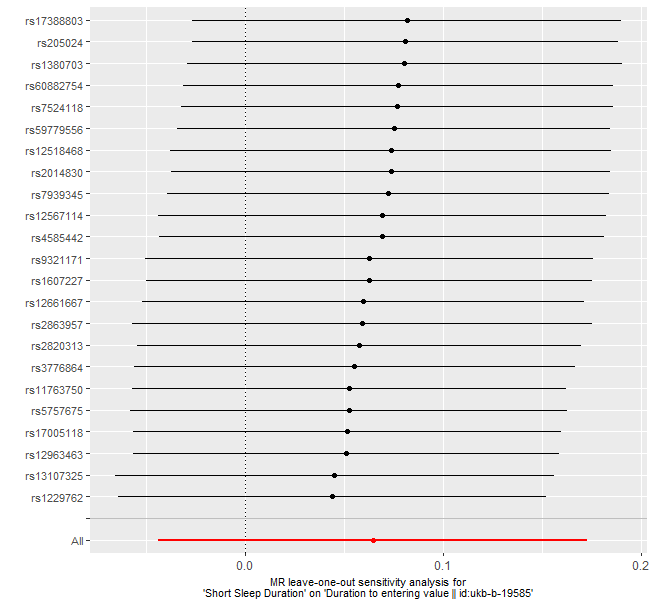

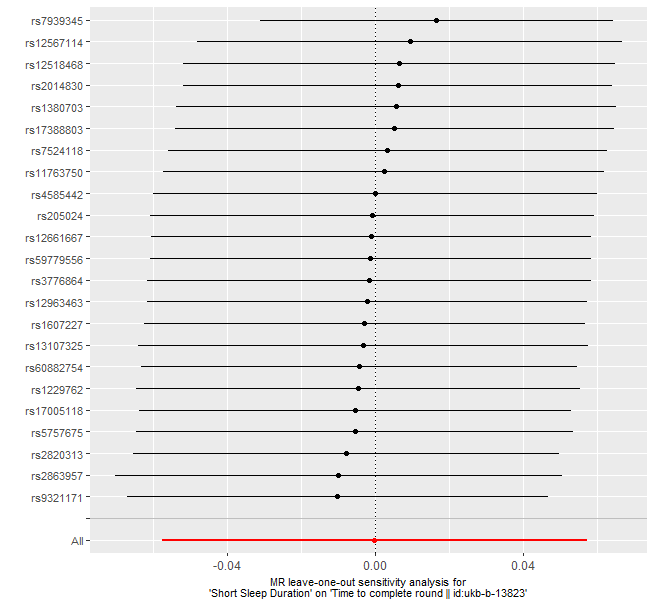


Outcome：Reaction Time


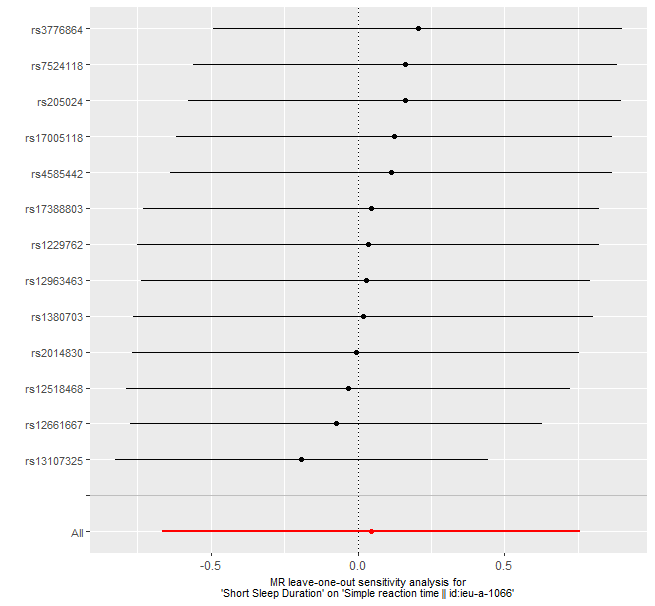


Outcome: Alzheimer's Disease

Lewy Body Dementia (LBD)

Vascular dementia

Frontotemporal dementia


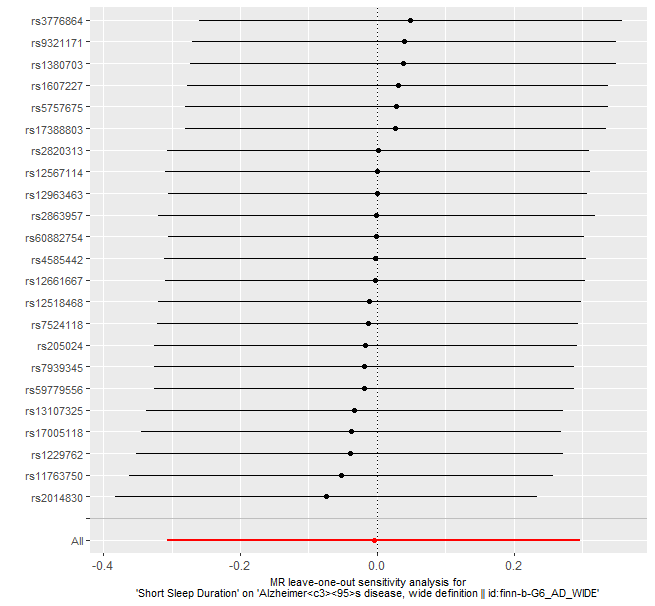


**Exposure: Insomnia**

Outcome: Cognitve Performance

Fluid intelligence score (FIS)

Memory Performance

Outcome: TM: Interval in trail 2 path

TM: Duration to complete trail 2 path

Outcome: SDS: Number of correct matches

SDS: Number of matches attempted

SDS: Duration to entering value

Outcome: PM: Number of incorrect matches

PM: Time to complete round

Outcome：Reaction Time

Outcome: Alzheimer's Disease

Lewy Body Dementia (LBD)

Vascular dementia

Frontotemporal dementia

**Exposure: Long sleep duration**

Outcome: Cognitve Performance

Fluid intelligence score (FIS)

Memory Performance

Outcome: TM: Interval in trail 2 path

TM: Duration to complete trail 2 path

Outcome: SDS: Number of correct matches

SDS: Number of matches attempted

SDS: Duration to entering value

Outcome: PM: Number of incorrect matches

PM: Time to complete round

Outcome：Reaction Time

Outcome: Alzheimer's Disease

Lewy Body Dementia (LBD)

Vascular dementia
