# Supplementary material for: Mendelian randomization study of self-reported long sleep duration, short sleep duration, and insomnia and cognitive function
Source: PLoS One. 2025 Aug 20;20(8):e0330782. doi: 10.1371/journal.pone.0330782 (PMC12367179; doi:10.1371/journal.pone.0330782)
Supplement: Attachment — Submitted filename: Review of PONE-D-24-46332.docx [file pone.0330782.s003.docx]

To the editors,

Thank you for your give me the opportunity to review the manuscript entitled (Mendelian Randomization Study of Self-reported long sleep duration, short sleep duration, and Insomnia and Cognitive Function).

The manuscript focuses on the self -reported measures of sleep durations with the cognition, using the genetic variations as indirect causative factors in losing the mental abilities.

This study may have several limitations:

1- The genetic variations in the SNPs determined by this study were already related to loss mental abilities by dementia or Alzheimer, therefor, the reliance to self-reporting duration to measure the cognitive may be not accurate and lead to biased results.
2- The study restricted the randomized analysis on the European ancestry population. So, the results may not match with variation in another ancestry populations.
3- The genetic variations influence sleep duration is unclear and affected by several environmental factors, sex, age …etc. therefor it’s not enough to be decisive results to link the cognitive with sleep duration.

Best regards

Dr. Luma Hassan Alwan Al Obaidy

20/3/2025
