# Supplementary material for: Mendelian randomization study of self-reported long sleep duration, short sleep duration, and insomnia and cognitive function
Source: PLoS One. 2025 Aug 20;20(8):e0330782. doi: 10.1371/journal.pone.0330782 (PMC12367179; doi:10.1371/journal.pone.0330782)
Supplement: Attachment — Submitted filename: Response to Reviewers.docx [file pone.0330782.s005.docx]

**PONE-D-24-46332**

**Mendelian Randomization Study of Self-reported Long sleep duration, Short sleep duration, and Insomnia and Cognitive Function**

Thank you for the opportunity to revise and resubmit this manuscript. The comments and suggestions provided by reviewers have been very helpful in guiding the revision. The manuscript have been revised thoroughly in accordance with recommendations of the reviewers. A detailed summary of the revisions was provided in the accompanying memo. I believe that the manuscript has improved significantly as a result of the review process, and hope that the revised study is suitable for a potential publication in *PLOS One.*

**ACADEMIC EDITOR**

**When submitting your revision, we need you to address these additional requirements.**

1. **Please ensure that your manuscript meets PLOS ONE's style requirements, including those for file naming.**

I have revised the manuscript to conform to PLOS ONE’s style requirements, including formatting and file naming, following the templates provided.

2. **PLOS requires an ORCID iD for the corresponding author in Editorial Manager on papers submitted after December 6th, 2016. Please ensure that you have an ORCID iD and that it is validated in Editorial Manager. To do this, go to ‘Update my Information’ (in the upper left-hand corner of the main menu), and click on the Fetch/Validate link next to the ORCID field. This will take you to the ORCID site and allow you to create a new iD or authenticate a pre-existing iD in Editorial Manager.**

No problem! Will do.

**3. Please ensure that you refer to Figures 3-6 in your text as, if accepted, production will need this reference to link the reader to the figure.**

I have ensured that all figures, including Figures 3–6, are now properly referenced in the main text.

**4. Please include captions for your Supporting Information files at the end of your manuscript, and update any in-text citations to match accordingly. Please see our Supporting Information guidelines for more information: http://journals.plos.org/plosone/s/supporting-information.**

Captions for all Supporting Information files have been added to ***S1 Fig*** and ***S1 Table***, and in-text citations have been updated accordingly.

5. **Please review your reference list to ensure that it is complete and correct. If you have cited papers that have been retracted, please include the rationale for doing so in the manuscript text, or remove these references and replace them with relevant current references. Any changes to the reference list should be mentioned in the rebuttal letter that accompanies your revised manuscript. If you need to cite a retracted article, indicate the article’s retracted status in the References list and also include a citation and full reference for the retraction notice.**

I have updated the reference list to ensure its accuracy and completeness. No retracted articles are cited. Any references that were added or modified are described below.

**REVIEWER 1**

**The manuscript aims to determine whether self‐reported sleep phenotypes—specifically short sleep duration, long sleep duration, and insomnia—are causally linked to cognitive function and dementia risk using a two-sample Mendelian randomization (MR) design. Overall, the study is methodologically sound and addresses an important public health question. The selection of genetic instruments from well-powered GWAS datasets and the use of multiple MR methods are notable strengths. However, some issues should be addressed before publication:**

Thank you for your thorough review of our manuscript and positive and encouraging feedback. I appreciate the time that you have put in to it. I also appreciate the concerns you have raised. To that end, I have addressed each of your concerns, and I believe that the revised manuscript is stronger as a result. Below are responses to each of your comments. I also added a list of references at the end of your comments. Revisions within the manuscript are highlighted in blue.

**1. Figures and Results Description:**

**• The main text would benefit from more detailed commentary on the results shown in Figures 3, 4, 5, and 6. Clearly explain how the associations differ among short sleep duration, long sleep duration, and insomnia across the various cognitive outcomes.**

Thank you for this suggestion. I have added more detailed commentary on the results presented in Figures 3, 4, 5, and 6. This includes highlighting patterns of directionality, magnitude of effect sizes, and statistical significance. The details are as follows：

*“The findings suggest that short sleep duration may adversely affect multiple domains of cognitive function. MR analysis using an IVW method showed that short sleep duration showed a harmful impact on cognitive performance (beta = –0.15, 95% CI: –0.27 to –0.02, P = 0.02), which serve as a potential test for identifying people in the community with undiagnosed dementia, and their decline reflects a significant reduction in overall cognitive function. Similarly, there was a strong negative correlation between short sleep duration and FIS (beta = –0.38; 95% CI: –0.65 to –0.11; P = 0.006, IVW). The FIS is a physiologically based measure of cognitive ability related to arithmetic speed and reasoning. In terms of memory function, short sleep duration was linked to poorer memory performance (beta = –0.10, 95% CI: –0.20 to –0.0005; P = 0.04, IVW), further underscoring the broad cognitive impact of inadequate sleep (Figure 3).*

*As shown in Figure 3, performance on the TM test, which assesses executive function and the ability to switch attention, was also negatively affected. Specifically, short sleep duration was associated with a longer interval between steps in the Trail 2 path (beta = 0.11, 95% CI: 0.01 to 0.21, P = 0.03, IVW) and a longer total duration to complete the Trail 2 path (beta = 0.11, 95% CI: 0.002 to 0.22, P = 0.04, IVW), suggesting impairments in attention and task-switching capabilities.*

*There was insufficient evidence to support a causal relationship between insomnia and performance on cognitive tests, including FIS (Figure 4). In addition, insomnia was causally associated with AD, with a significant odds ratio of 1.13 (95% CI: 1.02 to 1.24, P = 0.02, IVW) (Figure 6). However, there was insufficient evidence to support a causal relationship between long sleep duration and every cognitive-related outcome (P > 0.05 for all) (Figure 5 and Figure 6).* ”

Please refer to 3.2 Primary analysis.

**• Please check that all the Figures are referenced in the main text, as this improves readability.**

I have also carefully checked the manuscript to ensure that all figures are explicitly referenced in the main text at appropriate points, in order to improve readability and coherence.

Kindly refer to 3.2 Primary analysis.

**2. Interpretation of Sleep Phenotypes:**

**• Provide additional insights into why short sleep duration appears to have a stronger association with certain cognitive measures (e.g., fluid intelligence) compared to insomnia, and discuss how these different sleep measures may influence cognitive outcomes via distinct biological pathways.**

I appreciate this insightful suggestion regarding the interpretation of the associations. Indeed, short sleep duration seems to be more strongly associated with certain cognitive indicators (e.g., fluid intelligence) than insomnia. This may be due to differences in the definitions of short sleep duration and insomnia. In this study, short sleep duration was defined as 6 hours or less of sleep per 24 hours. Shorter sleep duration implies a reduction in slow wave sleep (SWS) and rapid eye movement (REM) sleep.The emergence and integrity of SWS are strongly associated with fact-dependent situational memory capacity (1). After controlling for the effects of demographic factors, studies have found an association between SWS sleep and information processing speed; meanwhile, REM sleep significantly affects an individual's executive function performance (2). Insomnia is defined as difficulty falling asleep at night or waking up in the middle of the night. Thus, insomniacs may still obtain a normal total sleep duration, albeit with poorer sleep quality. Alternatively, insomniacs may be more conscious of this compensation through planned daytime naps, but chronically sleep-deprived individuals tend to underestimate their cognitive deficits. During planned daytime naps, individuals may not only compensate for REM sleep, but also compensate for reduced SWS by prolonging REM sleep, thus demonstrating resilience to SWS deficits. From this perspective, some of the functions of SWS sleep may be redistributed during REM sleep stages or compensated to some extent by REM sleep-related processes (2). Second, sleep duration in this study was supported by accelerometer estimates. Insomnia relies on subjective self-reports, and subjective symptoms are influenced by mood, anxiety, and personal distress, which complicates its connection to objective cognition. In contrast to disease diagnosis, fluid intelligence emphasises speed of information processing, ability to reason abstractly and executive function. Thus, fluid intelligence scores better capture the changes in cognitive functioning that occur as a result of reduced SWS and REM due to short sleep duration. The above has been added to the seventh paragraph of the Discussion.

In addition to the fact that sleep duration affects cognitive function by influencing SWS and REM emergence and integrity, I have added the biological mechanisms by which sleep disruption or fragmentation due to insomnia affects cognitive health by influencing neuroinflammation and cerebral lymphatic system clearance. It has been added as follows:

*“Beyond sleep duration, growing evidence suggests that sleep quality plays a critical role in brain health through its effects on neuroinflammation and glymphatic clearance. Disrupted or fragmented sleep has been shown to increase systemic and central nervous system levels of pro-inflammatory cytokines, such as interleukin-6 (IL-6) and tumor necrosis factor-alpha (TNF-α), which can exacerbate neuroinflammatory processes and contribute to neurodegeneration (48). Inadequate or poor-quality sleep may also impair the function of the glymphatic system—a waste clearance pathway active during sleep—thereby reducing the removal efficiency of amyloid-β and other neurotoxic proteins from the brain (47). Chronic neuroinflammation has been implicated in synaptic dysfunction, tau pathology, and accelerated cognitive decline, suggesting that sleep quality is a key modifiable factor in the pathway linking sleep disturbance to AD (49). These biological mechanisms highlight the importance of considering both the duration and quality of sleep when evaluating its causal role in cognitive aging and neurodegenerative disease.*”

Please refer to the third paragraph of the Discussion.

**• Address the non-significant findings for long sleep duration. In particular, comment on how the limited number of SNPs (6–7) used as instruments for long sleep duration might affect statistical power and precision.**

Thanks for pointing this out. This study found no evidence of statistically significant support for a causal association between long sleep duration and cognitive function. The limited number of SNPs used as instrumental variables for long sleep duration likely constrained the statistical power of our MR analysis. Statistical power in MR is directly influenced by the strength and number of genetic instruments, as well as the proportion of variance in the exposure they explain (3). The rigorous selection criteria chosen for this study ensured strong associations between the selected SNPs and sleep duration, thus avoiding bias from weak instrumental variables and helping to select independent genetic variants. However, when only a small number of SNPs are available, the explained variance tends to be low, which can lead to imprecise causal estimates (4). This not only reduces the likelihood of detecting true causal effects but may also result in wider confidence intervals and attenuation of effect sizes toward the null. Consequently, the null findings for long sleep duration should be interpreted with caution, as they may reflect limited instrument strength rather than a true absence of causal association.

The above has been incorporated into the Discussion section; please refer to the second second limitation point within the Discussion.

**3. Literature Context and Novelty Claims:**

**• The manuscript already provides a clear discussion of what is novel about your study—such as the inclusion of multiple cognitive outcomes and the unique methodological details—when comparing your results with those of Henry et al. (2019). To strengthen the literature context further, I recommend incorporating a discussion of Wang et al. (2023) (J. Evid. Based Med.), which has examined similar associations between sleep traits and cognitive outcomes. Acknowledging Wang et al. (2023) will help situate your findings within the broader MR literature and clarify the added value of your study.**

**• This revision will ensure that the claim of novelty is appropriately nuanced.**

Sure thing! Done! A study by Wang et al. (2023) (J. Evid. Based Med.) has been added and is discussed further along with other relevant MR analyses. The details are as follows：

*“The MR study by Wang et al. explored the potential causal relationship between sleep characteristics including sleep duration, insomnia and cognitive impairment (58). This study reported a negative correlation between self-reported sleep duration and reaction time, suggesting an adverse effect of short sleep duration on cognitive performance. This study did not find sufficient evidence to support a causal relationship between insomnia and cognitive function (58).*”

*“Five previous MR studies have brought enlightening evidence (57–61). Based on previous MR analyses, the present study further explored the causal relationship between sleep duration and cognitive functioning by further categorising sleep duration: long sleep duration and short sleep duration. In addition, the present study used insomnia GWAS data based on a larger sample size for exploration. A review of previous MR analyses on insomnia and cognitive functioning found that the study by Wang et al. used insomnia GWAS data from 386,533 individuals of European ancestry recruited from 22 assessment centres in the UK (58); the study by Huang et al. included insomnia GWAS data from 237,627 individuals of European ancestry in the UK (61). The present study used combined data collected by UK Biobank (n = 386,533 individuals) and 23andMe (n = 944,477 individuals) for a final sample size of up to 1,331,010 individuals of European ancestry. Insomnia studies with different sample sizes and study sites may yield different conclusions. On the other hand, Qiu et al.'s MR analyses considered only one test of cognitive function (59). Henry et al. judged cognitive function only by visual memory tests, reaction time and dementia or not (57). However, the manifestations of cognitive decline are complex. It is difficult to fully reflect changes in cognitive functioning and the presence of dementia using only one or two tests. Compared with the study of Henry et al, Wang et al included more cognitive tests such as cognitive performance, fluid intelligence (58). Based on previous studies, the present study added more cognitive function tests (seven cognitive tests including memory test, reaction time cognitive performance and fluid intelligence). At the same time, previous MR studies did not provide more evidence about sleep characteristics and dementia subtypes. Qiu et al.'s MR analysis did not consider any dementia and its subtypes (59); Henry et al., Yuan et al. and Huang et al. all focused only on AD, the most common form of dementia (57, 60, 61); and Wang et al.'s focus fell on AD-related progression scores and changes in brain structure (58). The present study extends beyond AD to more common subtypes of dementia, namely vascular dementia, Lewy body dementia, and frontotemporal dementia.*”

Kindly refer to the fifth and sixth paragraphs of the Discussion.

**4. Overall Discussion Flow:**

**• The discussion section appears somewhat repetitive, particularly regarding the reiteration of methodological assumptions and novelty claims. I suggest reorganizing the discussion to improve flow by consolidating these points. A more concise presentation of the core MR assumptions (relevance, independence, and exclusion restriction) and a focused discussion on the study’s limitations (e.g., reliance on self-reported sleep measures and restriction to European ancestry) would enhance readability. If these issues are already covered in other sections, consider removing or rephrasing redundant passages (for example, it is stated twice that "this is the first MR study to investigate causal associations between long sleep duration, short sleep duration, insomnia and multiple cognitive outcomes").**

Thanks for this constructive comment. I agree that the Discussion section contained some redundancy. I have reorganized and revised the Discussion.

Kindly refer to the Strengths and Limitations section in the Discussion.

**5. Minor Editorial Corrections:**

**• Page 16. Please revise the incomplete sentence: “Further screening was carried out according to the instrumental variable screening conditions and the final results are presented in Supplement II to IV and .” Clarify what supplement is missing or restructure the sentence for clarity.**

Thank you for pointing out the ambiguity in the sentence on page 16. I apologize for the confusion caused. There was no missing supplementary file; the issue was due to an incomplete sentence structure. I have revised this sentence. The corrected version now reads:

*“Further screening was conducted based on the instrumental variable selection criteria, and the final results are presented in Supplements II to IV.*”

Please refer to 3.1 Instrumental variable selection lines 3 to 5.

**• Review the manuscript for additional typographical and grammatical errors to improve clarity and readability. For instance, in the sentence on page 16 discussing the MR-Egger intercept test, consider revising to: "According to the MR-Egger intercept test, there was no evidence of horizontal pleiotropy (p > 0.05), indicating that the data were suitable for MR analysis."**

Thank you for your helpful suggestion. The manuscript was carefully reviewed, and minor typographical and grammatical errors were corrected to improve clarity and readability. Specifically, the sentence regarding the MR-Egger intercept test has been revised as suggested:

*“According to the MR-Egger intercept test, there was no evidence of horizontal pleiotropy (p > 0.05), indicating that the data were suitable for MR analysis.*”

Kindly refer to 3.3 Sensitivity analyses lines 4 to 6.

**Reviewer #2: This is a well-written manuscript contributing to the understanding of the relation between self-reported sleep duration and cognitive function. The study's design is robust and methodologically sound, and its results are well presented based on the following:**

Thank you very much for your kind and encouraging comments. I appreciate your positive feedback on the writing, study design, and presentation of the results. Below are responses to each of your comments. Revisions within the manuscript are highlighted in blue. Thank you again for taking the time to review this manuscript — I truly appreciate it.

1. **The author has clearly stated the aim of this study, which is based on a thorough evaluation of the contradictory findings of previous observational studies of the relation between sleep duration and cognitive decline, using different adjustment models.**

Thank you for your positive comment! I appreciate your recognition of the clarity of the study's aim.

2. **The study used large-scale GWAS studies with large sample sizes, which enhances its statistical power.**

Thank you for highlighting this strength of this study.

3. **Although the author relied on data extracted from multiple GWAS studies, all these studies include individuals of European ancestry, which limits bias due to population differences.**

I appreciate your acknowledgment of the effort to reduce population stratification bias. As noted, all GWAS datasets included individuals of European ancestry, which helps minimize confounding due to ancestral heterogeneity. This study have clarified this point in the discussion as a strength, while also acknowledging it as a limitation regarding the generalizability of the findings to other populations. As written in discussion：

*“In addition, all participants in all GWAS datasets were of European origin, helping to reduce bias due to population stratification.*”

*“Third, the MR analyses were restricted to individuals of European ancestry, limiting the generalisability of the findings to other populations. Causal relationships may vary across genetic backgrounds, highlighting the importance of future studies in more diverse cohorts.*”

Kindly refer to the third point of the strengths and the third point of limitations of the Discussion.

4. **The instrumental variables, SNPs, used in the study were thoroughly selected after considering linkage disequilibrium, strand orientation consistency, and checking their association with other genomic traits to exclude confounding factors.**

Thank you for recognizing the rigor of our instrumental variable selection process. This comprehensive approach helps ensure the validity of the Mendelian randomization assumptions.

5. **Compared to previous literature which relied on the use of Alzheimer Disease as a cognitive outcome, this study is comprehensive. It is based on the evaluation of a broad range of cognitive outcomes, including 4 types of dementia and 7 cognitive tests. Each test comprises distinct subtests, scoring mechanisms, and participant demographics.**

Many thanks for this encouraging comment! Indeed, one of the main contributions of this study is the comprehensive assessment of cognitive outcomes. By including multiple dementia subtypes and diverse cognitive tests, this study aimed to capture a broader and more nuanced picture of cognitive function. This approach allows us to explore potential differences in the effects of sleep duration across various cognitive domains.

6. **The study uses several MR methods; IVW supplemented with MR-Egger regression and the Weighted Median approach, followed by multiple sensitivity analyses. The use of these methods is well presented in the Methodology, however, the explanation of how they support the robustness of the findings and address potential pleiotropy could be elaborated in the discussion.**

Sure thing! Done! I have expanded the Discussion section to elaborate on how each MR method and multiple sensitivity analyses contribute to the robustness of these findings and help address potential pleiotropy. The details are as follows：

*“In order to increase the robustness of the findings as well as to reduce the potential impact of horizontal pleiotropy, this study supplemented the main IVW analysis with MR-Egger and WME methods. The WME method provides valid causal estimates even when up to 50% of the IVs are invalid. The convergence of the results derived from the three methods enhances confidence in the validity of the findings and reduces the likelihood that the observed associations are attributable to pleiotropic bias. In addition, MR-Egger regression can detect directional pleiotropy through its intercept term, which reflects the average pleiotropic effect across IVs. The results suggest that the MR-Egger intercept is not statistically significant, suggesting limited evidence of horizontal pleiotropy. In addition, the MR-PRESSO detects potential pleiotropy by identifying and correcting for anomalous variation that may overly affect the results.The absence of significant outliers or large distortions in the MR-PRESSO supports the reliability of the estimates.*”

Kindly refer to the fourth point of limitations in Discussion.

7. **The study relies on Mendelian Randomization analysis to find causal relationships, since it alleviates bias from confounding and reverse causation. In line of this argument, the recent study on the Relationship of Sleep Duration on Cognitive Function Among the Elderly (Qiu et al., 2024) is worth mentioning. It combines data from the National Health and Nutrition Examination Survey (NHANES) and Mendelian randomization methods. The findings of this article could be also discussed in the discussion part.**

Thank you for your insightful suggestion. I have cited the study by Qiu et al. (2024) in the Discussion section. Their findings have been integrated with those from other Mendelian randomization studies focusing on sleep duration, insomnia, and cognitive function. The details are as follows：

*“Qiu et al. combined data from the National Health and Nutrition Examination Survey (NHANES) and MR analyses to report an inverted U-shaped relationship between sleep duration and cognitive function in older adults in a cross-sectional study, but the MR analyses did not find a causal effect of sleep duration and insomnia on cognitive function (59).*”

*“Five previous MR studies have brought enlightening evidence (57–61). Based on previous MR analyses, the present study further explored the causal relationship between sleep duration and cognitive functioning by further categorising sleep duration: long sleep duration and short sleep duration. In addition, the present study used insomnia GWAS data based on a larger sample size for exploration. A review of previous MR analyses on insomnia and cognitive functioning found that the study by Wang et al. used insomnia GWAS data from 386,533 individuals of European ancestry recruited from 22 assessment centres in the UK (58); the study by Huang et al. included insomnia GWAS data from 237,627 individuals of European ancestry in the UK (61). The present study used combined data collected by UK Biobank (n = 386,533 individuals) and 23andMe (n = 944,477 individuals) for a final sample size of up to 1,331,010 individuals of European ancestry. Insomnia studies with different sample sizes and study sites may yield different conclusions. On the other hand, Qiu et al.'s MR analyses considered only one test of cognitive function (59). Henry et al. judged cognitive function only by visual memory tests, reaction time and dementia or not (57). However, the manifestations of cognitive decline are complex. It is difficult to fully reflect changes in cognitive functioning and the presence of dementia using only one or two tests. Compared with the study of Henry et al, Wang et al included more cognitive tests such as cognitive performance, fluid intelligence (58). Based on previous studies, the present study added more cognitive function tests (seven cognitive tests including memory test, reaction time cognitive performance and fluid intelligence). At the same time, previous MR studies did not provide more evidence about sleep characteristics and dementia subtypes. Qiu et al.'s MR analysis did not consider any dementia and its subtypes (59); Henry et al., Yuan et al. and Huang et al. all focused only on AD, the most common form of dementia (57, 60, 61); and Wang et al.'s focus fell on AD-related progression scores and changes in brain structure (58). The present study extends beyond AD to more common subtypes of dementia, namely vascular dementia, Lewy body dementia, and frontotemporal dementia.*”

Kindly refer to the fifth and sixth paragraphs of the discussion.

8. **The fact that genetic predisposition may only explain part of the variance related to cognitive outcomes is acknowledged in the discussion but could be further elaborated.**

Thanks for pointing this out. Genetic susceptibility explains only a portion of the variation in cognitive outcomes, while a range of non-genetic factors have a significant impact on cognitive health and Alzheimer's disease risk. To elucidate this point more thoroughly, the discussion section has been expanded to emphasise this limitation. The details are as follows：

*“The polygenic structure of cognitive outcomes means that even large-scale GWAS can only capture part of the overall phenotypic variation (28, 79). Thus, the genetic tools used in MR studies explain only a limited portion of the complex causal pathways that influence cognition and dementia risk. An individual's genetic risk may only manifest itself in a particular context. For example, individuals with the same risk genes perform differently in cognitive development, in part due to differences in education or nutrition. MR analyses typically assume homogenous genetic effects, which may underestimate the true complexity of gene-environment interactions.*”

Please refer to the final point of limitations of the Discussion.

9. **While the author discusses the effect of sleep duration on Aβ deposition and clearance, the discussion could acknowledge potential biological mechanisms that explain the causal relationships, such as the relation between sleep quality, neuroinflammation and cognitive health.**

Thank you for your valuable suggestion. The discussion section has been revised to further elaborate on potential biological mechanisms that may explain the observed causal associations. In particular, the point regarding the relationship between sleep quality, neuroinflammation, and cognitive health has been incorporated. Kindly refer to the third paragraph in the Discussion. I have added the following:

*“Beyond sleep duration, growing evidence suggests that sleep quality plays a critical role in brain health through its effects on neuroinflammation and glymphatic clearance. Disrupted or fragmented sleep has been shown to increase systemic and central nervous system levels of pro-inflammatory cytokines, such as interleukin-6 (IL-6) and tumor necrosis factor-alpha (TNF-α), which can exacerbate neuroinflammatory processes and contribute to neurodegeneration (48). Inadequate or poor-quality sleep may also impair the function of the glymphatic system—a waste clearance pathway active during sleep—thereby reducing the removal efficiency of amyloid-β and other neurotoxic proteins from the brain (47). Chronic neuroinflammation has been implicated in synaptic dysfunction, tau pathology, and accelerated cognitive decline, suggesting that sleep quality is a key modifiable factor in the pathway linking sleep disturbance to AD (49). These biological mechanisms highlight the importance of considering both the duration and quality of sleep when evaluating its causal role in cognitive aging and neurodegenerative disease.”*

**Reviewer #3: The manuscript focuses on the self -reported measures of sleep durations with the cognition, using the genetic variations as indirect causative factors in losing the mental abilities.**

Thank you for taking the time to review this manuscript. I appreciate your comments and the opportunity to clarify and improve this work. Revisions made in the manuscript are highlighted in blue.

**This study may exhibit several limitations:**

1. **The genetic variations in the SNPs determined by this study were already related to loss mental abilities by dementia or Alzheimer, therefor, the reliance to self-reporting duration to measure the cognitive may be not accurate and lead to biased results.**

Thank you for your thoughtful comment. I fully understand your concern. However, the SNPs used in this MR analysis are derived from large genome-wide association studies (GWAS) that focus on identifying variants associated with sleep duration or insomnia rather than cognitive impairment or Alzheimer's disease. Meanwhile, during the SNP screening stage, this study further conducted a comprehensive search in the PhenoScanner database so as to exclude potential confounders affecting cognitive outcomes. This helps to reduce the risk of reverse causation or confounding due to pre-existing cognitive decline.

On the other hand, one important limitation of the present study is the reliance on self-reported sleep duration, which may be susceptible to error due to memory bias and reporting bias. As written in the discussion section:

“*Self-reported sleep duration exhibits inaccuracies attributable to memory bias and reporting bias, potentially encompassing additional facets of sleep beyond mere duration. The absence of a flawless method for measuring sleep duration without disrupting daily routines underscores the challenge in this domain (56). Despite the often robust correlation between actigraphy and polysomnography findings (72), actigraphy tends to overestimate sleep duration, particularly beyond the confines of sleep laboratory environments (72-76). Notably, a study revealed consistent genetic associations with sleep duration regardless of whether it was measured via actigraphy or self-reports (16). Given this, and the fact that international guidelines predominantly rely on self-reported measures for defining sleep duration recommendations (2, 77), self-report remains the most relevant metric for clinical, public health, and policy contexts, despite its known limitations.* ”

Kindly refer to the first point of limitations of the Discussion.

2. **The study restricted the randomized analysis on the European ancestry population. So, the results may not match with variation in another ancestry populations.**

Thank you for highlighting this important point. Indeed, this analyses were restricted to individuals of European ancestry to minimise the potential for population stratification and ensure the validity of the genetic instruments used in the MR analysis. As written in the Discussion in strengths:

“*In addition, all participants in all GWAS datasets were of European origin, helping to reduce bias due to population stratification.*”

At the same time, this study recognises the limitations posed by the restriction of the study to populations of European origin, as written in limitations of the Discussion:

“*Third, the MR analyses were restricted to individuals of European ancestry, limiting the generalisability of the findings to other populations. Causal relationships may vary across genetic backgrounds, highlighting the importance of future studies in more diverse cohorts.*”

3. **The genetic variations influence sleep duration is unclear and affected by several environmental factors, sex, age …etc. therefor it’s not enough to be decisive results to link the cognitive with sleep duration.**

Thank you for this important comment. While it is true that sleep duration is influenced by a combination of genetic and environmental factors such as age, sex, and lifestyle, MR analysis is specifically designed to address issues of confounding and reverse causation that often limit observational studies. MR uses genetic variants as instrumental variables (IVs), which are randomly allocated at conception and thus less likely to be influenced by environmental factors or disease status later in life.

Horizontal pleiotropy occurs when a genetic variant used as an instrumental variable (IV) affects the outcome through pathways (such as environmental factors) other than the sleep duration. In order to increase the robustness of the findings and to reduce the potential impact of horizontal pleiotropy, this study supplemented the main inverse-variance weighted (IVW) analysis with additional methods, including MR-Egger regression and the weighted median estimator (WME). The WME method is particularly useful as it can provide valid causal estimates even when up to 50% of the IVs are invalid. The consistency of the results across these three methods enhances confidence in the validity of the causal inference and reduces the likelihood that the observed associations are attributable to pleiotropic bias. Moreover, MR-Egger regression allows for the detection of directional pleiotropy through its intercept term, which reflects the average pleiotropic effect across the instruments. In this study, the MR-Egger intercept was not statistically significant, indicating limited evidence of horizontal pleiotropy. Further, MR-PRESSO was applied to detect and correct for potential outlier variants that may distort the causal estimates. The absence of significant outliers or distortions in the MR-PRESSO results supports the robustness and reliability of the findings.

Finally, it is acknowledged that genetic susceptibility explains only a portion of the variation in cognitive outcomes, while a wide range of non-genetic factors—including environmental, social, and behavioural influences—play significant roles in determining cognitive health. As written in the discussion section:

*“The polygenic structure of cognitive outcomes means that even large-scale GWAS can only capture part of the overall phenotypic variation (28, 79). Thus, the genetic tools used in MR studies explain only a limited portion of the complex causal pathways that influence cognition and dementia risk. An individual's genetic risk may only manifest itself in a particular context. For example, individuals with the same risk genes perform differently in cognitive development, in part due to differences in education or nutrition. MR analyses typically assume homogenous genetic effects, which may underestimate the true complexity of gene-environment interactions. Nonetheless, despite these limitations, MR offers a powerful tool for assessing causality in observational research. By leveraging genetic instruments, MR enables causal inference even in the face of incomplete heritability information. Taken together, these findings provide important evidence for the causal role of sleep traits in cognitive decline and dementia, while also highlighting the need for cautious interpretation and further validation in diverse populations and methodological frameworks. ”*

Kindly refer to the final point of limitations of the Discussion.

**Reviewer #4: Similar studies were published earlier concluding the same outcomes that prescribes a normal sleep duration of 7-8 hours in a day results in healthy cognitive function. However, this study has the uniqueness of implementing new MR strategy with a robust workflow that rules out some bias present in the previous studies especially with respect to the long sleep duration. Limitation of the study were discussed as well.**

Thank you for the insightful and supportive comment! The recognition of the study's methodological strengths and its contribution to the existing literature is greatly appreciated!

**Reference** **list**

(1) Walker MP. The Role of Slow Wave Sleep in Memory Processing. Journal of Clinical Sleep Medicine. 2009 Apr 15;5(2).
(2) Scullin MK, Gao C. Dynamic Contributions of Slow Wave Sleep and REM Sleep to Cognitive Longevity. Current Sleep Medicine Reports. 2018 Oct 23;4(4):284–93.
(3) Burgess S, Butterworth A, Thompson SG. Mendelian Randomization Analysis With Multiple Genetic Variants Using Summarized Data. Genetic Epidemiology. 2013 Sep 20;37(7):658–65.
(4) Davies NM, Holmes MV, Davey Smith G. Reading Mendelian randomisation studies: a guide, glossary, and checklist for clinicians. BMJ [Internet]. 2018 Jul 12;362:k601. Available from: https://www.bmj.com/content/362/bmj.k601
