# Supplementary material for: Mendelian randomization study of self-reported long sleep duration, short sleep duration, and insomnia and cognitive function
Source: PLoS One. 2025 Aug 20;20(8):e0330782. doi: 10.1371/journal.pone.0330782 (PMC12367179; doi:10.1371/journal.pone.0330782)
Supplement: Attachment — Submitted filename: Response to Reviewers_R2.docx [file pone.0330782.s006.docx]

PONE-D-24-46332R2

Mendelian Randomization Study of Self-reported Long sleep duration, Short sleep duration, and Insomnia and Cognitive Function

Thank you for the opportunity to revise and resubmit the manuscript. I appreciate the reviewers’ constructive feedback, which has helped us improve the clarity and quality of the work. I have addressed all comments carefully and outlined the responses in the accompanying revision memo. I hope the revised manuscript meets the standards for publication in PLOS ONE.

**Editor**

**Thank you for your response. We still require accession numbers so that interested researchers may search up your exact datasets that have been used. Currently, the links do not lead directly to the datasets and instead lead to the homepages of the websites.**

I have removed the general website homepage links from the manuscript—the sentence "Genetic data pertaining to cognitive function were sourced from the UK Biobank (https://www.ukbiobank.ac.uk) and the FinnGen consortium (https://www.finngen.fi/fi)" has been deleted. In addition, I have added direct links to the specific datasets used in S1 Table, so that interested researchers may easily access the exact data.

**Reviewer #1: Thank you for your thorough and thoughtful responses to my initial review. I am pleased to confirm that all of my concerns have been fully addressed in the revised manuscript.**

**The additions to the Results and Discussion sections offer a much clearer interpretation of the data, particularly regarding the distinct associations of short sleep duration, insomnia, and long sleep duration with cognitive outcomes. The clarification of potential biological mechanisms, especially in terms of sleep architecture and inflammatory pathways, adds valuable context to the findings. The inclusion of relevant literature (e.g., Wang et al., 2023) and the improved discussion flow also strengthen the overall narrative and support the manuscript’s contribution to the Mendelian randomization literature.**

**Figures have been referenced appropriately, editorial issues have been resolved, and the limitations of the MR design—including instrument strength and sleep phenotype definitions—are now well-articulated.**

**I have no further suggestions for revision. The manuscript is methodologically sound, clearly written, and meets the standards for publication. I recommend acceptance in its current form.**

Thank you for the positive and encouraging feedback. It is reassuring to know that the revisions effectively addressed the previous concerns.

Thank you again for the thoughtful review and recommendation for acceptance.

**Reviewer #5: This is a well-written manuscript exploring the causal associations between sleep hours and cognitive functions using Mendelian randomization. The paper has clearly stated how the statistical analyses were conducted and summarized the strength and limitations of the methods.**

Thank you for the positive feedback！

**Reviewer #6: This study investigates whether sleep patterns, specifically short or long sleep duration and insomnia, causally influence cognitive performance and dementia risk. The researchers employed Mendelian Randomization to leverage genetic variants as instrumental variables for testing causal relationships. They found that genetically predicted short sleep duration impairs cognitive performance across multiple domains including memory and attention. Insomnia showed a causal association with Alzheimer's disease risk but did not affect general cognitive test performance. Long sleep duration effects remain inconclusive due to insufficient genetic instruments.**

Thank you for your thorough and professional review of the manuscript. As you are concerned, there are several problems that need to be addressed. I have addressed and explained each of your concerns, and I believe that the revised manuscript is stronger as a result. Below are responses to each of your comments. Revisions within the manuscript are highlighted in blue.

**Major Comments**

**1. Insufficient Statistical Power for Long Sleep Duration Analysis: Genetic instrument for long sleep duration contains only 7 SNPs, severely limiting statistical power. The abstract and discussion imply absence of causal effects rather than acknowledging inadequate power to detect them. Revise language throughout to clarify that findings reflect limited instrumental strength rather than definitive evidence against causal effects.**

Thank you for highlighting the important issue of limited statistical power in the analysis of long sleep duration. The concern regarding the small number of SNPs (n=7) used as the genetic instrument is fully acknowledged. To address this, the language in the Abstract, Results, and Discussion sections has been revised to clarify that the null findings for long sleep duration should be interpreted with caution, reflecting the limited strength of the instrument rather than definitive evidence against a causal relationship.

In the Abstract, the corresponding section is written as:

“*However, due to the limited number of SNPs (n = 7) available as instruments for long sleep duration, there was no strong evidence to support a causal effect of long sleep duration on cognitive outcomes.*”

“*The evidence of causality between long sleep duration and cognitive function requires further investigation.*”

In the Results, the corresponding section is written as:

“*Considering the limited number of SNPs, the causal relationship between long sleep duration and cognitive function still needs further investigation.*”

In the Discussion, the corresponding section is written as:

“*The evidence of causality between long sleep duration and cognitive function remains unclear and requires further investigation.*”

“*Considering the limited number of SNPs (n = 7) available as instruments for long sleep duration, there was no strong evidence to support a causal effect of long sleep duration on cognitive outcomes.*”

In the Conclusions, the corresponding section is written as:

“*However, due to the limited number of SNPs available as instruments for long sleep duration, the evidence for a causal relationship between long sleep duration and cognitive function remains inconclusive and warrants further investigation.*”

Please refer to the last sentence of the results and the second sentence of the discussion in the Abstract, the last sentence of 3.2 Primary analysis in the Results, the last sentence of the first paragraph and the first sentence of the fourth paragraph in the Discussion and the last sentence in the Conclusions.

**2. Disconnect Between Insomnia Effects on AD vs. Cognitive Performance: Your finding that insomnia causally increases Alzheimer's risk while showing no effect on cognitive test scores requires deeper exploration. The brief discussion doesn't adequately address this apparent contradiction. Expand discussion of potential mechanisms underlying this dissociation, considering whether UK Biobank cognitive assessments may lack sensitivity to detect subtle neurodegenerative changes that manifest clinically as AD.**

Thank you for highlighting the important point. The Discussion section has been expanded to explore potential mechanisms underlying this discrepancy. Specifically, I wrote:

“*The present study suggests a close causal link between insomnia and AD, rather than cognitive test scores. There are several plausible explanations for this dissociation. Firstly, cognitive assessment methods provided by the UK Biobank may not readily detect cognitive decline in the preclinical stages of AD. These assessment methods, although suitable for large-scale population studies, may lack the sensitivity to capture subtle or domain-specific cognitive deficits, such as language deficits, disorientation disorders, which are often among the areas susceptible to AD (24, 40). Thus, individuals with early neurodegenerative conditions may still perform normally on these relatively crude tests. Secondly, insomnia may increase long-term risk for AD through pathways that do not lead to immediately measurable cognitive deficits. For example, insomnia is associated with impaired clearance of amyloid β-protein and tau protein by the lymphatic system, systemic inflammation, and neuroendocrine dysregulation, all of which are implicated in the pathogenesis of AD but may precede observable changes in cognitive performance by years or even decades (44, 46, 49). Thirdly, individuals with chronic insomnia may also develop compensatory mechanisms or cognitive reserves that temporarily buffer measurable performance deficits, thereby delaying the emergence of impairments on cognitive tests (64). Finally, in the GWAS included in this study, the diagnosis of AD captures a clinical endpoint, whereas the cognitive performance indicators in the UK Biobank reflect subclinical characteristics. The lack of association between insomnia and cognitive test scores does not necessarily imply a lack of cognitive impact, but should reflect the complexity and time lag between sleep disorders and cognitive decline.*”

Kindly refer to the ninth paragraph of the Discussion section.

**3. Self-Report Bias in Sleep Phenotypes: Reliance on self-reported sleep data introduces potential misclassification bias that could attenuate true causal estimates. Subjective sleep perception varies considerably between individuals, particularly for insomnia symptoms. Discuss how measurement error in self-reported exposures might bias results toward the null and emphasize the need for future studies incorporating objective sleep measurements.**

Thanks for pointing this out. To address this concern, I have revised the manuscript to discuss the implications of using self-reported exposures and emphasized the need for future studies using objective sleep measures. The details are as follows:

“*First, self-reported sleep duration or insomnia symptoms may introduce a potential misclassification bias due to memory and reporting errors, which can lead to biased MR analysis estimates towards zero values and underestimation of true causal effects. This bias mainly occurs in cases where there is no difference in measurement error. In this case, the noise introduced by self-reporting weakens the observed association between the genetic IV and the exposure, thereby reducing the strength of the IV. This dilution effect reduces the proportion of exposure variance explained by the IVs, leading to regression dilution bias. As a result, the estimated causal effect of sleep on cognitive or neurological outcomes is attenuated, making it appear weaker or even zero, despite the fact that there may be a genuine underlying association. This issue is particularly important for subjective sleep phenotypes such as insomnia, as individual perceptions can vary widely depending on factors such as mood, stress, and attentional biases. In addition, an increase in exposure misclassification reduces statistical efficacy and widens standard errors, thereby increasing the likelihood of Type II errors. The absence of a flawless method for measuring sleep duration without disrupting daily routines underscores the challenge in this domain (56). Despite the often robust correlation between actigraphy and polysomnography findings (72), actigraphy tends to overestimate sleep duration, particularly beyond the confines of sleep laboratory environments (72–76). Notably, genetic correlations with sleep duration appear to be consistent across self-reported and accelerometer-based measures (16), which provides some robustness to the use of self-reported data. In addition, international sleep guidelines continue to rely on self-reported sleep duration to formulate public health recommendations (2, 77), which supports their relevance in both clinical and epidemiological contexts. Nonetheless, future research should prioritise the integration of objective sleep measures (e.g., somatokinetic recorders and polysomnography) into large-scale cohort studies to improve measurement accuracy and reduce potential bias in causal inference.* ”

Please kindly refer to the eleventh paragraph of the Discussion section, the first point under the limitations.

4. **Mechanistic Discussion Lacks Domain Specificity: Your biological discussion mentions relevant pathways but fails to connect these mechanisms to your domain-specific cognitive findings. Link specific sleep-related mechanisms to affected cognitive domains, discuss how REM sleep disruption impacts attention and executive function while slow-wave sleep disruption affects memory consolidation.**

Thank you for this insightful comment. I have revised the Discussion section to include domain-specific links between sleep physiology and cognitive functions. I wrote:

“*In the current study, the genetic association analyses yielded some interesting results. Short sleep duration seems to be more strongly associated with certain cognitive indicators, such as fluid intelligence, than insomnia. This may be due to differences in the definitions of short sleep duration and insomnia. In this study, short sleep duration was defined as 6 hours or less of sleep per 24 hours. Shorter sleep duration implies a reduction in slow wave sleep (SWS) and rapid eye movement (REM) sleep. These two sleep stages play different roles in cognitive functioning. SWS, characterised by high-amplitude, low-frequency brain waves, is essential for the hippocampal-neocortical dialogue during memory consolidation (62). Diminished SWS impairs the reactivation of newly acquired information from the hippocampus and its transfer to long-term storage in the cortex (62). Thus, SWS is critically associated with declarative memory consolidation and fact-based situational memory (62). It has been shown that SWS disruption is associated with impaired situational memory and slower information processing, especially after accounting for demographic and lifestyle factors (62). In contrast, REM sleep is known to promote synaptic plasticity and activate neural circuits involving the prefrontal cortex and striatum to support emotion regulation, attention, and executive function. REM sleep disruption has been shown to impair sustained attention, working memory, and cognitive flexibility, functions that are critically dependent on the integrity of frontal-striatal circuits (63). The UK Biobank assessment reflects fluid intelligence - which encompasses the ability to process and apply new information, reason abstractly, and perform goal-directed behaviours effectively. Therefore, the observed association between short sleep duration and reduced fluid intelligence scores may reflect cumulative deficits caused by reduced SWS, which affects memory encoding and consolidation, and reduced REM sleep, which affects attention and executive functioning.* ”

“*Insomnia is defined as difficulty falling asleep at night or waking up in the middle of the night. Thus, insomniacs may still obtain a normal total sleep duration, albeit with poorer sleep quality. Alternatively, insomniacs may be more conscious of this compensation through planned daytime naps, but chronically sleep-deprived individuals tend to underestimate their cognitive deficits. During planned daytime naps, individuals may not only compensate for REM sleep, but also compensate for reduced SWS by prolonging REM sleep, thus demonstrating resilience to SWS deficits. From this perspective, some of the functions of SWS sleep may be redistributed during REM sleep stages or compensated to some extent by REM sleep-related processes (63). Second, sleep duration in this study was supported by accelerometer estimates. Insomnia relies on subjective self-reports, and subjective symptoms are influenced by mood, anxiety, and personal distress, which complicates its connection to objective cognition. In contrast to disease diagnosis, fluid intelligence emphasises speed of information processing, ability to reason abstractly and executive function. Thus, fluid intelligence scores better capture the changes in cognitive functioning that occur as a result of reduced SWS and REM due to short sleep duration.*”

Kindly refer to the seventh and eighth paragraphs of the Discussion section.

**5. Overstated Novelty Claims: The introduction presents this as the first MR study of sleep-cognition relationships, but you later cite several relevant prior MR studies. This inconsistent framing undermines credibility. Refine novelty claims to highlight genuine contributions: comprehensive cognitive phenotyping, inclusion of multiple dementia subtypes, and largest sample size for insomnia analysis.**

Many Thanks for pointing this out. I have deleted inappropriate expressions such as “*To the best of current knowledge,* *this is the first MR study to investigate causal associations between long sleep duration, short sleep duration, insomnia and multiple cognitive outcomes.*”

**6. Limited Generalizability Beyond European Ancestry: Restricting analyses to European ancestry populations limits generalizability, particularly given known ancestry differences in sleep patterns and dementia risk profiles. Explicitly discuss this limitation's public health implications and emphasize the need for replication in diverse populations before clinical translation.**

I fully acknowledge that limiting this analyses to individuals of European ancestry reduces the generalizability of the findings. I have described this limitation in the Discussion section, noting that sleep characteristics and dementia risk profiles vary across ancestral groups (Chen et al., 2015; Mester et al., 2023). This limits the immediate public health applicability of the results to non-European populations. I have emphasized the need for future studies to replicate these findings in more diverse cohorts before any clinical translation can be considered. The details are as follows：

“*Third, the MR analyses were restricted to individuals of European ancestry, limiting the generalisability of the findings to other populations. This is a notable limitation given the significant differences in sleep patterns, prevalence of sleep disorders, and dementia risk profiles across ethnic groups. For example, research suggests that individuals of African, Asian, and Hispanic ancestry may have different circadian rhythms, sleep duration distributions, and susceptibility to sleep-related health outcomes compared with individuals of European ancestry (79). Similarly, genetic architecture (including allele frequencies and patterns of linkage disequilibrium) may differ significantly across populations, which may alter the strength or direction of causal relationships (80). If causal pathways vary by descent, clinical recommendations based on European data alone may not be used in all populations. Future studies should consider validation in cohorts of diverse ancestry.* ”

Kindly refer to the eleventh paragraph of the Discussion section, the third point under the limitations.

**Minor Comments:**

1. **Sensitivity analyses are comprehensive and well-executed, strengthening confidence in the primary findings.**

Thank you!

2. **Effect sizes for some cognitive outcomes, while statistically significant, appear small. Providing clinical context or benchmarks would help readers assess practical significance.**

Absolutely, done! In the revised submission, I added the following:

“*Despite the small amount of effect observed, MR analyses support that short sleep duration performs equally poorly on other cognitive tests. Specifically, genetically predicted short sleep duration was causally associated with reduced memory performance (beta = –0.10, 95% CI: –0.20 to –0.0005, P = 0.04). This result further highlights the detrimental effects of reduced sleep duration on memory encoding and recall. In addition, impaired executive functioning due to short sleep duration was also reflected in poorer performance on the TM test. Individuals with genetically predicted short sleep duration exhibited longer step intervals (beta = 0.11, 95% CI: 0.01 to 0.21, P = 0.03) and longer total task duration (beta = 0.11, 95% CI: 0.002 to 0.22, P = 0.04), indicating impaired attentional switching and cognitive flexibility. Considering accumulation over time, even mild cognitive decline (e.g., 0.2–0.3 standard deviations) may predict increased risk of functional decline and dementia in later life (52, 54, 63).*”

Kindly refer to the last six sentences of the seventh paragraph of the Discussion section.

3. **Consider adding brief justification for your sleep duration thresholds (<6h, >9h) in the Methods section.**

Sure thing! I have added a brief justification in the Methods section, noting that the thresholds of <6 hours and >9 hours were based on prior epidemiological research that defines these cut-offs as clinically meaningful for short and long sleep duration (Hirshkowitz, 2015; Dashti et al., 2019). These thresholds have been linked to increased risk of adverse outcomes, including cognitive impairment and dementia (Hirshkowitz, 2015). I wrote:

“*These thresholds were chosen based on previous epidemiological and clinical studies that have shown that both less than 6 hours and more than 9 hours of sleep are associated with adverse health outcomes, including increased risk of cardiovascular disease, metabolic dysfunction, and cognitive decline (3, 16).*”

Kindly refer to 2.2.1 Long sleep duration, short sleep duration, and insomnia.

**4. Discussion flow has improved but could benefit from eliminating remaining redundancy between cognitive and dementia sections.**

Done!

**Reference** **list**

Chen, X., Wang, R., Zee, P., Lutsey, P.L., Javaheri, S., Alcántara, C., Jackson, C.L., Williams, M.A. and Redline, S. (2015). Racial/Ethnic Differences in Sleep Disturbances: The Multi-Ethnic Study of Atherosclerosis (MESA). SLEEP, 38(6). doi:https://doi.org/10.5665/sleep.4732.

Dashti, H.S., Jones, S.E., Wood, A.R., Lane, J.M., van Hees, V.T., Wang, H., Rhodes, J.A., Song, Y., Patel, K., Anderson, S.G., Beaumont, R.N., Bechtold, D.A., Bowden, J., Cade, B.E., Garaulet, M., Kyle, S.D., Little, M.A., Loudon, A.S., Luik, A.I. and Scheer, F.A.J.L. (2019). Genome-wide association study identifies genetic loci for self-reported habitual sleep duration supported by accelerometer-derived estimates. Nature Communications, [online] 10(1), pp.1–12. doi:https://doi.org/10.1038/s41467-019-08917-4.

Hirshkowitz, M. (2015). National Sleep Foundation’s Sleep Time Duration recommendations: Methodology and Results Summary. Sleep Health, [online] 1(1), pp.40–43. doi:https://doi.org/10.1016/j.sleh.2014.12.010.

Mester, R., Hou, K., Ding, Y., Meeks, G., Burch, K.S., Bhattacharya, A., Henn, B.M. and Bogdan Pasaniuc (2023). Impact of cross-ancestry genetic architecture on GWASs in admixed populations. American journal of human genetics, 110(6), pp.927–939. doi:https://doi.org/10.1016/j.ajhg.2023.05.001.
